# Description of ultrastrong light–matter interaction through coupled harmonic oscillator models and their connection with cavity-QED Hamiltonians

**DOI:** 10.1515/nanoph-2024-0528

**Published:** 2025-03-11

**Authors:** Unai Muniain, Javier Aizpurua, Rainer Hillenbrand, Luis Martín-Moreno, Ruben Esteban

**Affiliations:** Donostia International Physics Center, Paseo Manuel de Lardizabal 4, 20018 Donostia-San Sebastián, Spain; IKERBASQUE, Basque Foundation for Science, María Díaz de Haro 3, 48013 Bilbao, Spain; Department of Electricity and Electronics, University of the Basque Country (UPV/EHU), 48940 Leioa, Spain; CIC nanoGUNE BRTA, Tolosa Hiribidea 76, 20018 Donostia-San Sebastián, Spain; Instituto de Nanociencia y Materiales de Aragón (INMA), CSIC-Universidad de Zaragoza, 50009 Zaragoza, Spain; Departamento de Física de la Materia Condensada, Universidad de Zaragoza, 50009 Zaragoza, Spain; 202635 Centro de Física de Materiales (CFM-MPC), CSIC-UPV/EHU, Paseo Manuel de Lardizabal 5, 20018 Donostia-San Sebastián, Spain

**Keywords:** quantum nanophotonics, ultrastrong coupling, transverse and longitudinal fields, Coulomb coupling, Reststrahlen band, nanocavities

## Abstract

Classical coupled harmonic oscillator models are capable of describing the optical and infrared response of nanophotonic systems where a cavity photon couples to dipolar matter excitations. The distinct forms of coupling adopted in these classical models lead to different results in the ultrastrong coupling regime. To clarify the specific classical model required to address particular configurations, we establish a connection between each oscillator model and the equivalent cavity Quantum Electrodynamics description. We show that the proper choice of coupled harmonic oscillator model depends on the presence or absence of the diamagnetic term in the quantum models, linked to whether transverse or longitudinal electromagnetic fields mediate the coupling. This analysis also shows how to exploit the classical oscillator models to extract measurable information of the optical response, as demonstrated in three canonical photonic systems, and to describe the opening of the Reststrahlen band in the bulk dispersion of phononic materials.

## 1 Introduction

The optical properties of molecules, quantum dots, two-dimensional materials, or other systems supporting matter excitations are strongly modified when these excitations are coupled to the electromagnetic modes of a cavity or a resonator. The strong coupling regime is reached when the coupling strength *g* between the cavity modes and the matter excitations exceeds their losses [1], [2]. In this regime, hybrid modes known as polaritons emerge, exhibiting modified frequencies and new properties as compared to the uncoupled constituents. Strongly coupled system can also exhibit effects beyond the classical realm, including nonlinearities due to the Jaynes–Cummings ladder [3], emission of strongly correlated light [4], and changes on the chemical reactivity [5] or on the conductivity [6] of molecules located inside the cavity.

After the first observations of strong coupling for a single [7], [8] and many emitters [9], [10], [11], very large coupling strengths have been successfully measured in subsequent experiments, exploiting semiconductors [12], [13], superconducting circuits [14], plasmonic nanoparticle crystals [15], or ensembles of organic molecules [16], [17], [18], [19], for instance. It is now possible to reach coupling strengths that are several times larger than the threshold that usually marks the onset of the ultrastrong coupling regime [20], [21], [22], which roughly occurs when the coupling strength is ≈ 10 % of the uncoupled cavity mode and matter excitation resonant frequencies. In this ultrastrong coupling regime, additional quantum effects emerge, such as a shift of the ground state energy and the appearance of virtual excitations in this state [23], which cannot be accounted for within the rotating-wave approximation (RWA).

Models based on the Cavity Quantum Electrodynamics (cavity-QED) framework offer a natural description of these effects. However, two different QED Hamiltonians have been considered when studying the ultrastrong coupling regime, with differences stemming from the presence or absence of a contribution to the energy, the so-called diamagnetic term (also known as the *A* ^2^ term, with *A* the transverse vector potential of the electromagnetic mode). Introducing this term avoids a superradiant phase transition [24], for example. However, the inclusion of the diamagnetic contribution is still under discussion [25], [26], [27], [28], [29] and depends on the specifics of the system [30], [31]. Furthermore, in the presence of a diamagnetic term, if the Hilbert space must be truncated when performing the calculations (as is often the case), care needs to be taken as the results can become dependent on the chosen gauge [32], [33].

On the other hand, the response of nanophotonic systems in the strong and ultrastrong coupling regime is often described using phenomenological classical models based on coupled harmonic oscillators [34], [35], [36]. Such a simple description turns out to be adequate when the optical cavity couples with many quantum emitters (such as molecules, quantum dots, color centers in diamond…) or with matter excitations in an extended material. In this case, the nonlinearities behind many quantum effects are strongly attenuated compared to the single-emitter scenario. Here, we focus on nanophotonic systems for simplicity, but the discussion presented in this work is also valid for systems of micrometer dimensions unless otherwise stated. The classical coupled harmonic oscillator models have successfully described phenomena such as the avoided crossing of the hybrid modes [37], Fano resonances [38], stimulated Raman scattering [39], and electromagnetically induced transparency [40], [41], [42]. They are used to fit experimental data and to extract the coupling strength *g*, the frequencies of the hybrid modes, and the fraction of light and matter corresponding to each mode [43], [44]. However, in these phenomenological models, it is often unclear which exact physical quantity each oscillator represents, making it difficult to determine the value of a given observable in an experiment. To further complicate the situation, and similarly to the coexistence of cavity-QED Hamiltonian descriptions with and without diamagnetic term, different classical oscillator models have been used to analyze coupled systems, in both the strong and ultrastrong coupling regimes. In some models, the coupling terms are proportional to the amplitudes of the harmonic oscillators, while in others, they are proportional to the time derivatives of the amplitudes. The choice of coupling terms and the connections with the cavity-QED description are often not clearly justified [36], [45], [46], [47].

In this work, we first present a cavity-QED model describing the emitter-cavity coupling and derive several classical harmonic oscillator models that reproduce the same spectral properties and expectation values of any operator. These classical descriptions feature coupling terms that are proportional either to the amplitudes of the harmonic oscillators or to their time derivatives, accompanied by corresponding coupling-induced dressing of the oscillator frequencies. The choice of description is, in principle, a matter of preference. However, this flexibility disappears if one requires that the cavity frequencies in the phenomenological model are the (nondressed) bare ones, which is the standard choice in nanophotonics, where bare cavity frequencies can be measured or computed. Specifically, the presence or absence of the diamagnetic term in the original cavity-QED Hamiltonian determines the form of the coupling term in the classical model with bare cavity frequencies. We illustrate this scenario using several standard nanophotonic systems as examples. Furthermore, these examples serve to clarify how the amplitude of the oscillator modes relates to physical observables, such as the electric field within the cavity.

The paper is organized as follows:

In Section 2, we analyze in detail the connection between the cavity-QED descriptions and several equivalent classical harmonic oscillator models that can be derived from them.

**Figure 1:**
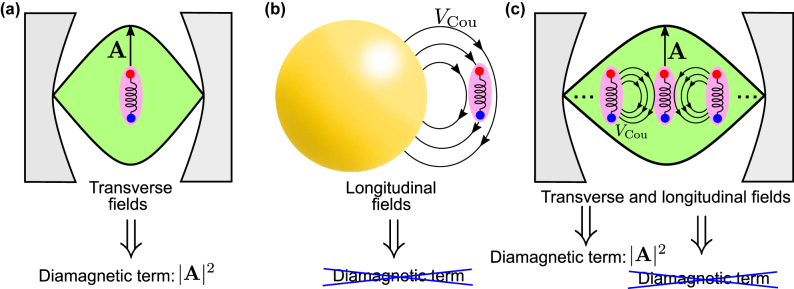
Schematics of the interaction between matter excitations and cavity modes in the three systems considered in this work. (a) A molecular emitter (as a representative quantum emitter) placed inside a dielectric (Fabry–Pérot) cavity. The transverse field of the single cavity mode considered is described with the vector potential **A**, which leads to the presence of the diamagnetic term ∝|**A**|^2^ in the cavity-QED Hamiltonian that describes this system. (b) A molecular emitter close to a metallic spherical nanoparticle and coupled to a single plasmonic mode. Within the quasistatic approximation, the molecular emitter only interacts with the longitudinal fields of the spherical nanoparticle, via the Coulomb potential *V*_Cou_. Since the vector potential **A** is not considered, the diamagnetic term is absent in the corresponding cavity-QED description. (c) An ensemble of molecular emitters placed inside a Fabry–Pérot cavity. The molecular emitters behave as a homogeneous bulk material. In this system, each emitter interacts with the transverse cavity mode characterized by the vector potential **A** as well as with the longitudinal fields associated with the Coulomb potential *V*_Cou_ induced by the other molecular emitters. Whereas the interaction of each emitter with cavity modes requires a diamagnetic term in the cavity-QED description, the coupling with other emitters is described without this term.

In Section 3, we apply these results to three canonical situations arising in nanophotonics: (i) a molecular emitter (or another quantum emitter) coupled to a conventional dielectric cavity (a Fabry–Pérot cavity, Figure 1(a)), (ii) a molecular emitter coupled to a small metallic nanoparticle supporting plasmonic resonances (Figure 1(b)), and (iii) an ensemble of molecular emitters or a homogeneous material inside a Fabry–Pérot cavity (Figure 1(c)). These examples emphasize the importance of the type of coupling. The choice of the classical coupled harmonic model (which depends on the presence of the diamagnetic term in the cavity-QED Hamiltonian) depends on whether the coupling is mediated by the transverse fields in a dielectric cavity or by the Coulomb interaction. Additionally, we demonstrate that identifying the amplitudes of the classical harmonic oscillators with the expectation values of quantum operators allows for the calculation physical observables within the classical description. Last, we use the third canonical configuration to discuss the bulk dispersion of materials and the emergence of the Reststrahlen band within harmonic oscillator models, a point discussed in more detail in the Supplementary Material.

## 2 Comparison of classical and cavity-QED models

In this section, we examine first a cavity-QED Hamiltonian that describes the interaction between a quantum emitter and a cavity optical mode. In Section 2.1, we derive the Heisenberg equations of motion for the displacements of the quantum operators, which take the form of classical oscillator equations. We present two equivalent descriptions, related by unitary transformations of the original quantum Hamiltonian. In one description, the coupling term between the oscillators is proportional to their amplitudes, while in the other, it is proportional to their time derivatives. Both approaches yield the same results, as the coupling strength and cavity frequency are appropriately renormalized in each case.

In nanophotonics, bare cavity frequencies, which can be measured or computed, are typically used when fitting experimental and simulated spectra, without considering their potential renormalization. We, therefore, focus on classical models with un-renormalized cavity frequencies, referring to them as the Spring Coupling (SpC) model for amplitude-based coupling, and the Momentum Coupling (MoC) model for coupling based on time derivatives of the amplitudes.

For specific values of the diamagnetic term in the Hamiltonian, the Heisenberg equations align naturally with either the SpC (Section 2.2) or MoC (Section 2.3) models, making each of them the most appropriate choice for fitting different experimental data. Section 2.4 illustrates the differences between these two models.

### 2.1 Derivation of the classical models from the Hamiltonians

In this subsection, we introduce the classical harmonic oscillator models. To this purpose, we first analyze the light–matter interaction using the cavity-QED framework. The cavity modes and the matter excitations are quantized using bosonic operators. The use of bosonic operators is valid for the cavity modes, and for matter excitations such as vibrations or phonons associated with a potential with a harmonic dependence on the degrees of freedom. The correspondence with classical harmonic oscillators (and thus the use of bosonic operators) is also valid to treat the coupling with matter excitations of fermionic nature provided that the number of excitations is much smaller than the number of quantum emitters (molecules, quantum dots…) and that any other effects induced by the saturation of the fermionic states can be discarded. Under these conditions, for example, the Quantum Rabi model (a generalization of the Jaynes–Cummings model to the ultrastrong coupling regime that includes a fermionic excitation [21]) becomes analogous to an appropriate bosonic Hamiltonian with a single matter excitation. Under this prescription based on bosonic operators, we can use a Hopfield-type Hamiltonian [48] in the form

(1) $$\begin{array}{ll} {\hat{H}}_{1} & =\hbar \omega_{\text{cav}}\left({\hat{a}}^{†}\hat{a}+\frac{1}{2}\right)+\hbar \omega_{\text{mat}}\left({\hat{b}}^{†}\hat{b}+\frac{1}{2}\right)\\ & \;+\hbar g_{\text{QED}}\left(\hat{a}+{\hat{a}}^{†}\right)\left(\hat{b}+{\hat{b}}^{†}\right)+\hbar D{\left(\hat{a}+{\hat{a}}^{†}\right)}^{2}, \end{array}$$

as shown in the Supplementary Material. In this Hamiltonian, the creation operator ${\hat{a}}^{†}$ and the annihilation operator $\hat{a}$ act on the cavity mode, while the equivalent operators ${\hat{b}}^{†}$ and $\hat{b}$ are associated to the matter excitation, obeying commutation rules $\left[\hat{a},{\hat{a}}^{†}\right]=\left[\hat{b},{\hat{b}}^{†}\right]=1$ . The first two terms on the right-hand side of Eq. (1) indicate the energy of the uncoupled (or bare) cavity modes and matter excitations at (angular) frequencies *ω*_cav_ and *ω*_mat_, respectively, with *ℏ* the reduced Planck constant. The third term describes the light–matter coupling, which is parameterized by the coupling strength *g*_QED_, and where we include the antiresonant terms $\hat{a}\hat{b}$ and ${\hat{a}}^{†}{\hat{b}}^{†}$ required to describe the ultrastrong coupling regime correctly. *g*_QED_ can in principle depend on *ω*_cav_ and *ω*_mat_ in specific systems (Section S6 in Supplementary Material). Last, we introduce the diamagnetic term, scaled by a parameter *D* that is initially considered to have an arbitrary value (including the zero value). This diamagnetic term, which is included in many (but not all) studies of ultrastrong coupling, is negligible in the strong coupling regime, but becomes important under ultrastrong coupling. It typically originates from the |**A**_⊥_|^2^ term of the minimal coupling Hamiltonian, where **A**_⊥_ is the transverse vector potential. In the main text, we work in the Coulomb gauge, where the vector potential is completely transverse (∇⋅**A** = 0, and thus, **A**_⊥_ = **A**), so that hereafter we omit the symbol ⊥ in **A** for brevity.

From the Hopfield Hamiltonian, we can obtain the equations of motion of the displacements (or oscillation amplitudes) of two quantum oscillators. With this aim, we connect the creation and annihilation operators from the Hamiltonian in Eq. (1) with the quantum operators ${\hat{x}}_{\text{cav}}=\sqrt{\frac{\hbar }{2\omega_{\text{cav}}}}\left(\hat{a}+{\hat{a}}^{†}\right)$ , ${\hat{x}}_{\text{mat}}=\sqrt{\frac{\hbar }{2\omega_{\text{mat}}}}\left(\hat{b}+{\hat{b}}^{†}\right)$ , ${\hat{p}}_{\text{cav}}=-i\sqrt{\frac{\hbar \omega_{\text{cav}}}{2}}\left(\hat{a}-{\hat{a}}^{†}\right)$ , and ${\hat{p}}_{\text{mat}}=-i\sqrt{\frac{\hbar \omega_{\text{mat}}}{2}}\left(\hat{b}-{\hat{b}}^{†}\right)$ . These operators correspond to the canonical position and momentum operators of harmonic oscillators (except that no mass has been included in their definitions). They fulfill the canonical commutation relations $[{\hat{x}}_{\text{mat}},{\hat{x}}_{\text{cav}}]=\left[{\hat{p}}_{\text{mat}},{\hat{p}}_{\text{cav}}\right]=\left[{\hat{x}}_{\text{mat}},{\hat{p}}_{\text{cav}}\right]=\left[{\hat{x}}_{\text{cav}},{\hat{p}}_{\text{mat}}\right]=0$ , and $\left[{\hat{x}}_{\text{mat}},{\hat{p}}_{\text{mat}}\right]=\left[{\hat{x}}_{\text{cav}},{\hat{p}}_{\text{cav}}\right]=i\hbar$ . The dynamics of these operators are calculated from the general Heisenberg equation of motion of an operator $\hat{O}$ , $\frac{d}{dt}\hat{O}=\frac{1}{i\hbar }\left[\hat{O},\hat{H}\right]$ . We convert the four resulting first-order differential equations into two second-order equations by eliminating the momentum operators and obtain the following equations of motion for the expectation values $\left\langle {\hat{x}}_{\text{cav}}\right\rangle$ and $\left\langle {\hat{x}}_{\text{mat}}\right\rangle$ :

(2a) $$\begin{array}{ll} & \left\langle {\ddot{\hat{x}}}_{\text{cav}}\right\rangle +\left(\omega_{\text{cav}}^{2}+4D\omega_{\text{cav}}\right)\left\langle {\hat{x}}_{\text{cav}}\right\rangle +2g_{\text{QED}}\sqrt{\omega_{\text{cav}}\omega_{\text{mat}}}\left\langle {\hat{x}}_{\text{mat}}\right\rangle =0, \end{array}$$

(2b) $$\left\langle {\ddot{\hat{x}}}_{\text{mat}}\right\rangle +\omega_{\text{mat}}^{2}\left\langle {\hat{x}}_{\text{mat}}\right\rangle +2g_{\text{QED}}\sqrt{\omega_{\text{cav}}\omega_{\text{mat}}}\left\langle {\hat{x}}_{\text{cav}}\right\rangle =0.$$

These are not the only classical equations that could describe the spectra of the coupled system. Any Hamiltonian ${\hat{H}}_{2}$ related to the Hopfield Hamiltonian ${\hat{H}}_{1}$ by a unitary transformation will have the same eigenfrequencies but will lead to different Heisenberg equations of motion. We perform a unitary transformation to ${\hat{H}}_{1}$ with the operator $\hat{U}={\text{e}}^{-\text{i}\frac{\pi }{2}{\hat{b}}^{†}\hat{b}}$ . In the new reference frame, ${\hat{H}}_{2}=\hat{U}{\hat{H}}_{1}{\hat{U}}^{†}+i\hbar \frac{\partial \hat{U}}{\partial t}{\hat{U}}^{†}$ is expressed as:

(3) $$\begin{array}{ll} {\hat{H}}_{2} & =\hbar \omega_{\text{cav}}\left({\hat{a}}^{†}\hat{a}+\frac{1}{2}\right)+\hbar \omega_{\text{mat}}\left({\hat{b}}^{†\prime }{\hat{b}}^{\prime }+\frac{1}{2}\right)\\ & \;+i\hbar g_{\text{QED}}\left(\hat{a}+{\hat{a}}^{†}\right)\left({\hat{b}}^{\prime }-{\hat{b}}^{†\prime }\right)+\hbar D{\left(\hat{a}+{\hat{a}}^{†}\right)}^{2}, \end{array}$$

where the prime ′ denotes that the matter operators are transformed ( $\hat{b}\to i{\hat{b}}^{\prime }$ and ${\hat{b}}^{†}\to -i{\hat{b}}^{†\prime }$ ). In the representation of position and momentum operators, this transformation can be understood as a rotation in phase space so that the canonical variables transform as

(4a) $${\hat{x}}_{\text{mat}}\to -\frac{{\hat{p}}_{\text{mat}}^{\prime }}{\omega_{\text{mat}}},$$

(4b) $${\hat{p}}_{\text{mat}}\to \omega_{\text{mat}}{\hat{x}}_{\text{mat}}^{\prime }.$$

In this new reference frame, we can calculate the equations of motion for the expectation values $\left\langle {\hat{x}}_{\text{cav}}\right\rangle$ and $\left\langle {\hat{x}}_{\text{mat}}^{\prime }\right\rangle$ :

(5a) $$\begin{array}{ll} & \;\left\langle {\ddot{\hat{x}}}_{\text{cav}}\right\rangle +\left(\omega_{\text{cav}}^{2}+4D\omega_{\text{cav}}-4g_{\text{QED}}^{2}\frac{\omega_{\text{cav}}}{\omega_{\text{mat}}}\right)\left\langle {\hat{x}}_{\text{cav}}\right\rangle \\ & \;-2g_{\text{QED}}\sqrt{\frac{\omega_{\text{cav}}}{\omega_{\text{mat}}}}\left\langle {\dot{\hat{x}}}_{\text{mat}}^{\prime }\right\rangle =0, \end{array}$$

(5b) $$\left\langle {\ddot{\hat{x}}}_{\text{mat}}^{\prime }\right\rangle +\omega_{\text{mat}}^{2}\left\langle {\hat{x}}_{\text{mat}}^{\prime }\right\rangle +2g_{\text{QED}}\sqrt{\frac{\omega_{\text{cav}}}{\omega_{\text{mat}}}}\left\langle {\dot{\hat{x}}}_{\text{cav}}\right\rangle =0.$$

We find that, in contrast to Eq. (2), the coupling term is now proportional to the time derivative of the oscillation amplitudes.

To obtain the classical harmonic oscillator models, it is just necessary to associate the expectation values of the quantum operators to classical oscillation amplitudes, e.g., $\left\langle {\hat{x}}_{\text{cav}}\right\rangle \to x_{\text{cav}}$ , so that Eq. (2) becomes

(6a) $${\ddot{x}}_{\text{cav}}+\left(\omega_{\text{cav}}^{2}+4D\omega_{\text{cav}}\right)x_{\text{cav}}+2g_{\text{QED}}\sqrt{\omega_{\text{cav}}\omega_{\text{mat}}}x_{\text{mat}}=0,$$

(6b) $${\ddot{x}}_{\text{mat}}+\omega_{\text{mat}}^{2}x_{\text{mat}}+2g_{\text{QED}}\sqrt{\omega_{\text{cav}}\omega_{\text{mat}}}x_{\text{cav}}=0,$$

and Eq. (5) becomes

(7a) $$\begin{array}{ll} & \;{\ddot{x}}_{\text{cav}}+\left(\omega_{\text{cav}}^{2}+4D\omega_{\text{cav}}-4g_{\text{QED}}^{2}\frac{\omega_{\text{cav}}}{\omega_{\text{mat}}}\right)x_{\text{cav}}\\ & \;-2g_{\text{QED}}\sqrt{\frac{\omega_{\text{cav}}}{\omega_{\text{mat}}}}{\dot{x}}_{\text{mat}}=0, \end{array}$$

(7b) $${\ddot{x}}_{\text{mat}}+\omega_{\text{mat}}^{2}x_{\text{mat}}+2g_{\text{QED}}\sqrt{\frac{\omega_{\text{cav}}}{\omega_{\text{mat}}}}{\dot{x}}_{\text{cav}}=0,$$

where we do not make an explicit distinction between $x_{\text{mat}}\equiv \left\langle {\hat{x}}_{\text{mat}}^{\prime }\right\rangle$ used in Eqs. (5) and (7) and $x_{\text{mat}}\equiv \left\langle {\hat{x}}_{\text{mat}}\right\rangle$ in Eqs. (2) and (6). However, the physical interpretation of the expectation values $\left\langle {\hat{x}}_{\text{mat}}\right\rangle$ and $\left\langle {\hat{x}}_{\text{mat}}^{\prime }\right\rangle$ (or the oscillation amplitudes *x*_mat_ in each set of equations) is different, as discussed in more detail in Section 3 when applying each equation to specific coupled systems. Loss mechanisms are not included in these equations (friction terms proportional to the time derivatives ${\dot{x}}_{\text{cav}}$ and ${\dot{x}}_{\text{mat}}$ ), because they were derived from Hermitian cavity-QED Hamiltonians. Neglecting losses is usually an excellent approximation for calculating the eigenfrequencies and eigenvectors of the system in the ultrastrong coupling regime, where the coupling strength can be much larger than the system losses (the inclusion of dissipation in cavity-QED descriptions is discussed in Refs. [49], [50]).

Importantly, once the different interpretation of $\left\langle {\hat{x}}_{\text{mat}}\right\rangle$ and $\left\langle {\hat{x}}_{\text{mat}}^{\prime }\right\rangle$ is accounted for, the two sets of coupled harmonic oscillator equations can be used to obtain the same result for any physical magnitude of a given system, as they correspond to Hamiltonians related by a unitary transformation. In particular, the eigenfrequencies of Eqs. (6) and (7) are identical.

Thus, it is always possible to obtain the optical response of the coupled system by considering the coupling to be proportional to either the oscillation amplitudes or their time derivatives. An important point to notice is that, in both Eqs. (6b) and (7b), the “matter resonant frequency” (square root of the term proportional to *x*_mat_) is the bare frequency *ω*_mat_. However, the “cavity resonant frequency” (square root of the term proportional to *x*_cav_) is different in the different oscillator models. In the model characterized by Eq. (6a), the shifted cavity frequency is $\sqrt{\omega_{\text{cav}}^{2}+4D\omega_{\text{cav}}}$ , while in Eq. (7a), the shifted cavity frequency is $\sqrt{\omega_{\text{cav}}^{2}+4D\omega_{\text{cav}}-4g_{\text{QED}}^{2}\frac{\omega_{\text{cav}}}{\omega_{\text{mat}}}}$ (shifts of the matter excitation are discussed in Section S2 of the Supplementary Material). In the following, we use the term dressed cavity/excitation (or dressed/renormalized frequency) when the shift is not zero, and thus the value of the shifted frequency does not coincide with the original value *ω*_cav_ before coupling (notice that $\sqrt{\omega_{\text{cav}}^{2}+4D\omega_{\text{cav}}}=\omega_{\text{cav}}$ when using Eq. (6a) with *D* = 0 and $\sqrt{\omega_{\text{cav}}^{2}+4D\omega_{\text{cav}}-4g_{\text{QED}}^{2}\frac{\omega_{\text{cav}}}{\omega_{\text{mat}}}}=\omega_{\text{cav}}$ when using Eq. (7a) with $D\omega_{\text{mat}}=g_{\text{QED}}^{2}$ , so that in these two cases we will refer to bare cavity frequencies).

In nanophotonics, coupled harmonic oscillator equations have been widely used to fit data without considering frequency renormalization so we adhere to this procedure, i.e., we consider harmonic oscillator models where the frequency of the cavity and matter excitations are the bare ones. This approach gives preference to the model with coupling constant proportional to the oscillation amplitude (Eq. (6)) or to its derivative (Eq. (7)), depending on the value of *D*, as discussed next. Thus, throughout the remaining of this paper (including the Supplementary Material unless otherwise stated), we analyze these two preferred models using bare frequencies. We denote these models the Spring Coupling (SpC) model and the Momentum Coupling (MoC) model, respectively. Other models are discussed in Section S2 and summarized in Section S4 of the Supplementary Material. Additionally, Section S1 of the Supplementary Material details how to obtain the classical coupled harmonic oscillator equations directly from the classical electromagnetic Lagrangian.

### 2.2 Spring Coupling (SpC) model

We consider first a system without diamagnetic term, *D* = 0. This choice is appropriate, for example, when the interaction between the emitter and cavity excitations is mediated by Coulomb coupling, as discussed in more detail in Section 3.2. Eq. (6) then becomes

(8a) $${\ddot{x}}_{\text{cav}}+\omega_{\text{cav}}^{2}x_{\text{cav}}+2g_{\text{SpC}}\sqrt{\omega_{\text{cav}}\omega_{\text{mat}}}x_{\text{mat}}=0,$$

(8b) $${\ddot{x}}_{\text{mat}}+\omega_{\text{mat}}^{2}x_{\text{mat}}+2g_{\text{SpC}}\sqrt{\omega_{\text{cav}}\omega_{\text{mat}}}x_{\text{cav}}=0,$$

where the coupling is proportional to the classical oscillation amplitudes *x*_cav_ and *x*_mat_ and we have changed the notation *g*_SpC_ = *g*_QED_ (using a different symbol for the coupling strength in the classical and quantum descriptions becomes useful in Section 2.3). The $\sqrt{\omega_{\text{cav}}\omega_{\text{mat}}}$ prefactor appears directly from the Hamiltonian and ensures that *g* have units of frequency. Other choices of prefactor have been used (such as using the arithmetic mean of the bare frequencies instead of the geometric mean [51]), which are equivalent in the strong coupling regime but not in the ultrastrong one. In the frequency (*ω*) domain, these equations are transformed to

(9a) $$-\omega^{2}x_{\text{cav}}+\omega_{\text{cav}}^{2}x_{\text{cav}}+2g_{\text{SpC}}\sqrt{\omega_{\text{cav}}\omega_{\text{mat}}}x_{\text{mat}}=0,$$

(9b) $$-\omega^{2}x_{\text{mat}}+\omega_{\text{mat}}^{2}x_{\text{mat}}+2g_{\text{SpC}}\sqrt{\omega_{\text{cav}}\omega_{\text{mat}}}x_{\text{cav}}=0.$$

**Figure 2:**
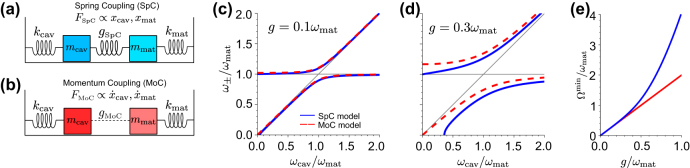
Comparison of the Spring Coupling (SpC) and Momentum Coupling (MoC) models. (a) Schematics of the SpC model in analogy to an oscillator model in classical mechanics. The coupling mechanism of strength *g*_SpC_ is analogous to a force *F*_SpC_ exerted by a spring and proportional to the oscillator displacements *x*_cav_ and *x*_mat_. (b) Schematics of the MoC model. The coupling mechanism of strength *g*_MoC_ is analogous to a force *F*_MoC_ proportional to the time derivatives of the oscillator displacements ${\dot{x}}_{\text{cav}}$ and ${\dot{x}}_{\text{mat}}$ . We represent the coupling with dashed lines to highlight the different coupling mechanism compared with the SpC model, but we are not aware of any system described by the MoC model in classical mechanics. (c) Eigenfrequencies *ω*_±_ of the hybrid states calculated from the bare values *ω*_cav_ and *ω*_mat_, with *ω*_mat_ fixed and *ω*_cav_/*ω*_mat_ changing. *ω*_±_ are obtained from the SpC model (blue solid line, corresponding to Eq. (10)) and the MoC model (red dashed line, Eq. (13)) for coupling strength *g* = *g*_SpC_ = *g*_MoC_ = 0.1 *ω*_mat_. The thin gray lines correspond to the bare cavity frequency *ω*_cav_ and the bare frequency of the matter excitation, *ω*_mat_. (d) Same as panel (c), for coupling strength *g* = *g*_SpC_ = *g*_MoC_ = 0.3 *ω*_mat_. (e) Minimum splitting between the hybrid modes Ω^min^ = *ω*_+_ − *ω*_−_, as a function of the coupling strength *g* for the SpC model (blue solid line) and the MoC model (red solid line). All frequencies in panels (c–e) are normalized with respect to the fixed frequency of the matter excitation *ω*_mat_, so that the results do not depend on the particular value of *ω*_mat_, only on the *ω*_cav_/*ω*_mat_ and *g*/*ω*_mat_ ratios.

We refer to Eqs. (8) and (9) as the Spring Coupling (SpC) model because they are analogous to the equations describing the movement of two coupled springs (sketch in Figure 2(a)) (we emphasize that we could also describe the same physics of ultrastrongly coupled systems by setting *D* = 0 in Eq. (7), but, in this case, the dressed frequency $\sqrt{\omega_{\text{cav}}^{2}-4g_{\text{SpC}}^{2}\frac{\omega_{\text{cav}}}{\omega_{\text{mat}}}}$ would appear in the equations instead of the bare one, contrary to our previous choice). The eigenfrequencies *ω*_±,SpC_ of the SpC model are obtained by diagonalizing the matrix associated with Eq. (9), which leads to

(10) $$\omega_{\pm ,\text{SpC}}=\frac{1}{\sqrt{2}}\sqrt{\omega_{\text{cav}}^{2}+\omega_{\text{mat}}^{2}\pm \sqrt{{\left(\omega_{\text{cav}}^{2}-\omega_{\text{mat}}^{2}\right)}^{2}+16g_{\text{SpC}}^{2}\omega_{\text{cav}}\omega_{\text{mat}}}}.$$

We note that frequencies given by Eq. (10) correspond to the energy difference between the first excited and ground state, and not to the absolute values of the eigenfrequencies themselves. This distinction is not necessary in classical descriptions that set the energy of the ground state to zero (or a fixed value). However, the cavity-QED model indicates a *g*_QED_-dependent shift of the ground-state energy from zero, which is a fully quantum phenomenon. The information of this shift is lost when we take the expectation value of the operators $\left\langle {\hat{x}}_{\text{cav}}\right\rangle$ and $\left\langle {\hat{x}}_{\text{mat}}\right\rangle$ in Eq. (2). The *g*_QED_ dependence of this shift can be found, for instance, in Figure 2(f) of Ref. [21].

### 2.3 Momentum Coupling (MoC) model

For a diamagnetic term with $D=\frac{g_{\text{QED}}^{2}}{\omega_{\text{mat}}}$ (this value normally appears in atomic physics and in cavity-QED models [23] in the Coulomb Gauge and is discussed in Ref. [21] and Section 3.1), Eq. (7) takes the form

(11a) $${\ddot{x}}_{\text{cav}}+\omega_{\text{cav}}^{2}x_{\text{cav}}-2g_{\text{MoC}}{\dot{x}}_{\text{mat}}=0,$$

(11b) $${\ddot{x}}_{\text{mat}}+\omega_{\text{mat}}^{2}x_{\text{mat}}+2g_{\text{MoC}}{\dot{x}}_{\text{cav}}=0,$$

with the coupling term proportional to the time derivative of the oscillation amplitudes (the “velocities”) so that we call this model the Momentum Coupling (MoC) model (sketch in Figure 2(b)). The coupling strength *g*_MoC_ in these equations is related to the constant *g*_QED_ in the cavity-QED Hamiltonian as $g_{\text{MoC}}=\sqrt{\frac{\omega_{\text{cav}}}{\omega_{\text{mat}}}}g_{\text{QED}}$ (and thus $D=\frac{g_{\text{QED}}^{2}}{\omega_{\text{mat}}}=\frac{g_{\text{MoC}}^{2}}{\omega_{\text{cav}}}$ ). We introduce this new coupling strength because, in this way, (i) Eqs. (11a) and (11b) take the same form as in previous work [52], [53], [54] and (ii) *g*_MoC_ becomes independent of the resonant frequencies *ω*_mat_ and *ω*_cav_ for the systems studied in Section 3. However, it is also possible to write Eqs. (11a) and (11b) in terms of *g*_QED_ as long as one is consistent in all the derivation. We further emphasize that *g*_MoC_ = *g*_QED_ in resonance (*ω*_cav_ = *ω*_mat_), and these two parameters only take significantly different values for strong detuning. In the frequency domain, Eq. (11) becomes

(12a) $$-\omega^{2}x_{\text{cav}}+\omega_{\text{cav}}^{2}x_{\text{cav}}+2i\omega g_{\text{MoC}}x_{\text{mat}}=0,$$

(12b) $$-\omega^{2}x_{\text{mat}}+\omega_{\text{mat}}^{2}x_{\text{mat}}-2i\omega g_{\text{MoC}}x_{\text{cav}}=0,$$

and the corresponding eigenfrequencies are

(13) $$\omega_{\pm ,\text{MoC}}=\frac{1}{\sqrt{2}}\sqrt{\omega_{\text{cav}}^{2}+\omega_{\text{mat}}^{2}+4g_{\text{MoC}}^{2}\pm \sqrt{{\left(\omega_{\text{cav}}^{2}+\omega_{\text{mat}}^{2}+4g_{\text{MoC}}^{2}\right)}^{2}-4\omega_{\text{cav}}^{2}\omega_{\text{mat}}^{2}}}.$$

Although the MoC is used to describe the coupling between matter excitations and cavity modes (Figure 2(b)), we are not aware of any equivalent mechanical system in classical mechanics that follows the equations of motion in Eqs. (11a) and (11b) (with coupling terms proportional to the time derivatives of the oscillation amplitude, similarly to friction terms but describing the interaction between two different oscillators). This is in contrast to the SpC model where the equivalent system, composed of masses and springs, is shown in Figure 2(a).

### 2.4 Comparison of the MoC and SpC models

As mentioned above, the MoC and SpC models are appropriate when $D=g_{\text{QED}}^{2}/\omega_{\text{mat}}$ and *D* = 0, respectively (we emphasize again that the resonant frequencies *ω*_cav_, *ω*_mat_ in these models are the bare resonant frequencies). Further, regardless of whether the diamagnetic term should be included in the description or not, these models have been used in the past as phenomenological tools for extracting coupling parameters by fitting the spectra of the coupled system obtained from experimental data or simulations [17], [18], [51], [52], [53], [54], [55], [56], [57], [58], [59]. In this section, we compare the results provided by both models as a function of the coupling strength.

The MoC and SpC models are known to give very different results for *g* ≫ 0.1*ω*_mat_, as we briefly illustrate in this section (we use *g* in this subsection to refer to *g*_SpC_ or *g*_MoC_ in discussions that are valid for both models). Figure 2(c) compares the eigenfrequencies of the SpC (blue solid line) and MoC (red dashed line) models for *g* = 0.1*ω*_mat_, as given by Eqs. (10) and (13), respectively. For simplicity, we consider that the coupling strength is the same for all values of *ω*_cav_ (a different parameter choice is discussed in Section S6 of the Supplementary Material). The eigenfrequencies of the hybrid modes *ω*_±_ are calculated as a function of the bare cavity frequency *ω*_cav_, with the bare *ω*_mat_ frequency fixed (all frequencies are normalized by *ω*_mat_, so that the figures are independent of the value of this parameter). The eigenfrequencies obtained within the MoC and SpC models follow a nearly identical dependence on *ω*_cav_, and the agreement is even better for *g* < 0.1 *ω*_mat_. Thus, when analyzing systems not in the ultrastrong coupling regime, the two models can generally be used interchangeably with minimal impact on the results, although exceptions can exist [60].

In contrast, the choice of the model is crucial for even larger coupling strengths, such that the system is well into the ultrastrong coupling regime. The differences between the two models are illustrated in Figure 2(d) for coupling strength *g* = 0.3 *ω*_mat_. In this case, the two models predict significantly different eigenfrequencies of the coupled system. The difference is smaller for larger cavity frequencies, *ω*_cav_ ≫ *ω*_mat_, because the oscillators become uncoupled and the eigenfrequencies approach the bare frequencies *ω*_cav_ and *ω*_mat_ in the two models. However, even for a relatively large $\frac{\omega_{\text{cav}}}{\omega_{\text{mat}}}=1.5$ , the difference between the values of *ω*_±_ according to the two models is around 10 %.

We compare next the splitting Ω = *ω*_+_ − *ω*_−_ between the two eigenmodes at zero detuning, *ω*_cav_ = *ω*_mat_. In the MoC model, the splitting equals twice the coupling strength, i.e., Ω = 2*g*, which is the minimum splitting in this model [61], [62]. On the other hand, in the SpC model, the relation between Ω and the coupling strength for zero detuning is

(14) $$\begin{array}{ll} \Omega_{\text{SpC}} & =\omega_{+,\text{SpC}}-\omega_{-,\text{SpC}}\\ & =\omega_{\text{mat}}\left(\sqrt{1+\frac{2g_{\text{SpC}}}{\omega_{\text{mat}}}}-\sqrt{1-\frac{2g_{\text{SpC}}}{\omega_{\text{mat}}}}\right). \end{array}$$

We find Ω_SpC_ = 2.11*g*_SpC_ for the values used in Figure 2(d). Further, according to the SpC model, the minimum splitting between the branches does not happen at zero detuning but at cavity frequencies larger than the matter excitation frequencies. To further emphasize the difference between the models, Figure 2(e) shows the minimum splitting as a function of coupling strength, with a linear dependence for the MoC (red solid line) model, Ω^min^ = 2*g*, in contrast with the strong deviation from nonlinearity of the SpC model results (blue line) for *g* ≫ 0.1*ω*_mat_. As a consequence, close to the so-called deep strong coupling regime $\left(\frac{g}{\omega_{\text{mat}}}\approx 1\right)$ , $\Omega_{\text{SpC}}^{\min}$ is approximately twice that of the MoC model.

Last, Figure 2(d) shows important differences at small cavity frequencies, *ω*_cav_ ≪ *ω*_mat_. The dispersion of the MoC model shows two hybrid modes for all values of the detuning, with the lower mode frequency *ω*_−,MoC_ tending toward *ω*_cav_ for decreasing value of *ω*_cav_. In contrast, for the SpC model, the lower mode ceases to exist (*ω*_−,SpC_ becomes imaginary) under the condition $\frac{\omega_{\text{cav}}}{\omega_{\text{mat}}}<{\left(\frac{2g_{\text{SpC}}}{\omega_{\text{mat}}}\right)}^{2}$ (for fixed *g*_SpC_ = 0.3*ω*_mat_; see Section S6 of the Supplementary Material where a different choice is discussed). Further, in the SpC description, the upper branch approaches the bare matter frequency at *ω*_cav_ → 0, but this is not the case in the MoC model, where the corresponding asymptotic limit is $\omega_{+,\text{MoC}}=\sqrt{\omega_{\text{mat}}^{2}+4g_{\text{MoC}}^{2}}$ . Thus, in the MoC model, the coupling affects the upper hybrid mode even in this highly detuned situation.

The two models’ different asymptotic limits of the upper branch determine the predicted range of energies where hybrid modes can exist. The MoC results show a frequency band between *ω*_mat_ and $\sqrt{\omega_{\text{mat}}^{2}+4g_{\text{MoC}}^{2}}$ with no modes available. This forbidden band is not present in the SpC dispersion. In Section 3 and Section S8 of the Supplementary Material, we connect this result with the Reststrahlen band of polar materials and show that we can reproduce the experimental dispersion of these materials by using the MoC [45] and alternative models but not the SpC model.

The connection between classical and quantum models is summarized in Table 1. The classical SpC and MoC models result in the same eigenfrequencies as cavity-QED Hamiltonians without the diamagnetic term (*D* = 0) and with $D=\frac{g_{\text{QED}}^{2}}{\omega_{\text{mat}}}=\frac{g_{\text{MoC}}^{2}}{\omega_{\text{cav}}}$ , respectively. Other classical coupled harmonic oscillator models where dressed frequencies are used instead of the bare ones (with an associated change of the coupling term) are discussed in Section S2 of the SI. For completeness, we also discuss in Section S5 of the Supplementary Material an often-used linearized model that is a good approximation to the MoC and SpC models for low coupling strengths (especially for the anticrossing region of the spectrum corresponding to small detunings). However, this linearized model is not appropriate in the ultrastrong coupling regime.

**Table 1:** Summary of the correspondences of the classical SpC and MoC models with the cavity-QED description without diamagnetic term *D* = 0 (second column) and with diamagnetic term and $D=\frac{g_{\text{MoC}}^{2}}{\omega_{\text{cav}}}$ (third column). The second row shows the two considered cavity-QED Hamiltonians. The third row indicates the equations of motion of the oscillation amplitudes *x*_cav_ and *x*_mat_ obtained with the classical SpC (second column) and MoC (third column) harmonic oscillator models. The fourth row provides the frequencies of the two resulting hybrid modes, which are the same for the cavity-QED and classical models for the value of *D* and choice of coupled harmonic oscillator model indicated in each column. The last row indicates the relationship between the coupling constant *g*_QED_ in the cavity-QED Hamiltonian and those in the classical coupled harmonic oscillator models (*g*_MoC_ and *g*_SpC_). For the system in Section 3.1, *g*_MoC_ is constant and thus $g_{\text{QED}}\propto \sqrt{\omega_{\text{mat}}/\omega_{\text{cav}}}$ .

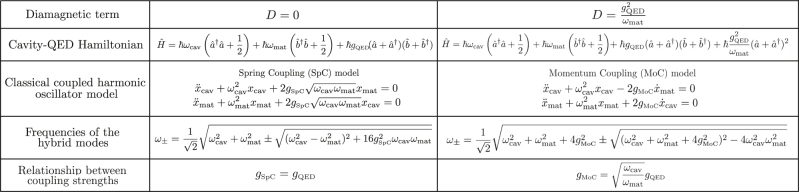

At this point, we have discussed the connections between a general quantum description and classical equations of motion. However, we still need to determine how to choose between the MoC and SpC models for a given system (or equivalently, whether the Hamiltonian has *D* ≠ 0 or *D* = 0). In the next section, we consider three representative systems to explore this question and highlight the key role played by the nature of the matter–cavity interaction (Coulomb coupling or coupling with transverse electromagnetic modes in dielectric cavities).

Additionally, we have focused thus far on the eigenfrequencies, which can be extracted directly from the equations of coupled harmonic oscillators without needing an exact understanding of what the oscillation amplitudes *x*_cav_ and *x*_mat_ represent. However, a clear physical interpretation of these parameters is necessary to evaluate magnitudes of interest, such as the electric field at a given location inside or outside the optical cavity. In Section 3, we also address how *x*_cav_ and *x*_mat_ relate to relevant physical quantities in the representative systems of choice.

## 3 Physical observables from classical models in configurations of interest

We analyze in this section the three canonical nanophotonics systems introduced in Figure 1, for which different cavity-QED Hamiltonians (with and without the diamagnetic term) are appropriate. In Section 3.1, we focus on the textbook case of a single molecular emitter (or another quantum emitter) interacting with transverse electromagnetic modes of the dielectric Fabry–Pérot cavity in Figure 1(a) (in transverse modes, the fields are perpendicular to the wavevector in all Fourier components). As a second example, we analyze in Section 3.2 a molecular emitter close to a small metallic nanoparticle (Figure 1(b)), where the coupling is governed by Coulomb interactions (the fields mediating this interaction are longitudinal, i.e., parallel to the wavevector in all Fourier components). The last example (Section 3.3) consists of an ensemble of molecular emitters (representing a bulk material) inside a Fabry–Pérot cavity (Figure 1(c)), where the molecules couple with a transverse electromagnetic mode of the cavity and also interact with each other through Coulomb coupling.

### 3.1 A quantum emitter interacting with a transverse mode of a dielectric cavity

**Figure 3:**
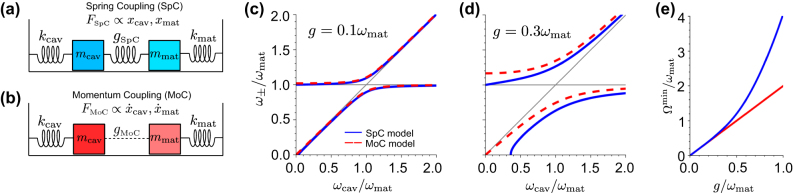
Interaction of a quantum emitter with a transverse cavity mode within the classical MoC model. (a) Schematics of the system. The two oscillators are associated with the vector potential **A** of the cavity mode and the induced dipole moment **d** of the excitation in the quantum emitter, which we consider to be a molecule. The oscillators are coupled with each other with strength *g*_MoC_. The bottom sketch indicates the cavity dimensions that we analyze in the rest of the panels. The emitter is placed at the center of the cavity. The green shaded areas in the sketches represent the field distribution of the cavity mode. (b) Spatial distribution of the electric field for the upper (blue) and the lower (red) hybrid modes at frequencies *ω*_+,MoC_ and *ω*_−,MoC_, respectively, for coupling strength *g*_MoC_ = 2.5 ⋅ 10^−4^ *ω*_cav_. The electric field is calculated along the cavity axis (along the *x* direction in panel (a), with *x* = *y* = *z* = 0 corresponding to the cavity center). The inset is a zoom of the region near the emitter. (c) Contribution to the electric field from the cavity $\Sigma_{\text{cav}}^{\pm }$ (dots) and from the emitter $\Sigma_{\text{mat}}^{\pm }$ (solid lines), for the hybrid mode at frequency *ω*_+,MoC_ (blue) and the hybrid mode at frequency *ω*_−,MoC_ (red), as a function of the detuning *ω*_mat_ − *ω*_cav_. The fields are real and are evaluated at the position (*x*, *y*, *z*) = (10.5 nm, 0, 0), i.e., at 10.5 nm distance from the center of the cavity where the molecular emitter is located (see sketch in (a) for directions), which corresponds to the position indicated by the dashed line in the inset of panel (b). The coupling strength is *g*_MoC_ = 2.5 ⋅ 10^−4^ *ω*_cav_. (d) Same as in (c), for *g*_MoC_ = 0.2 *ω*_cav_.

We consider first a dipole interacting with a single transverse mode of a resonant dielectric cavity (Figures 1(a) and 3(a)). The dipole is associated with matter excitations, and it can represent an excitonic transition of a molecule or quantum dot or a transition between vibrational states, for example. For concreteness, we consider the coupling with a molecular emitter in the following. Cavity-QED models of this system have successfully described phenomena such as the modification of the spontaneous emission rate of the emitter [63], [64], of the photon statistics of the emitted light [60], [65], [66], or of the coherence time of the quantum states [67].

The whole derivation of the equations of motion of the classical variables within the Coulomb gauge is discussed in the Supplementary Material (Section S1), but we summarize it in the following. We represent the molecular emitter as two point charges with relative position **l** (forming a dipole), which couple through Coulomb interactions determined by the potential *V*_Cou_(*l*) approximated as a harmonic one, $V_{\text{Cou}}\left(l\right)=\frac{1}{2}m_{\text{red}}\omega_{\text{mat}}^{2}l^{2}$ , with *l* = |**l**| the distance and *m*_red_ the reduced mass of this two-body system. The dipole moment induced in the molecular emitter is **d** = *q* **l**, where *q* is the absolute value of the charge of the particles in the dipole. On the other hand, the cavity mode is characterized by the vector potential **A**, which is the canonical position variable of the transverse electromagnetic fields [68]. This description does not include nonlinear effects, being thus valid for harmonic molecular vibrations, and also for anharmonic vibrations or excitonic transitions under weak illumination.

In cavity-QED models, the standard approach to describe light–matter interactions in this system is to use the minimal-coupling classical Hamiltonian in the Coulomb gauge of the form $H_{\text{min}-\text{c}}=\frac{q^{2}{\left(p-A\right)}^{2}}{2m_{\text{red}}}$ , where **p** is the classical canonical momentum of the dipolar matter excitation. To obtain the quantum Hamiltonian, we use the following quantization relations [48], [68]:

(15) $$\hat{A}\left(r\right)=\sqrt{\frac{\hbar }{2\omega_{\text{cav}}\varepsilon_{0}V_{\text{eff}}}}\Xi \left(r\right)\left(\hat{a}+{\hat{a}}^{†}\right),$$

(16) $$\hat{\Pi }\left(r\right)=-i\sqrt{\frac{\hbar \omega_{\text{cav}}\varepsilon_{0}V_{\text{eff}}}{2}}\Xi \left(r\right)\left(\hat{a}-{\hat{a}}^{†}\right),$$

(17) $$\hat{d}=\sqrt{\frac{\hbar f_{\text{mat}}}{2\omega_{\text{mat}}}}\left(\hat{b}+{\hat{b}}^{†}\right),$$

(18) $$\hat{p}=-i\sqrt{\frac{\hbar \omega_{\text{mat}}}{2f_{\text{mat}}}}\left(\hat{b}-{\hat{b}}^{†}\right),$$

where $\hat{\Pi }\left(r\right)$ is the canonical momentum associated to the vector potential $\hat{A}\left(r\right)$ (see Sections S1, S2 of the Supplementary Material). The function Ξ(**r**) accounts for the spatial distribution of the vector potential of the cavity mode and is normalized so that its maximum value is 1. Further, we have introduced the effective mode volume of the cavity field [69], *V*_eff_, and the oscillator strength of the dipolar excitation $f_{\text{mat}}=\frac{q^{2}}{m_{\text{red}}}$ . From the minimal-coupling Hamiltonian *H*_min-c_, the light–matter interaction term is $H_{\text{int}}=\frac{-q^{2}p\cdot A}{m_{\text{red}}}$ . Considering that the induced dipole moment and the vector potential form an angle *θ*, and using Eqs. (15) and (18), the interaction term of the quantized Hamiltonian becomes

(19) $${\hat{H}}_{\text{int}}=i\hbar \frac{1}{2}\sqrt{\frac{f_{\text{mat}}}{\varepsilon_{0}V_{\text{eff}}}}\sqrt{\frac{\omega_{\text{mat}}}{\omega_{\text{cav}}}}\Xi \left(r\right)\cos\theta \left(\hat{a}+{\hat{a}}^{†}\right)\left(\hat{b}-{\hat{b}}^{†}\right).$$

Comparing this expression with the third term of the Hopfield Hamiltonian (Eq. (3)), we directly obtain that the coupling strength in the cavity-QED formalism is $g_{\text{QED}}=\frac{1}{2}\sqrt{\frac{f_{\text{mat}}}{\varepsilon_{0}V_{\text{eff}}}}\sqrt{\frac{\omega_{\text{mat}}}{\omega_{\text{cav}}}}\Xi \left(r\right)\cos\theta$ . We consider from now on the maximum coupling strength $g_{\text{QED}}=\frac{1}{2}\sqrt{\frac{f_{\text{mat}}}{\varepsilon_{0}V_{\text{eff}}}}\sqrt{\frac{\omega_{\text{mat}}}{\omega_{\text{cav}}}}$ , which is achieved in the position of the maximum field (Ξ(**r**) = 1) for optimal orientation (*θ* = 0). Further, the **A** ^2^ term in the minimal-coupling Hamiltonian leads to a diamagnetic term (fourth term on the right-hand side of Eq. (3)) with $D=\frac{g_{\text{QED}}^{2}}{\omega_{\text{mat}}}$ . Following the discussion of Section 2, the presence of the diamagnetic term in the cavity-QED Hamiltonian with this exact value of *D* indicates that this system can be described by the classical MoC model in Eq. (11).

Next, we use the connection between the classical and cavity-QED approaches to illustrate the procedure to obtain the value of physical observables from the classical oscillation amplitudes of the cavity *x*_cav_ and of the molecular excitation *x*_mat_. The classical coupling strength *g*_MoC_ is directly obtained from the quantum value as $g_{\text{MoC}}=\sqrt{\frac{\omega_{\text{cav}}}{\omega_{\text{mat}}}}g_{\text{QED}}=\frac{1}{2}\sqrt{\frac{f_{\text{mat}}}{\varepsilon_{0}V_{\text{eff}}}}$ . Further, the quantum position operators ( $\propto \hat{a}+{\hat{a}}^{†}$ and $\propto \hat{b}+{\hat{b}}^{†}$ ) and the classical oscillation amplitudes (*x*_cav_ and *x*_mat_) are related by the standard quantization relationship

(20a) $$\text{Re}\left(x_{\text{cav}}\right)=〈{\hat{x}}_{\text{cav}}〉=\sqrt{\frac{\hbar }{2\omega_{\text{cav}}}}〈\hat{a}+{\hat{a}}^{†}〉,$$

(20b) $$\text{Re}\left(x_{\text{mat}}\right)=〈{\hat{x}}_{\text{mat}}〉=\sqrt{\frac{\hbar }{2\omega_{\text{mat}}}}〈\hat{b}+{\hat{b}}^{†}〉,$$

where, for an appropriate comparison between classical amplitudes and quantum operators, the real part of the oscillator amplitudes must be taken: Re(*x*_cav_) = Re(|*x*_cav_|e^−i*ωt*+*ϕ*^) ∝|*x*_cav_| cos(*ωt* + *ϕ*), with *ϕ* a phase. Equations (20a) and (15) indicate that the oscillation amplitude *x*_cav_ in the MoC model (Eq. (11)) is given by $x_{\text{cav}}=A\sqrt{\varepsilon_{0}V_{\text{eff}}}$ , where A is the maximum amplitude of the classical vector potential (i.e., in the position where Ξ(**r**) = 1). Therefore, the oscillation amplitude *x*_cav_ can be used to calculate the spatial distribution of this potential as $A\left(r\right)=A\Xi \left(r\right)=\frac{x_{\text{cav}}}{\sqrt{\varepsilon_{0}V_{\text{eff}}}}\Xi \left(r\right)$ ( $A\left(r\right)=\left\langle \hat{A}\left(r\right)\right\rangle$ is the classical counterpart of the quantum operator of the vector potential). Equivalently, from Eqs. (20b) and (17), the amplitude of the oscillator corresponding to the matter excitation is directly connected with the induced classical dipole moment (*d* = |**d**|) as $x_{\text{mat}}=\frac{d}{\sqrt{f_{\text{mat}}}}$ . These relations are schematically shown in Figure 3(a).

We are finally in conditions to obtain the value of physical observables such as the electric field from the classical harmonic MoC model. We first consider the spatial distribution of the electric fields of each hybrid mode. The transverse cavity mode field (given by **A**(**r**, *t*)) must be added to the longitudinal near field induced by the induced dipole,1To satisfy the boundary conditions in a closed cavity, additional terms due to image dipoles should be included. However, we neglect these terms for simplicity since their contribution is typically small compared to the near field of the dipole $\propto \frac{1}{r^{3}}$ and of the field of the cavity mode. which is obtained from the scalar Coulomb potential

(21) $$V_{\text{Cou}}\left(r,t\right)=\frac{1}{4\pi \varepsilon_{0}}\frac{d\left(t\right)n_{d}\cdot n_{r}}{|r{|}^{2}},$$

with unit vectors $n_{d}=\frac{d}{\left|d\right|}$ and $n_{r}=\frac{r}{\left|r\right|}$ . The total electric field is, therefore, given as

(22) $$E\left(r,t\right)=-\nabla V_{\text{Cou}}\left(r,t\right)-\frac{\partial A\left(r,t\right)}{\partial t},$$

and the electric field at frequencies *ω*_±,MoC_ of each hybrid mode (given by Eq. (13)) corresponds to

(23) $$\begin{array}{ll} E\left(r,\omega_{\pm ,\text{MoC}}\right) & =\frac{3\left(n_{d}\cdot n_{r}\right)n_{r}-n_{d}}{4\pi \varepsilon_{0}r^{3}}d\left(\omega_{\pm ,\text{MoC}}\right)\\ & \;+i\omega_{\pm ,\text{MoC}}A\left(r,\omega_{\pm ,\text{MoC}}\right)n_{A}\\ & =\underset{E_{\text{mat}}\left(r,\omega_{\pm ,\text{MoC}}\right)}{\underset{︸}{\frac{3\left(n_{d}\cdot n_{r}\right)n_{r}-n_{d}}{4\pi \varepsilon_{0}r^{3}}\sqrt{f_{\text{mat}}}x_{\text{mat}}\left(\omega_{\pm ,\text{MoC}}\right)}}\\ & \;+\underset{E_{\text{cav}}\left(r,\omega_{\pm ,\text{MoC}}\right)}{\underset{︸}{\frac{i\Xi \left(r\right)}{\sqrt{\varepsilon_{0}V_{\text{eff}}}}\omega_{\pm ,\text{MoC}}x_{\text{cav}}\left(\omega_{\pm ,\text{MoC}}\right)n_{A}}}, \end{array}$$

with $n_{A}=\frac{A\left(r\right)}{\left|A\left(r\right)\right|}$ . This equation indicates the contribution of the cavity **E**_cav_(**r**, *ω*_±,MoC_) ∝ *x*_cav_ and of the matter excitation **E**_mat_ (**r**, *ω*_±,MoC_) ∝ *x*_mat_ to the electric field. Further, we use the eigenvectors (Eq. (12a)) to obtain the ratio between the amplitudes *x*_cav_ and *x*_mat_ of the classical harmonic oscillators:

(24) $$\frac{x_{\text{cav}}\left(\omega_{\pm ,\text{MoC}}\right)}{x_{\text{mat}}\left(\omega_{\pm ,\text{MoC}}\right)}=\frac{-2i\omega_{\pm ,\text{MoC}}g_{\text{MoC}}}{\omega_{\text{cav}}^{2}-\omega_{\pm ,\text{MoC}}^{2}}.$$

Inserting Eq. (24) into (23), we obtain the ratio between the contributions of the cavity electric field and the matter excitation.

Equations (23) and (24) are a main result of this subsection and can be used to obtain the electric field at any position and for an arbitrary transverse mode with field distribution given by Ξ(**r**). We consider for illustration the particular case of a molecule (as an example of quantum emitter) introduced in the center of a dielectric cavity consisting in a rectangular vacuum box enclosed in the three dimensions by perfect mirrors, as sketched in Figure 3(a). The cross-section of the box is square, with size *L*_*x*_ = *L*_*y*_ = 292 nm and its height is *L*_*z*_ = 215 nm, which results in a fundamental lowest-order cavity mode at frequency *ω*_cav_ = 3 eV and an effective volume *V*_eff_ = 4.483 ⋅ 10^6^ nm^3^ (for an easier comparison between classical frequencies *ω* and quantum energies *ℏω*, in this paper, we use eV as a unit for both of them). This value of *V*_eff_ is calculated from the general expression of dielectric structures [70]

(25) $$V_{\text{eff}}=\frac{\int \varepsilon \left(r\right)|\Xi \left(r\right){|}^{2}dr}{\max\left[\varepsilon \left(r\right)|\Xi \left(r\right){|}^{2}\right]},$$

where *ɛ*(**r**) refers to the permittivity of the system at position **r**, and in this particular case, we consider *ɛ*(**r**) = 1 inside the cavity. The molecular excitation is nearly resonant with the cavity, *ω*_mat_ ≈ *ω*_cav_ = 3 eV, but its exact frequency is changed to study the effects of detuning. The transition dipole moment $\mu_{\text{mat}}=\sqrt{\frac{\hbar f_{\text{mat}}}{2\omega_{\text{mat}}}}$ (associated with the transition from the ground state to the first excited state) is parallel to the *z* axis and is relatively strong, *μ*_mat_ = 15 Debye, achievable with organic molecules such as nonacene, for example, [71]. This value of the transition dipole moment implies that this molecular emitter has an oscillator strength of $f_{\text{mat}}=\frac{{\left(118.74e\right)}^{2}}{m_{\text{p}}}$ , where *e* is the electron charge and *m*_p_ the mass of the proton. By placing the molecular emitter in the center of the cavity where the electric field of the mode is maximum, this choice of parameters leads to a coupling strength *g*_MoC_ ≈ 2.5 ⋅ 10^−4^ *ω*_cav_, far from the ultrastrong coupling regime (a larger value of *g*_MoC_ is considered at the end of this subsection).

We show in Figure 3(b) the distribution of the *z* component of the electric field inside this cavity for the upper hybrid mode *E*_*z*_(*x*, *ω*_+,MoC_) and for the lower hybrid mode *E*_*z*_(*x*, *ω*_−,MoC_), as obtained from Eq. (23). We plot the fields as a function of the position in the *x* direction with respect to the location of the molecular emitter at the center of the cavity. To highlight the differences between the contributions of the cavity and the induced dipole in the two modes, we choose a slight detuning of *ω*_cav_ − *ω*_mat_ = 1.5 meV. Since the classical MoC model does not give the absolute value of the eigenmode fields, we choose arbitrary units so that the contribution of the cavity mode to the electric field of the upper hybrid mode (**E**_cav_(**r**, *ω*_+,MoC_) in Eq. (23)) has a maximum absolute value of 1. This choice fixes all the other values according to Eq. (24).2The eigenstates of the Hopfield Hamiltonian from Eq. (3) have a symmetry where the cavity contribution of one hybrid mode is the same as the matter contribution of the other mode and vice versa, satisfying the equality $\left\langle \hat{a}+{\hat{a}}^{†}\right\rangle \left(\omega_{\pm ,\text{MoC}}\right)=\left\langle \hat{b}+{\hat{b}}^{†}\right\rangle \left(\omega_{\mp ,\text{MoC}}\right)$ . This property allows us to connect the amplitudes of the classical oscillators of the two hybrid eigenmodes as $\sqrt{\omega_{\text{cav}}}x_{\text{cav}}\left(\omega_{\pm ,\text{MoC}}\right)=\sqrt{\omega_{\text{mat}}}x_{\text{mat}}\left(\omega_{\mp ,\text{MoC}}\right)$ (from Eq. (20)). The fields are dominated by the cavity mode far from the cavity center and by the contribution from the molecular dipole close to *x* = 0. The field distribution shows a clear difference in the behavior of the two hybrid modes. For the upper mode, the induced dipole points in the same direction as the cavity field $\left(\frac{x_{\text{cav}}\left(\omega_{+,\text{MoC}}\right)}{x_{\text{mat}}\left(\omega_{+,\text{MoC}}\right)}>0\right)$ , but in the inverse direction for the lower mode $\left(\frac{x_{\text{cav}}\left(\omega_{-,\text{MoC}}\right)}{x_{\text{mat}}\left(\omega_{-,\text{MoC}}\right)}<0\right)$ . Further, at the detuning considered, the relative contribution of the cavity to the fields is larger for the upper than for the lower mode, as indicated by the values of the electric field far from the molecular emitter at *ω*_+,MoC_ and *ω*_−,MoC_. In contrast, as shown in the inset, the relative contribution from the molecular dipole to the field close to the molecule (*x* = 0) is stronger for the lower mode. Figure 3(b) thus confirms that the classical harmonic oscillator model allows for calculating the relative contribution of cavity and matter for each mode, as desired.

Further, Eqs. (23) and (24) also enable to examine the dependence of the field **E**(**r**, *ω*_±,MoC_) inside the cavity with detuning *ω*_mat_ − *ω*_cav_. Figure 3(c) shows the contributions to this electric field of the cavity and the molecular emitter for each hybrid mode, normalized with respect to the sum of both contributions, according to $\Sigma_{\text{cav}}^{\pm }=\frac{|E_{\text{cav}}\left(\omega_{\pm ,\text{MoC}}\right){|}^{2}}{|E_{\text{cav}}\left(\omega_{\pm ,\text{MoC}}\right){|}^{2}+|E_{\text{mat}}\left(\omega_{\pm ,\text{MoC}}\right){|}^{2}}$ (dots) and $\Sigma_{\text{mat}}^{\pm }=\frac{|E_{\text{mat}}\left(\omega_{\pm ,\text{MoC}}\right){|}^{2}}{|E_{\text{cav}}\left(\omega_{\pm ,\text{MoC}}\right){|}^{2}+|E_{\text{mat}}\left(\omega_{\pm ,\text{MoC}}\right){|}^{2}}$ (solid lines). These ratios play a similar role as the Hopfield coefficients from cavity-QED descriptions. The blue (red) dots and solid lines correspond to the upper (lower) hybrid mode. We obtain **E**_cav_(*ω*_±,MoC_) and **E**_mat_(*ω*_±,MoC_) by replacing Eq. (24) into (23), for a fixed coupling strength *g*_MoC_ = 2.5 ⋅ 10^−4^ *ω*_cav_ and for a distance of 10.5 nm from the molecular emitter in the *x* direction. This position (indicated by the dashed line in the inset of Figure 3(b)) is chosen because it is where the matter and cavity contributions have the same weight for the two hybrid modes at zero detuning and very small coupling strengths ( $\Sigma_{\text{cav}}^{\pm }=\Sigma_{\text{mat}}^{\pm }=0.5$ ). For *ω*_cav_ > *ω*_mat_, the field of the lower mode is predominantly given by the matter excitation ( $\Sigma_{\text{mat}}^{-}>\Sigma_{\text{cav}}^{-}$ as indicated by the red dots and the red solid line). In contrast, for the upper mode, the cavity contribution dominates ( $\Sigma_{\text{cav}}^{+}>\Sigma_{\text{mat}}^{+}$ , blue). Further, already at detunings as small as *ω*_cav_ − *ω*_mat_ ≳ 15meV = 5 ⋅ 10^−3^ *ω*_cav_, the modes are essentially uncoupled for this small coupling strength ( $\Sigma_{\text{mat}}^{+}\ll \Sigma_{\text{cav}}^{+}$ and $\Sigma_{\text{mat}}^{-}\gg \Sigma_{\text{cav}}^{-}$ ).

The coupling strength we have considered in this subsection corresponds to the strong coupling regime (we have neglected losses) but is far from the ultrastrong coupling regime so that the phenomena studied can also be explained with the classical linearized model (Section S5 of the Supplementary Material). On the other hand, we consider again in Figure 3(d) the contributions to the electric field $\Sigma_{\text{cav}}^{\pm }$ and $\Sigma_{\text{mat}}^{\pm }$ as a function of the detuning, but in this case for a considerably larger coupling strength *g*_MoC_ = 0.2*ω*_cav_. This value of *g*_MoC_ is not currently achievable with dielectric cavities at the single molecule or single emitter level (it would correspond to a transition dipole moment *μ*_mat_ = 1.2 ⋅ 10^4^ Debye), but we choose it to illustrate the analysis of ultrastrongly coupled systems within the classical MoC model. Further, such large *g*_MoC_ can be achieved in dielectric cavities fully filled by a material or many molecular emitters, as discussed in Section 3.3. For zero detuning *ω*_cav_ = *ω*_mat_, the contributions of the induced dipole and the cavity are no longer identical in the ultrastrong coupling regime, with $\Sigma_{\text{cav}}^{+}\approx 0.6$ and $\Sigma_{\text{mat}}^{+}\approx 0.4$ for the upper hybrid mode at frequency *ω*_+,MoC_ (and the opposite for the lower hybrid mode). More strikingly, the results in Figure 3(d) indicate a very different tendency of the modes at large detunings as compared to strong coupling, especially in the case of the upper hybrid mode at frequency *ω*_+,MoC_. In the ultrastrong coupling regime, in the *ω*_mat_ → 0 limit (*ω*_mat_ − *ω*_cav_ → −3 eV), this mode (blue solid line and dots) has significant contributions from both the cavity and the matter ( $\Sigma_{\text{cav}}^{+}\approx 0.9$ and $\Sigma_{\text{mat}}^{+}\approx 0.1$ ). Thus, these two excitations do not decouple in this limit. This behavior is consistent with the discussion of the dispersion in Figure 2(d), where at large detunings, the upper mode frequency does not reach the bare frequency *ω*_mat_. The SpC model (not shown) does not reproduce this behavior because the modes become uncoupled ( $\Sigma_{\text{cav}}^{+}\approx 1$ and $\Sigma_{\text{mat}}^{+}\approx 0$ ).

The described methodology thus enables obtaining results equivalent to those of the cavity-QED description (Hopfield Hamiltonian with the diamagnetic term) by using an intuitive classical model of coupled harmonic oscillators. In summary, we have shown in this section how to use the classical MoC model to characterize the fields in a hybrid system composed of a molecular emitter coupled to a transverse mode of a cavity.

### 3.2 A quantum emitter interacting with the longitudinal field of a metallic nanoparticle through Coulomb coupling

Next, we consider a quantum emitter placed close to a metallic nanoparticle to analyze how to model an alternative system and obtain physical observables in the strong and ultrastrong coupling regimes. These nanoparticles are attractive in nanophotonics because they support localized surface plasmon modes characterized by very low effective volumes [18], [71], [72], [73], [74]. Since the coupling strength is inversely proportional to the square root of the effective mode volume, very large coupling strengths can be obtained even when the nanoparticle interacts with a single molecule or quantum dot. We consider again a molecule as a representative quantum emitter.

In order to analyze the interaction of the dipolar plasmonic mode of the nanoparticle with a molecular (harmonic) excitation of dipole moment *d*_mat_, we consider that the size of the nanoparticle and the molecule-nanoparticle distance are much smaller than the light wavelength and treat the system within the quasistatic approximation. Under this approximation, the temporal variation of the vector potential **A** in Eq. (22) is negligible. Therefore, the coupling between the nanoparticle and the molecular emitter is governed by Coulomb interactions expressed by a scalar potential *V*_Cou_. The coupling is then mediated by longitudinal fields, in contrast to the coupling with transverse fields in Section 3.1.

In this context, the emitter-nanoparticle coupling cannot be modeled with the minimal coupling Hamiltonian as in Section 3.1, and it is described instead through the interaction Hamiltonian [75]

(26) $${\hat{H}}_{\text{int}2}=-{\hat{d}}_{\text{mat}}\cdot {\hat{E}}_{\text{cav}}^{\|}\left(r_{\text{mat}}\right).$$

${\hat{E}}_{\text{cav}}^{\|}$ is the electric field associated with the dipolar mode of the nanocavity, which in the quasistatic approximation is completely longitudinal (we indicate this explicitly with the symbol ‖) and *d*_cav_ is the induced plasmonic dipole moment (operator ${\hat{d}}_{\text{cav}}$ ). For simplicity, we consider small spherical particles of radius *R*_cav_ composed by a Drude metal with plasma frequency *ω*_p_, but this approach could be generalized to other systems. The spherical particles present a dipolar plasmonic resonance of Lorentzian lineshape at frequency $\omega_{\text{cav}}=\frac{\omega_{\text{p}}}{\sqrt{3}}$ , and oscillator strength $f_{\text{cav}}=4\pi \varepsilon_{0}R_{\text{cav}}^{3}\omega_{\text{cav}}^{2}$ [76]. The quasistatic field outside them is directly determined by *d*_cav_ according to ${\hat{E}}_{\text{cav}}^{\|}\left(r\right)=\frac{3\left({\hat{d}}_{\text{cav}}\cdot n_{r\text{cav}}\right)n_{r\text{cav}}-{\hat{d}}_{\text{cav}}}{4\pi \varepsilon_{0}|r_{\text{cav}}-r{|}^{3}}$ , where **r**_cav_ is the center of the nanoparticle, |**r** − **r**_cav_| > *R*_cav_, and we define the unit vector $n_{r\text{cav}}=\frac{r-r_{\text{cav}}}{\left|r-r_{\text{cav}}\right|}$ .

We insert the quantized expressions of the induced dipole moments ${\hat{d}}_{\text{cav}}$ and ${\hat{d}}_{\text{mat}}$ of Eq. (17) into the Hamiltonian in Eq. (26) and the expression of ${\hat{E}}_{\text{cav}}^{\|}\left(r\right)$ and obtain

(27) $${\hat{H}}_{\text{int}2}=\hbar g_{\text{SpC}}\left(\hat{a}+{\hat{a}}^{†}\right)\left(\hat{b}+{\hat{b}}^{†}\right),$$

with coupling strength

(28) $$\begin{array}{ll} g_{\text{SpC}} & =\frac{1}{2}\frac{\sqrt{f_{\text{cav}}}\sqrt{f_{\text{mat}}}}{4\pi \varepsilon_{0}|r_{\text{cav}}-r_{\text{mat}}{|}^{3}\sqrt{\omega_{\text{cav}}\omega_{\text{mat}}}}\\ & \;\times \left[n_{d\text{cav}}\cdot n_{d\text{mat}}-3\left(n_{d\text{cav}}\cdot n_{r\text{rel}}\right)\left(n_{d\text{mat}}\cdot n_{r\text{rel}}\right)\right], \end{array}$$

where we have defined the unit vectors as $n_{d\text{cav}}=\frac{d_{\text{cav}}}{\left|d_{\text{cav}}\right|}$ , $n_{d\text{mat}}=\frac{d_{\text{mat}}}{\left|d_{\text{mat}}\right|}$ , and $n_{r\text{rel}}=\frac{r_{\text{cav}}-r_{\text{mat}}}{\left|r_{\text{cav}}-r_{\text{mat}}\right|}$ . The total Hamiltonian is thus the sum of ${\hat{H}}_{\text{int}2}$ and the terms related to the energy of the uncoupled plasmon and molecular excitation, corresponding to the Hopfield Hamiltonian of Eq. (1) without the diamagnetic term (*D* = 0). Thus, the corresponding classical model to be adopted is the SpC model in Section 2.2, with the equations of motion in Eq. (8). Additional details can be found in Section S1 of the Supplementary Material.

**Figure 4:**
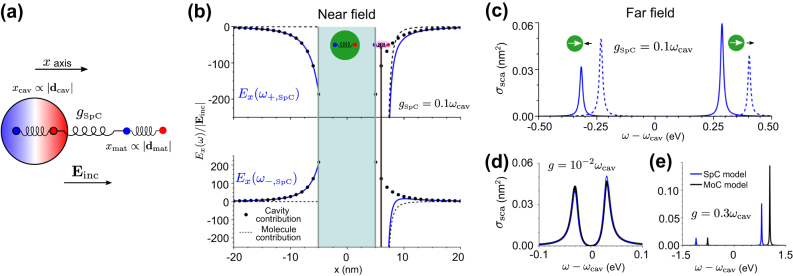
Modeling of the coupling between a quantum emitter and a metallic spherical nanoparticle (a plasmonic nanocavity) within the classical SpC model. (a) Schematics of the system. The quantum emitter is considered to be a molecule. The molecular excitation (of induced dipole moment **d**_mat_) and the dipolar mode of the plasmonic nanocavity (of induced dipole moment **d**_cav_) are described as two harmonic oscillators (of oscillation amplitudes *x*_mat_ and *x*_cav_) that are coupled with strength *g*_SpC_. The system is excited by a laser of electric field amplitude **E**_inc_. The radius of the spherical nanoparticle is 5 nm, and the molecular emitter is placed at a 1 nm distance from the nanoparticle surface along the *x* axis (the center of the nanoparticle corresponds to *x* = *y* = *z* = 0). **d**_cav_, **d**_mat_, and **E**_inc_ are polarized along *x*. (b) Electric field distribution along the *x* axis (*y* = *z* = 0) when the system is excited at the frequency of the upper hybrid mode *ω*_+,SpC_ (top panel) and of the lower hybrid mode *ω*_−,SpC_ (bottom panel). The fields are real and are evaluated only outside the nanocavity, with the positions inside highlighted by the green-shaded area. The position of the molecular emitter is indicated by the vertical brown line. We evaluate the fields for coupling strength *g*_SpC_ = 0.1 *ω*_cav_, and *ω*_cav_ = *ω*_mat_ = 3 eV. For each hybrid mode, the cavity contribution to the field is indicated by dots, the contribution from the emitter by dashed lines, and the total field by blue solid lines. (c) Scattering cross section of the same system, with *g*_SpC_ = 0.1 *ω*_cav_, as a function of the detuning of the laser *ω* − *ω*_cav_. Solid lines: tuned system with frequencies *ω*_cav_ = *ω*_mat_ = 3 eV. Dashed lines: detuned system with frequencies *ω*_cav_ = 3 eV and *ω*_mat_ = 3.2 eV. (d) Scattering cross section of the tuned system (*ω*_cav_ = *ω*_mat_ = 3 eV), comparing the result of the SpC model (blue line) to the results of the MoC model (black line), in the strong coupling regime, *g* = 10^−2^ *ω*_cav_. (e) Same as in (d) for the ultrastrong coupling regime, *g* = 0.3 *ω*_cav_. In all results *f*_cav_ = (4345*e*)^2^/*m*_p_ (where *m*_p_ is the mass of the proton), $F_{\text{cav}}=\sqrt{f_{\text{cav}}}|E_{\text{inc}}|$ , *f*_mat_ = (118.74*e*)^2^/*m*_p_, $F_{\text{mat}}=\sqrt{f_{\text{mat}}}|E_{\text{inc}}|$ , *κ* = 20 meV and *γ* = 10 meV (except that we modify *f*_cav_ in panels (d) and (e) to achieve the desired values of *g*_SpC_).

The representation of the plasmon-molecule system with the SpC model is schematically shown in Figure 4(a). To obtain the observables in this system, we use the equivalence of the oscillation amplitudes *x*_cav_ and *x*_mat_ with the induced dipole moments of the cavity and the molecular (or matter) excitation. This equivalence can be obtained from Eqs. (17) and (20), and it follows $x_{\text{cav}}=\frac{d_{\text{cav}}}{\sqrt{f_{\text{cav}}}}$ and $x_{\text{mat}}=\frac{d_{\text{mat}}}{\sqrt{f_{\text{mat}}}}$ . Further, this treatment can be extended to other dipole–dipole interactions beyond the coupling of a molecular emitter with a plasmon (direct dipole–dipole interactions between molecules are considered in Section 3.3).

We consider next that the dipolar mode of the metallic nanoparticle is illuminated by an external field of amplitude **E**_inc_ and frequency *ω*. We introduce this field in the SpC model as a forcing term that acts both onto the nanoparticle and onto the molecular emitter. Specifically, this is done by adding terms $F_{\alpha }{\text{e}}^{-\text{i}\omega t}=\sqrt{f_{\alpha }}|E_{\text{inc}}|{\text{e}}^{-\text{i}\omega t}$ (*α* = “cav” or *α* = “mat”) on the right-hand side of Eq. (8), i.e., the amplitude *F*_*α*_ of the time-dependent force is proportional to the induced dipole moments *d*_*α*_ and the electric field of the illumination (see Section S1 in the Supplementary Material for further details). By solving the equations of motion of the SpC model (Eq. (8)) in the frequency domain with this external force included, we can calculate the induced dipole moments of the cavity plasmon and matter excitation:

(29a) $$\begin{array}{ll} d_{\text{cav}}\left(\omega \right) & =\sqrt{f_{\text{cav}}}x_{\text{cav}}\left(\omega \right)=\sqrt{f_{\text{cav}}}\\ & \;\times \frac{F_{\text{cav}}\left(\omega_{\text{mat}}^{2}-\omega^{2}\right)-F_{\text{mat}}2g_{\text{SpC}}\sqrt{\omega_{\text{cav}}\omega_{\text{mat}}}}{\left(\omega_{\text{cav}}^{2}-\omega^{2}\right)\left(\omega_{\text{mat}}^{2}-\omega^{2}\right)-4g_{\text{SpC}}^{2}\omega_{\text{cav}}\omega_{\text{mat}}}, \end{array}$$

(29b) $$\begin{array}{ll} d_{\text{mat}}\left(\omega \right) & =\sqrt{f_{\text{mat}}}x_{\text{mat}}\left(\omega \right)=\sqrt{f_{\text{mat}}}\\ & \;\times \frac{F_{\text{mat}}\left(\omega_{\text{cav}}^{2}-\omega^{2}\right)-F_{\text{cav}}2g_{\text{SpC}}\sqrt{\omega_{\text{cav}}\omega_{\text{mat}}}}{\left(\omega_{\text{cav}}^{2}-\omega^{2}\right)\left(\omega_{\text{mat}}^{2}-\omega^{2}\right)-4g_{\text{SpC}}^{2}\omega_{\text{cav}}\omega_{\text{mat}}}. \end{array}$$

These expressions are consistent with an alternative classical model that describes the nanocavity and the molecular emitter as dipolar polarizable objects (Section S1 of the Supplementary Material), supporting the validity of the general approach presented here. In the absence of losses [49], the induced dipole moments *d*_cav_ and *d*_mat_ diverge at the eigenfrequencies *ω*_±,SpC_ of the SpC model (Eq. (10)). To avoid these divergences, we add an imaginary part to the bare cavity and matter frequencies in this section. These imaginary parts are related to the decay rates of the cavity, *κ*, and of the matter excitation, *γ*, as $Im\left(\omega_{\text{cav}}\right)=-\frac{\kappa }{2}$ and $Im\left(\omega_{\text{mat}}\right)=-\frac{\gamma }{2}$ , respectively.

As an example, we consider a metallic spherical nanoparticle of radius *R*_cav_ = 5 nm and with a cavity mode of frequency *ω*_cav_ = 3 eV. We consider the same molecular emitter of Section 3.1, with a strong transition dipole moment of magnitude *μ*_mat_ = 15 Debye. As indicated by Eq. (28), the coupling strength of the system can be adjusted based on the position and orientation of the molecular emitter. We choose that the dipolar molecular transition is polarized perpendicularly to the surface of the nanoparticle and parallel to the amplitude of the incident field **E**_inc_, to maximize the coupling strength (as a consequence **d**_cav_, **d**_mat_, ${\hat{E}}_{\text{cav}}^{\|}\left(r_{\text{mat}}\right)$ , and **n**_**r**rel_ are all oriented in the same direction in, e.g., Eqs. (26) and (28)). With this choice, and placing the molecular emitter at 1 nm from the surface of the nanoparticle, we obtain a coupling strength *g*_SpC_ ≈ 300 meV = 0.1 *ω*_cav_ and thus reach the limit of the ultrastrong coupling regime. This large value of *g*_SpC_ is possible due to the small size of the nanoparticle (large field confinement) and to the strong transition dipole moment considered for the molecular emitter, which lies slightly beyond the values of *μ*_mat_ = 3 − 5 Debyes corresponding to typical molecules used in combination with plasmonic systems. Even larger field confinement may be possible in nonspherical experimental nanostructure configurations that exploit very narrow gaps [18], [77]. To ensure that the system is also in the strong coupling regime when considering lower values of *g*_SpC_ below, we choose *γ* = 10 meV and a damping rate of the plasmonic cavity *κ* = 20 meV that is small compared to those of usual plasmonic metals.

The induced dipole moments obtained from Eq. (29) can be used, for example, to calculate the near-field distribution under excitation at frequency *ω*. The total electric field is the sum of the cavity $E_{\text{cav}}^{\|}$ and molecular or matter contribution $E_{\text{mat}}^{\|}$ . Under the quasistatic approximation, with $d_{\text{cav}}\left(\omega \right)=\sqrt{f_{\text{cav}}}x_{\text{cav}}\left(\omega \right)$ and $d_{\text{mat}}\left(\omega \right)=\sqrt{f_{\text{mat}}}x_{\text{mat}}\left(\omega \right)$ , we obtain that the fields at position **r** outside the metallic nanoparticle, |**r** − **r**_cav_| > *R*_cav_, depend on the amplitude of the harmonic oscillators as

(30) $$\begin{array}{ll} E^{\|}\left(r,\omega \right) & =\underset{E_{\text{cav}}^{\|}\left(r,\omega \right)}{\underset{︸}{\frac{3\left(n_{d\text{cav}}\cdot n_{r\text{cav}}\right)n_{r\text{cav}}-n_{d\text{cav}}}{4\pi \varepsilon_{0}|r-r_{\text{cav}}{|}^{3}}\sqrt{f_{\text{cav}}}x_{\text{cav}}\left(\omega \right)}}\\ & \;+\underset{E_{\text{mat}}^{\|}\left(r,\omega \right)}{\underset{︸}{\frac{3\left(n_{d\text{mat}}\cdot n_{r\text{mat}}\right)n_{r\text{mat}}-n_{d\text{mat}}}{4\pi \varepsilon_{0}|r-r_{\text{mat}}{|}^{3}}\sqrt{f_{\text{mat}}}x_{\text{mat}}\left(\omega \right)}}. \end{array}$$

From this expression, the fields at the frequency of each hybrid mode are calculated by replacing into Eq. (30) the oscillation amplitudes in Eq. (29) induced at the mode frequencies *ω*_±,SpC_.

The electric fields along the *x*-axis associated with the upper and lower mode frequencies are plotted in the top and bottom panels of Figure 4(b) (blue lines), respectively. These fields are real and polarized along the *x* direction. We further show the decomposition of the fields into the contribution of the cavity (black dots) and the molecular emitter (black dashed line) as given by the first and second terms on the right-hand side of Eq. (30), respectively. It can be appreciated from Figure 4(b) that, for example, when the upper hybrid mode is excited, the dipoles associated with the cavity and the molecular emitter are oriented in the same direction (same sign). In contrast, for the lower mode, the dipoles point toward the opposite direction.

The near field plotted in Figure 4(b) is useful for analyzing the behavior of the hybrid modes. Still, it is difficult to measure, and most experiments focus on far-field spectral information, such as in the scattering cross section spectral *σ*_sca_. Due to the small emitter-nanocavity distance, we neglect retardation effects so that *σ*_sca_ is related to the total induced dipole moment of the system as [78]

(31) $$\begin{array}{ll} \sigma_{\text{sca}}\left(\omega \right) & =\frac{\omega^{4}}{6\pi \varepsilon_{0}^{2}c^{4}}{\left|\frac{d_{\text{cav}}\left(\omega \right)}{\left|E_{\text{inc}}\right|}+\frac{d_{\text{mat}}\left(\omega \right)}{\left|E_{\text{inc}}\right|}\right|}^{2}\\ & =\frac{\omega^{4}}{6\pi \varepsilon_{0}^{2}c^{4}}\left|\frac{\sqrt{f_{\text{cav}}}x_{\text{cav}}\left(\omega \right)}{\left|E_{\text{inc}}\right|}n_{d\text{cav}}\right.\\ & \;{\left.+\frac{\sqrt{f_{\text{mat}}}x_{\text{mat}}\left(\omega \right)}{\left|E_{\text{inc}}\right|}n_{d\text{mat}}\right|}^{2}. \end{array}$$

We show in Figure 4(c) the scattering cross section for the same nanoparticle-molecular emitter system in the outset of the ultrastrong coupling regime (*g*_SpC_ = 0.1 *ω*_cav_). Since the oscillator strength of the cavity is much larger than that of the emitter (*f*_cav_ ≫ *f*_mat_), the spectrum is entirely dominated by the cavity contribution, obtained from Eq. (29a) (however, in other systems, where both oscillator strengths are similar, *f*_cav_ ≈ *f*_mat_, it is crucial to consider both contributions in Eq. (31)). The scattering cross section spectra are shown for two different detunings between the nanocavity and the molecular emitter. At zero detuning (*ω*_cav_ = *ω*_mat_ = 3 eV, solid lines in Figure 4(c)) the upper hybrid mode has a (moderately) larger cross section than the lower hybrid mode, mostly due to the *ω* ^4^ factor in Eq. (31). However, when the molecular excitation is blue detuned with respect to the cavity (*ω*_cav_ = 3 eV and *ω*_mat_ = 3.2 eV, dashed line), the strength of the peak in the cross section spectra associated with the lower hybrid mode increases and the upper hybrid mode becomes weaker. This behavior occurs because, for this detuning, the lower hybrid mode acquires a larger contribution of the cavity resonance that dominates the scattering spectra, while the predominantly emitter-like behavior of the upper mode results in a smaller cross section due to *f*_mat_ ≪ *f*_cav_.

To assess the importance of using the classical SpC model to describe this system, we compare the results of the scattering cross section spectra calculated with this model with those obtained using the MoC model. For this purpose, it is necessary to obtain the expressions of the scattering cross section for the latter model under external illumination. By introducing forcing terms in the equations of motion of the MoC model (Eq. (11)) to account for the external field, we obtain the corresponding oscillation amplitudes

(32a) $$x_{\text{cav},MoC}\left(\omega \right)=\frac{F_{\text{cav}}\left(\omega_{\text{mat}}^{2}-\omega^{2}\right)-F_{\text{mat}}2ig_{\text{MoC}}\omega }{\left(\omega_{\text{cav}}^{2}-\omega^{2}\right)\left(\omega_{\text{mat}}^{2}-\omega^{2}\right)-4g_{\text{MoC}}^{2}\omega^{2}},$$

(32b) $$x_{\text{mat},MoC}\left(\omega \right)=\frac{F_{\text{cav}}2ig_{\text{MoC}}\omega +F_{\text{mat}}\left(\omega_{\text{mat}}^{2}-\omega^{2}\right)}{\left(\omega_{\text{cav}}^{2}-\omega^{2}\right)\left(\omega_{\text{mat}}^{2}-\omega^{2}\right)-4g_{\text{MoC}}^{2}\omega^{2}}.$$

We calculate the scattering cross section according to each classical model by introducing these oscillations amplitudes in Eq. (31). Figure 4(d) shows the spectra for the system at zero detuning (*ω*_cav_ = *ω*_mat_ = 3 eV) in the strong coupling regime but far from the ultrastrong coupling regime, with *g* = 10^−2^ *ω*_cav_. As expected, the spectra calculated from the two models overlap almost perfectly (black line: MoC model; blue line: SpC model). The difference between the two calculations is less than 10 % at the hybrid mode frequencies *ω*_±_. This small error is consistent with the good agreement of the eigenfrequencies in Section 2 for this relatively low value of *g*.

In contrast, if the system is well into the ultrastrong coupling regime, with coupling strength *g* = 0.3 *ω*_cav_, the spectra obtained with the two models are very different (Figure 4(e)). There is a clear disagreement in the peak positions due to the difference in the eigenfrequencies of the two models (see Figure 2(d)). Further, the MoC model predicts that the strength of the peak corresponding to the excitation of the upper hybrid mode is twice larger than the equivalent value from the SpC model. These significant differences emphasize the importance of the choice of the model in this regime. However, we note that for such large coupling, higher-order modes of the nanocavity are likely to play an important role in the coupling, which would need to be considered in realistic systems [79]. Further, examining how this analysis is modified when going beyond the quasistatic description would be of interest.

### 3.3 An ensemble of interacting molecules in a Fabry–Pérot cavity

**Figure 5:**
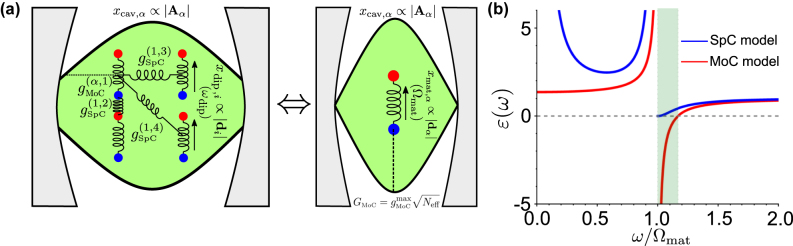
Interaction between matter excitations within a homogeneous material and the transverse modes of a dielectric cavity. (a) (Left) Schematic of the system. The homogeneous material is modeled as an ensemble of dipolar molecular emitters with a vibration at frequency *ω*_dip,*i*_. The oscillators *x*_cav,*α*_ represent the vector potential **A**_*α*_ associated with all modes *α* in the cavity, and the individual matter oscillators *x*_dip,*i*_ represent the induced dipole moments **d**_*i*_ of each molecular emitter. The cavity–molecular emitter interactions are modeled with the MoC model and coupling strength $g_{\text{MoC}}^{\left(\alpha ,i\right)}$ , and the molecule–molecule dipolar interactions with the SpC model and coupling strength $g_{\text{SpC}}^{\left(i,j\right)}$ . We indicate all the interactions of the molecular emitter with index *i* = 1. (Right) Schematic indicating that the description of the full system is equivalent to the coupling, within the MoC model, of the cavity mode *α* with a single molecular excitation of induced dipole moment **d**_*α*_, modified frequency Ω_mat_, and modified coupling strength *G*_MoC_. (b) Permittivity of the material inside the cavity, obtained from the classical SpC model (blue solid line, Eq. (39)) and the MoC model (red solid line, Eq. (37)), for the collective coupling strength *G* = 0.3 *ω*_cav_.

The previous two examples illustrate the procedure for connecting the variables in the SpC and MoC models to physical observables. In both cases, the optical cavity was coupled to a single quantum emitter, a very challenging situation for experimentally reaching the ultrastrong coupling regime. An alternative approach to access the necessary coupling strengths consists in filling a cavity with many molecules or with a material supporting a well-defined excitation (such as a phononic resonance) [54], [80], [81]. We consider in this section a homogeneous ensemble of molecular emitters as the material that interacts with resonant transverse electromagnetic modes of a Fabry–Pérot cavity (left sketch in Figure 5(a)), a system of significant relevance in experiments [5], [46], [82], [83]. Each molecule presents a vibrational excitation that is modeled as a dipole of induced dipole moment **d**_*i*_ (we focus here on the case of molecular emitters for specificity, but the same derivation can also be applied to phononic or similar materials by focusing on the induced dipole moment associated to each unit cell). We consider that all molecular emitters are identical and thus have the same oscillator strength *f*_dip_ and resonant frequency *ω*_dip_. We use the subindex *dip* to emphasize that, at this stage, we are considering the individual molecular dipoles and not the whole material (the full ensemble of molecular emitters) involved in the coupling. For simplicity, we assume that there are *N*_dip_ molecular emitters distributed homogeneously. The electromagnetic modes of the Fabry–Pérot cavity are standing waves with vector potential **A**_*α*_ and frequency *ω*_cav,*α*_, where all *α* modes are orthogonal.

Following the relations between the observables and oscillators given in Section 3.1, we represent each vibrational dipole as a harmonic oscillator with oscillation amplitude $x_{\text{dip},i}=\frac{\left|d_{i}\right|}{\sqrt{f_{\text{dip}}}}$ and each cavity mode with the variable $x_{\text{cav},\alpha }=\sqrt{\varepsilon_{0}V_{\text{eff}}}A_{\alpha }$ , where $A_{\alpha }$ is the maximum amplitude of the vector potential of the *α* mode. Notably, this system encompasses the two types of interaction discussed in the previous subsections: (i) each induced dipole *i* is coupled to all other dipoles *j* (through the direct Coulomb molecule–molecule interaction) following the SpC model, where the coupling strength $g_{\text{SpC}}^{\left(i,j\right)}$ is given by Eq. (28); (ii) each induced dipole *i* is coupled to all transverse cavity modes *α* according to the MoC model with coupling strength $g_{\text{MoC}}^{\left(\alpha ,i\right)}=\frac{1}{2}\sqrt{\frac{f_{\text{dip}}}{\varepsilon_{0}V_{\text{eff}}}}\Xi_{\alpha }\left(r_{i}\right)\cos\theta_{\alpha ,i}$ (see Section 3.1), where Ξ_*α*_(**r**_*i*_) is the normalized amplitude value of the cavity field at the position of molecular emitter *i* and *θ*_(*α*,*i*)_ is the angle between the orientation of the induced dipole moment of the *i*th molecular emitter and the polarization of each cavity mode. We assume that all molecular emitters are oriented in the same direction as the cavity field, and thus cos *θ*_*α*,*i*_ = 1 for all *α* and *i*. All the interactions present in this system are shown schematically in the left panel of Figure 5(a). To combine all couplings in a single model, we just include in the harmonic oscillator equations the coupling terms associated with the longitudinal dipole–dipole interactions (SpC model, Eq. (8)) and with the interaction of the molecular emitters with the transverse cavity modes (MoC model, Eq. (11)). The resulting equations are

(33a) $$\begin{array}{ll} & {\ddot{x}}_{\text{dip},i}+\omega_{\text{dip}}^{2}x_{\text{dip},i}+\underset{\alpha }{\sum }2g_{\text{MoC}}^{\left(\alpha ,i\right)}{\dot{x}}_{\text{cav},\alpha }+\underset{j\neq i}{\sum }2\omega_{\text{dip}}g_{\text{SpC}}^{\left(i,j\right)}x_{\text{dip},j}=0, \end{array}$$

(33b) $${\ddot{x}}_{\text{cav},\alpha }+\omega_{\text{cav},\alpha }^{2}x_{\text{cav},\alpha }-\underset{i}{\sum }2g_{\text{MoC}}^{\left(\alpha ,i\right)*}{\dot{x}}_{\text{dip},i}=0,$$

where the sum extends over all cavity modes (∑_*α*_) and molecular emitters (∑_*i*_ and ∑_*j*_).

The direct calculation of the dynamics of the entire system requires solving *N*_dip_ × *N*_cav_ equations, where *N*_cav_ is the number of cavity modes. However, due to the homogeneity of the material and the orthogonality of the cavity modes, each cavity mode *α* only couples with a collective matter excitation, which is represented by an oscillator of oscillation amplitude *x*_mat,*α*_ ∝∑_*i*_Ξ_*α*_(**r**_*i*_)*x*_dip,*i*_, i.e., the amplitude of the individual molecular oscillators in the collective mode *α* is weighted by the cavity mode field at the same position. *x*_mat,*α*_ thus captures the response of the whole material formed by the ensemble of molecules, as highlighted by the use of the *mat* subindex. The motion of each cavity mode *α* and the associated collective mode can then be obtained by solving the following two coupled equations (see Section S7 in Supplementary Material for the full derivation and the value of the different parameters)

(34a) $$\begin{array}{ll} & {\ddot{x}}_{\text{mat},\alpha }+\left(\omega_{\text{dip}}^{2}+2\omega_{\text{dip}}g_{\text{shift}}\right)x_{\text{mat},\alpha }+2g_{\text{MoC}}^{\max}\sqrt{N_{\text{eff}}}{\dot{x}}_{\text{cav},\alpha }=0, \end{array}$$

(34b) $${\ddot{x}}_{\text{cav},\alpha }+\omega_{\text{cav},\alpha }^{2}x_{\text{cav},\alpha }-2g_{\text{MoC}}^{\max}\sqrt{N_{\text{eff}}}{\dot{x}}_{\text{mat},\alpha }=0.$$

In these equations, *g*_shift_ is a parameter that depends on the values $g_{\text{SpC}}^{\left(i,j\right)}$ and that effectively describes the effect of the molecule–molecule dipolar interactions on the frequency of the *α* collective matter excitation, and $g_{\text{MoC}}^{\max}$ is the maximum coupling strength between a single molecular emitter and the transverse cavity mode, obtained for a molecular emitter placed at the antinodes of the mode. *N*_eff_ is the effective number of molecular emitters that are coupled to the mode (*N*_eff_ = *N*_dip_/2 for a Fabry–Pérot mode). Equation (34) indicates that it is possible to describe the coupling between a cavity mode and a collective molecular excitation by considering only two harmonic oscillators, which are independent of the other cavity and collective molecular modes. The coupling strength between each collective matter excitation and the corresponding cavity mode increases with *N*_eff_ as $G_{\text{MoC}}=g_{\text{MoC}}^{\max}\sqrt{N_{\text{eff}}}$ . This scaling with $\sqrt{N_{\text{eff}}}$ is consistent with the quantum Dicke model [84] and explains the large coupling strengths that have been demonstrated in these systems [54], [85], [86]. Further, the dipole–dipole interaction between the molecular emitters shifts the frequency of the collective excitation from *ω*_dip_ to $\Omega_{\text{mat}}=\sqrt{\omega_{\text{dip}}^{2}+2\omega_{\text{dip}}g_{\text{shift}}}$ (except when the cavity mode presents extremely fast spatial variations, where more complex effects can occur [87]). This shift corresponds to that described by the Clausius–Mossotti model of the permittivity of a material, where the resonances in the permittivity do not occur at the same frequency as that of the individual microscopic polarizable units. Ω_mat_ can be considered as either the result of dressing the excitation of the individual molecular emitters, or as the bare resonance of the whole material formed by the ensemble of molecular emitters. In this paper, we adopt the latter convention, as we are interested in the coupling of cavity photons with the material itself, and not with the individual constituent molecules. Thus, Ω_mat_ is considered as a bare frequency. After the change of variables, we obtain

(35a) $${\ddot{x}}_{\text{mat},\alpha }+\Omega_{\text{mat}}^{2}x_{\text{mat},\alpha }+2G_{\text{MoC}}{\dot{x}}_{\text{cav},\alpha }=0,$$

(35b) $${\ddot{x}}_{\text{cav},\alpha }+\omega_{\text{cav},\alpha }^{2}x_{\text{cav},\alpha }-2G_{\text{MoC}}{\dot{x}}_{\text{mat},\alpha }=0.$$

In this description, each cavity mode *α* only couples to the collective molecular mode where the induced dipoles are polarized following the orientation and spatial distribution Ξ_*α*_(**r**) of the cavity mode field. This collective molecular mode thus has a total induced dipole moment $d_{\alpha }=\frac{1}{\sqrt{N_{\text{eff}}}}\sum_{i}\Xi_{\alpha }\left(r_{i}\right)d_{i}$ , where **d**_*i*_ are the single-molecule induced dipole moments (see Section S7 in Supplementary Material). Importantly, Eq. (35) indicates that the interaction between each cavity mode with the corresponding collective matter mode is described classically within the MoC model. As a consequence, the description of this coupling is fully equivalent to the analysis of the coupling between the same cavity mode and an individual dipole of frequency Ω_mat_ and increased coupling strength *G*_MoC_, as indicated schematically in Figure 5(a), so that the response of the cavity filled by a large number of molecular emitters can be described by adapting the analysis and conclusions in Section 3.1. For example, the expression of the eigenvectors as a function of the contributions from the cavity and collective molecular modes can be obtained in principle using Eq. (24). The electric field inside the cavity corresponding to each hybrid mode could be obtained by noticing that (i) *x*_cav,*α*_ gives the amplitude of the vector potential $A_{\alpha }$ ; (ii) the oscillator *x*_mat,*α*_ is proportional to the induced dipole moment **d**_*α*_, which enables to calculate the individual induced dipole moments **d**_*i*_ by inverting the relation $d_{\alpha }=\frac{1}{\sqrt{N_{\text{eff}}}}\sum_{i}\Xi_{\alpha }\left(r_{i}\right)d_{i}$ for each *α*; and (iii) these single-molecule quantities lead to the polarization density $P\left(r\right)=\frac{d_{i}\left(r\right)}{\Delta V}$ , where Δ*V* is the volume that each individual dipole occupies (Δ*V* is the same for all dipoles).

We have thus shown that the MoC model constitutes the proper description of the coupling between transverse cavity modes and collective matter excitations in homogeneous materials. We further confirm the validity of this model to describe the system by demonstrating that it allows for recovering the typical bulk permittivity of phononic materials or ensembles of molecules and that this cannot be captured by the SpC model. We first note that, according to recent work [54], [88], [89], the dispersion of the cavity–matter system is exactly the same as the bulk dispersion of the material. This enables to relate the spectrum of the MoC model with the bulk permittivity *ɛ*(*ω*) of the material in the following manner: the cavity modes of the bare cavity (without molecular emitters) follow the dispersion of free photons as *ω*_cav,*α*_ = *ck*_*α*_, with *c* the light speed in vacuum and *k*_*α*_ the wavevector that is determined by the length *L*_cav_ of the Fabry–Pérot cavity (for perfect mirrors) as *k*_*α*_ = *n*_*α*_ *π*/*L*_cav_, for an integer *n*_*α*_ and normal incidence. For the cavity filled with molecular emitters, the frequency of each cavity mode of wavevector *k*_*α*_ is modified from *ω*_cav,*α*_ to $\omega =\frac{ck_{\alpha }}{\sqrt{\varepsilon \left(\omega \right)}}=\frac{\omega_{\text{cav},\alpha }}{\sqrt{\varepsilon \left(\omega \right)}}$ due to the permittivity of the material. According to the discussion above, these *ω* values must be equal to the eigenfrequencies *ω*_±,MoC_ of the MoC model. From Eq. (12), we know that the MoC eigenfrequencies and the bare cavity frequencies are related as $\left(\omega_{\text{cav},\alpha }^{2}-\omega_{\pm ,\text{MoC}}^{2}\right)\left(\Omega_{\text{mat}}^{2}-\omega^{2}\right)-4G_{\text{MoC}}^{2}\omega_{\pm ,\text{MoC}}^{2}=0$ . We can rewrite this relation as

(36) $$\omega_{\pm ,\text{MoC}}^{2}=\frac{\omega_{\text{cav},\alpha }^{2}}{1+\frac{4G_{\text{MoC}}^{2}}{\Omega_{\text{mat}}^{2}-\omega^{2}}}.$$

By comparing Eq. (36) with the previous relation $\omega =\frac{ck_{\alpha }}{\sqrt{\varepsilon \left(\omega \right)}}=\frac{\omega_{\text{cav},\alpha }}{\sqrt{\varepsilon \left(\omega \right)}}$ , it is possible to identify the permittivity of the material in the cavity as

(37) $$\varepsilon_{\text{MoC}}\left(\omega \right)=1+\frac{4G_{\text{MoC}}^{2}}{\Omega_{\text{mat}}^{2}-\omega^{2}}.$$

Equation (37) is the same that was discussed by Hopfield [48] and can be compared with the permittivity of polar materials. The latter can often be described in a range of infrared frequencies as

(38) $$\varepsilon \left(\omega \right)=\varepsilon_{\infty }\left(1+\frac{\omega_{\text{LO}}^{2}-\omega_{\text{TO}}^{2}}{\omega_{\text{TO}}^{2}-\omega^{2}}\right),$$

where *ω*_TO_ and *ω*_LO_ are the frequencies of the transverse optical and longitudinal optical phonons, respectively [90]. Thus, the MoC model recovers the permittivity of a polar material or an ensemble of molecules, with the correspondences Ω_mat_ = *ω*_TO_ and $G_{\text{MoC}}=\sqrt{\frac{\omega_{\text{LO}}^{2}-\omega_{\text{TO}}^{2}}{4}}$ . The only difference is that Eq. (37) does not include the high-frequency permittivity *ɛ*_∞_ because this parameter originates from additional molecular excitations that are not considered in our model. In order to show that the MoC model is the only model with bare frequencies that correctly describes the permittivity of these materials, we derive the permittivity *ɛ*_SpC_(*ω*) obtained within the SpC model by repeating the procedure with Eq. (9). We obtain:

(39) $$\varepsilon_{\text{SpC}}\left(\omega \right)={\left(\frac{2G_{\text{SpC}}^{2}\Omega_{\text{mat}}}{\omega \left(\Omega_{\text{mat}}^{2}-\omega^{2}\right)}+\sqrt{1+{\left(\frac{2G_{\text{SpC}}^{2}\Omega_{\text{mat}}}{\omega \left(\Omega_{\text{mat}}^{2}-\omega^{2}\right)}\right)}^{2}}\right)}^{2},$$

which does not follow the standard form of the permittivity (Eq. (38)).

For comparison, we plot in Figure 5(b) the permittivities obtained with the MoC model (red solid line, Eq. (37)) and the SpC model (blue solid line, Eq. (39)), as a function of the normalized frequency *ω*/Ω_mat_, with *G* = 0.3 Ω_mat_. The distinct behavior of permittivity predicted by the two models becomes evident when comparing their Reststrahlen bands. The Reststrahlen band represents the frequency range where electromagnetic waves cannot propagate in the bulk material (and also correspond to the maximum polaritonic gap achievable through the coupling of matter excitations with optical modes in dielectric resonators [54], [91]). The Reststrahlen band is delimited in polar materials by the phonon frequencies *ω*_TO_ and *ω*_LO_. The MoC model describes the Reststrahlen band appropriately, because the permittivity is negative in the range $\omega \in \left(\Omega_{\text{mat}},\sqrt{\Omega_{\text{mat}}^{2}+4G_{\text{MoC}}^{2}}\right)=\left(\omega_{\text{TO}},\omega_{\text{LO}}\right)$ (highlighted by the green area in Figure 5(b)). In contrast, the permittivity *ɛ*_SpC_ associated with the SpC model is non-negative for all frequencies and thus is unable to describe the presence of a Reststrahlen band. As an additional difference between both models, only the MoC model results in a permittivity that does not diverge in the *ω* → 0 limit, in agreement with the expected behavior (Eq. (38)). We further discuss the classical modeling of the Reststrahlen band in Section S8 of the Supplementary Material, where we do not require the use in the coupled harmonic oscillator equations of the resonant frequency of the *bare* excitation of the material (Ω_mat_ = *ω*_TO_) and cavity (which is the choice that defines the MoC, see discussion at the end of Section 2.1 and before Eq. (35)). We show that, without this constraint, i.e., by using a *dressed* excitation of the material or the cavity, the Reststrahlen band can also be accurately described by alternative models where the coupling term is proportional to the oscillation amplitude.

In this subsection, we have focused on the coupling with (harmonic) vibrations and phonons. Still, the discussion holds validity for other dipolar matter excitations, independent of their physical origin, such as molecular excitons. The main difference between excitons and vibrations is that the former are two-level systems (fermionic transitions), which, when the number of coupled molecules is low enough, introduces many nonlinear effects not included in classical harmonic oscillator models. However, when many molecules are present, the collective excitation is bosonic according to the Holstein–Primakoff transformation [92]. Therefore, while the discussions in Sections 3.1 and 3.2 are valid for harmonic excitations or for obtaining properties such as eigenvalues and electric field distribution under weak illumination, the discussion in this subsection is applicable more broadly.

## 4 Conclusions

We have analyzed the application of classical coupled harmonic oscillator models to describe nanophotonic systems under ultrastrong coupling and the connection of these models with quantum descriptions. This study focuses on the two classical models typically used in this context, here referred to as the Spring Coupling (SpC) and Momentum Coupling (MoC) models, where the difference relies on whether the coupling term is proportional to the oscillation amplitudes (SpC model) or to their time derivatives (MoC model). The choice between these models typically does not have significant consequences in the weak and strong coupling regimes, where both can be approximated to the same linearized model (this approximation is discussed in the Supplementary Material and is equivalent to the rotating-wave approximation in quantum models). However, the SpC and MoC models result in very different eigenvalues in the ultrastrong coupling regime. We show that the SpC model describes light–matter coupling induced by Coulomb interactions, such as those governing the interaction between different quantum emitters and between quantum emitters and small plasmonic nanoparticles, and that this model results in the same eigenvalue spectra as the quantum Hopfield Hamiltonian without diamagnetic term. On the other hand, the MoC model reproduces the spectra of systems for which the diamagnetic term should be present in the Hamiltonian, corresponding to systems where matter excitations interact with transverse electromagnetic fields (for example, in conventional dielectric cavities). The SpC and MoC models thus result in the same spectra of ultrastrongly coupled nanophotonic systems as a cavity-QED description without and with diamagnetic term, respectively, but using a simpler framework. These two classical models consider the bare cavity and matter frequencies, but we generalize the discussion in the Supplementary Material (Section S2) to alternative models of classical oscillators. This generalized analysis indicates that dressing the frequencies allows us to transform coupled harmonic oscillator models where the coupling is proportional to the oscillation amplitudes to equivalent equations with coupling proportional to their time derivatives and vice versa.

Additionally, classical oscillator models are typically used to calculate the eigenvalues of the system, but we discuss how they also provide other experimentally measurable magnitudes in three canonical systems of nanophotonics. We first show that the MoC model can be applied to calculate the electric field distribution of the two hybrid modes of a dielectric cavity filled by a single quantum emitter. Next, we use the SpC model to calculate the near-field distribution and the far-field scattering spectra of a quantum emitter located near a metallic nanoparticle. Last, the two models are combined to consider an ensemble of molecules inside a dielectric cavity. The molecules interact with each other through Coulomb interactions (SpC model) and also with the transverse electromagnetic modes of the dielectric cavity (MoC model). In this case, we show that the system response can be obtained by considering that each transverse cavity mode interacts with a collective molecular excitation. The only effect of the molecule–molecule dipolar interactions is to modify the effective frequency of these collective excitations, and the MoC model describes the ultrastrong coupling between these collective excitations and the cavity modes. Interestingly, the MoC model enables to recover correctly the permittivity and bulk dispersion of the material filling the cavity, and thus also the Reststrahlen band observed in polar materials, which is not the case for the SpC model. Alternative coupled harmonic oscillator models of the bulk dispersion are discussed in Section S8 of the Supplementary Material. Our work hence advances the exploration of classical descriptions of the ultrastrong coupling regime. It opens the possibility of simplifying the analysis of a wide variety of complex systems often described with quantum models.

## Supplementary Material

Supplementary Material Details
