# Supplementary material for: Description of ultrastrong light–matter interaction through coupled harmonic oscillator models and their connection with cavity-QED Hamiltonians
Source: Nanophotonics. 2025 Mar 11;14(11):2031–52. doi: 10.1515/nanoph-2024-0528 (PMC12133317; doi:10.1515/nanoph-2024-0528)
Supplement: Supplementary file 1 — Supplementary Material Details [file j_nanoph-2024-0528_suppl_001.pdf]

Supplementary Material for: Description of ultrastrong light-matter interaction through coupled harmonic oscillator models and their connection with cavity-QED Hamiltonians

Unai Muniain*[1], Javier Aizpurua[1,2,3], Rainer Hillenbrand[2,3,4], Luis Martín-Moreno[5,6] and Ruben Esteban*[1,7]

[1]*Donostia International Physics Center, Paseo Manuel de Lardizabal 4, 20018 Donostia-San Sebastián, Spain*

[2]*IKERBASQUE, Basque Foundation for Science, María Díaz de Haro 3, 48013 Bilbao, Spain*

[3]*Department of Electricity and Electronics, University of the Basque Country (UPV/EHU), 48940 Leioa, Spain*

[4]*CIC nanoGUNE BRTA, Tolosa Hiribidea 76, 20018 Donostia-San Sebastián, Spain*

[5]*Departamento de Física de la Materia Condensada, Universidad de Zaragoza, 50009 Zaragoza, Spain*

[6]*Instituto de Nanociencia y Materiales de Aragón (INMA), CSIC-Universidad de Zaragoza, 50009 Zaragoza, Spain*

[7]*Centro de Física de Materiales (CFM-MPC), CSIC-UPV/EHU, Paseo Manuel de Lardizabal 5, 20018 Donostia-San Sebastián, Spain*

Corresponding authors: Unai Munian (unaimuni@gmail.com); Ruben Esteban (ruben.esteban@ehu.eus)

Contents

S1 Derivation of the equations of motion in the classical coupled harmonic oscillator models **S2**

S2 Alternative classical models of coupled harmonic oscillators **S7**

S2.1 Alternative model of a matter excitation interacting with transverse cavity modes obtained within the Coulomb gauge S7

S2.2 Alternative model of a matter excitation interacting with transverse cavity modes obtained within the dipole gauge S9

S2.3 Alternative model of a molecular emitter interacting with a metallic nanoparticle . . . S10

S3 Comparison between cavity-QED Hamiltonians of different systems and gauges **S11**

S4 Summary of classical models and their connection with cavity-QED Hamiltonians **S12**

S5 Linearized Model **S14**

S6 Evolution of the eigenvalues for a different choice of coupling strength **S15**

S7 Transformation from individual to collective oscillators in the description of homogeneous materials in Fabry-Pérot cavities **S16**

S8 Reststrahlen band **S19**

S1 Derivation of the equations of motion in the classical coupled harmonic oscillator models

In the main article, we derive the classical models of coupled harmonic oscillators from the cavity Quantum Electrodynamics (QED) Hamiltonians. In this supplementary section, we derive in detail the equations of motion of the classical harmonic oscillators within a classical electromagnetic description that departs from the classical Lagrangian (Sec. S3 shows how to use this approach to obtain also the cavity-QED Hamiltonians).

We start this derivation from the general classical Lagrangian representing charges and electromagnetic fields, which we then particularize for the specific systems we analyze in the main article. Afterward, we show that the Spring Coupling (SpC) and the Momentum Coupling (MoC) models defined in the main article are obtained from the Euler-Lagrange equations of motion of these Lagrangians. Thus, a fully classical description is enough to model ultrastrong coupling in different nanophotonic systems without the need to use any quantum model. Last, we discuss how to introduce laser illumination into the SpC Model (necessary for Sec. 3.2 of the main text) and confirm the validity of the SpC model by comparing it with a an alternative description based on classical polarizabilities.

The form of the electromagnetic Lagrangian depends on the gauge. We choose the Coulomb gauge, which leads to the following expression [1]:

$$L_{\text{Cou}} = \sum_j \frac{1}{2} m_j \dot{\mathbf{r}}_j^2 - \sum_{i,j>i} \frac{q_i q_j}{4\pi\varepsilon_0 |\mathbf{r}_i - \mathbf{r}_j|} + \int \left[\frac{\varepsilon_0}{2} (|\dot{\mathbf{A}}(\mathbf{r})|^2 - c^2 |\nabla \times \mathbf{A}(\mathbf{r})|^2) + \mathbf{j}(\mathbf{r}) \cdot \mathbf{A}(\mathbf{r}) \right] d\mathbf{r}. \tag{S1}$$

In this Lagrangian, the electromagnetic degrees of freedom are encapsulated in the dynamical field variable $\mathbf{A}(\mathbf{r})$, which represents the vector potential of the fields, with the condition $\nabla \cdot \mathbf{A} = 0$ due to the choice of gauge. The energy of these fields is scaled by the vacuum permittivity ε_0 and the light speed in vacuum c (for simplicity, we assume in this section that the material filling the cavity is vacuum). On the other hand, all the dynamics related to the matter structure are expressed by the spatial positions $\mathbf{r}_i$, mass m_i, and charge q_i of each point-like charge indexed by i. Each point charge interacts with all the others according to the Coulomb potential energy (second term on the right-hand side) and with the transverse electromagnetic fields (according to the $\int \mathbf{j}(\mathbf{r}) \cdot \mathbf{A}(\mathbf{r}) d\mathbf{r}$ term, where $\mathbf{j}(\mathbf{r}) = \sum_i q_i \dot{\mathbf{r}}_i \delta(\mathbf{r} - \mathbf{r}_i)$ is the current density at any position $\mathbf{r}$).

The equations of motion obtained from the Lagrangian in Eq. (S1) for the variables $\mathbf{A}(\mathbf{r})$ and $\mathbf{r}_i$ are equivalent to Maxwell's equation for a general system. We are interested in obtaining the equations of motion that describe the dynamics of systems formed by molecules or similar quantum emitters interacting with cavity modes in the strong and the ultrastrong coupling regimes. First, we focus on the terms of the Lagrangian related to the electromagnetic field (which in the Coulomb gauge is entirely described with the vector potential $\mathbf{A}$). The vector potential is separated into the components $\mathbf{A}_\alpha(\mathbf{r})$ of all transverse modes α of the cavity as $\mathbf{A}(\mathbf{r}) = \sum_\alpha \mathbf{A}_\alpha(\mathbf{r}) = \sum_\alpha \mathcal{A}_\alpha \Xi_\alpha(\mathbf{r}) \mathbf{n}_\alpha(\mathbf{r})$. For each α index, the field is polarized at any position in the direction determined by the unit vector $\mathbf{n}_\alpha(\mathbf{r})$, the maximum scalar amplitude is given by $\mathcal{A}_\alpha$ and the fields have spatial distribution $\Xi_\alpha(\mathbf{r})$, normalized so that $\Xi_\alpha(\mathbf{r}) = 1$ in the position where the field is maximum. Further, we consider that the α modes form an orthogonal basis, and the integral of the field distribution over space gives the effective volume of the mode, i.e.

$$\int \Xi_\alpha(\mathbf{r}) \Xi_{\alpha'}^*(\mathbf{r}) \mathbf{n}_\alpha(\mathbf{r}) \cdot \mathbf{n}_{\alpha'}(\mathbf{r}) \, d\mathbf{r} = V_{\text{eff},\alpha} \delta_{\alpha,\alpha'}. \tag{S2}$$

By taking into account the decomposition of the modes and their orthogonality, the terms of the Lagrangian of Eq. (S1) only related to the electromagnetic fields are written as

$$\int \frac{\varepsilon_0}{2}\left(\left|\sum_\alpha \dot{\mathcal{A}}_\alpha \Xi_\alpha(\mathbf{r})\mathbf{n}_\alpha\right|^2 - c^2\left|\nabla\times\sum_\alpha \mathcal{A}_\alpha \Xi_\alpha(\mathbf{r})\mathbf{n}_\alpha\right|^2\right) d\mathbf{r} = \sum_\alpha \frac{\varepsilon_0 V_{\text{eff},\alpha}}{2}\left(\dot{\mathcal{A}}_\alpha \dot{\mathcal{A}}^*_\alpha - \omega^2_{\text{cav},\alpha}\mathcal{A}_\alpha\mathcal{A}^*_\alpha\right). \tag{S3}$$

We now focus on the terms of the Lagrangian associated with the matter degrees of freedom to describe the matter excitations. We model the material as an ensemble of dipoles indexed by j, each formed by two point charges that have the same mass m_j and opposite charges and are placed in positions $\mathbf{r}_{j+}$ and $\mathbf{r}_{j-}$ (representing e.g. the simplest description of a quantum emitter). At equilibrium, $\mathbf{r}_{j+} - \mathbf{r}_{j-} = \mathbf{r}_j^{\text{eq}}$, where $\mathbf{r}_j^{\text{eq}}$ can take into account the coupling with other dipoles. For example, when modeling a complex molecule $\mathbf{r}_j^{\text{eq}}$ would be obtained including the interaction between all charges forming the molecule. We make the harmonic approximation to the Coulomb potential experienced by each dipole with respect to the equilibrium position: $\approx \frac{1}{2}m_{\text{red}}\omega^2_{\text{mat}}|\mathbf{r}_{j+} - \mathbf{r}_{j-} - \mathbf{r}_j^{\text{eq}}|^2$, where m_{red} is the reduced mass of the dipole. We also assume that the mass center of the dipole is static at position $\mathbf{r}_j = \frac{\mathbf{r}_{j+}+\mathbf{r}_{j-}}{2}$, while the distance between point charges from the equilibrium position, i.e., $\mathbf{l}_j = \mathbf{r}_{j+} - \mathbf{r}_{j-} - \mathbf{r}_{\text{eq}}$ and, equivalently, the induced dipole moment $\mathbf{d}_j = q_j\mathbf{l}_j$, evolve in time. From these assumptions, the Coulomb potential energy in the second term in Eq. (S1) includes the harmonic potential energy corresponding to the charges in each dipole and the potential energy due to the interaction between different dipoles. Accordingly, the terms related to the matter degrees of freedom in the Lagrangian transform as

$$\begin{aligned}\sum_j \frac{1}{2}m_j\dot{\mathbf{r}}_j^2 - \sum_{i,j>i}\frac{q_iq_j}{4\pi\varepsilon_0|\mathbf{r}_i-\mathbf{r}_j|} =& \sum_j\left(\frac{1}{2}\frac{m_{\text{red},j}}{q_j^2}\dot{d}_j^2 - \frac{1}{2}\frac{m_{\text{red},j}}{q_j^2}\omega^2_{\text{mat},j}d_j^2\right)\\ &- \sum_{i,j>i}\frac{1}{4\pi\varepsilon_0|\mathbf{r}_i-\mathbf{r}_j|^3}\left[\mathbf{d}_i\cdot\mathbf{d}_j - 3(\mathbf{d}_i\cdot\mathbf{n}_{\mathbf{r}ij})(\mathbf{d}_j\cdot\mathbf{n}_{\mathbf{r}ij})\right],\end{aligned} \tag{S4}$$

with $d_j = |\mathbf{d}_j|$ and the unit vector $\mathbf{n}_{\mathbf{r}ij} = \frac{\mathbf{r}_j-\mathbf{r}_i}{|\mathbf{r}_j-\mathbf{r}_i|}$. Equation (S4) has been derived using the harmonic approximation of the dipolar potential and, as a consequence, all terms of the Lagrangian that do not account for light-matter interaction are quadratic with respect to the amplitudes of the vector potential and their time derivatives (Eq. (S3)), or with respect to the induced dipole moments and their time derivatives (Eq. (S4)). Therefore, if light and matter were uncoupled, the dynamical evolution of these variables would be the same as that of free harmonic oscillators. We now discuss how the interaction between the cavity modes and the dipoles affects the equations of motion. The coupling of each dipole with the transverse fields of the cavity appears in the Lagrangian as

$$\begin{aligned}\int \mathbf{j}\cdot\mathbf{A}\,d\mathbf{r} &= \int\left(\sum_j q_j\dot{\mathbf{r}}_{j+}\delta(\mathbf{r}-\mathbf{r}_{j+}) - q_j\dot{\mathbf{r}}_{j-}\delta(\mathbf{r}-\mathbf{r}_{j-})\right)\left(\sum_\alpha \mathcal{A}_\alpha\Xi_\alpha(\mathbf{r})\mathbf{n}_\alpha\right)d\mathbf{r}\\ &= \sum_{j,\alpha} q_j[\mathbf{r}_{j+}\Xi_\alpha(\dot{\mathbf{r}}_{j+}) - \dot{\mathbf{r}}_{j-}\Xi_\alpha(\mathbf{r}_{j-})]\mathcal{A}_\alpha\mathbf{n}_\alpha \approx \sum_{j,\alpha}\mathcal{A}_\alpha\Xi_\alpha(\mathbf{r}_j)\dot{\mathbf{d}}_j\cdot\mathbf{n}_\alpha\end{aligned} \tag{S5}$$

In the last step, we have performed the long-wavelength approximation, so that the fields do not vary in the length scale of each dipole, i.e., $\Xi(\mathbf{r}_{j+}) \approx \Xi(\mathbf{r}_{j-})$ for any j. The total Lagrangian of the system

in the Coulomb gauge reads

$$L_{\text{Cou}}(d_j,\dot{d}_j,\mathcal{A}_\alpha,\dot{\mathcal{A}}_\alpha,\mathcal{A}^*_\alpha,\dot{\mathcal{A}}^*_\alpha)=\sum_\alpha \frac{\varepsilon_0 V_{\text{eff},\alpha}}{2}\left(\dot{\mathcal{A}}_\alpha\dot{\mathcal{A}}^*_\alpha-\omega^2_{\text{cav},\alpha}\mathcal{A}_\alpha\mathcal{A}^*_\alpha\right)+\sum_j\frac{1}{2}\frac{1}{f_{\text{mat},j}}\left(\dot{d}_j^2-\omega^2_{\text{mat},j}d_j^2\right)$$
$$+\sum_{j,\alpha}\mathcal{A}_\alpha\dot{d}_j\Xi_\alpha(\mathbf{r}_j)\cos\theta_{\alpha,j}-\sum_{i,j}d_id_j\frac{\mathbf{n}_{\mathbf{d}i}\cdot\mathbf{n}_{\mathbf{d}j}-3(\mathbf{n}_{\mathbf{d}i}\cdot\mathbf{n}_{\mathbf{r}ij})(\mathbf{n}_{\mathbf{d}j}\cdot\mathbf{n}_{\mathbf{r}ij})}{4\pi\varepsilon_0|\mathbf{r}_i-\mathbf{r}_j|^3}, \tag{S6}$$

where $\mathbf{n}_{\mathbf{d}j}=\frac{\mathbf{d}_j}{|\mathbf{d}_j|}$, $\theta_{\alpha,j}$ is the angle between the induced dipole moment $\mathbf{d}_j$ and the direction $\mathbf{n}_\alpha$ of the electric field in the mode α, and $f_{\text{mat}}=\frac{q_j^2}{m_{\text{red}}}$ is the oscillator strength of the jth dipole.

From the Lagrangian L_{Cou} of Eq. (S6), we can derive the equations of motion of the classical coupled harmonic oscillators by calculating the Euler-Lagrange equations, $\frac{d}{dt}\frac{\partial L_{\text{Cou}}}{\partial\dot{x}}-\frac{\partial L_{\text{Cou}}}{\partial x}=0$, for $x\in\{d_j,\mathcal{A}^*_\alpha\}$. The resulting equations of motion are

$$\ddot{\mathcal{A}}_\alpha+\omega^2_{\text{cav},\alpha}\mathcal{A}_\alpha-\sum_j\dot{d}_j\frac{\Xi_\alpha(\mathbf{r}_j)\cos\theta_{\alpha,j}}{\varepsilon_0V_{\text{eff},\alpha}}=0, \tag{S7a}$$

$$\ddot{d}_j+\omega^2_{\text{mat},j}d_j+f_{\text{mat},j}\sum_{i\neq j}\frac{\mathbf{n}_{\mathbf{d}i}\cdot\mathbf{n}_{\mathbf{d}j}-3(\mathbf{n}_{\mathbf{d}i}\cdot\mathbf{n}_{\mathbf{r}ij})(\mathbf{n}_{\mathbf{d}j}\cdot\mathbf{n}_{\mathbf{r}ij})}{4\pi\varepsilon_0|\mathbf{r}_i-\mathbf{r}_j|^3}d_i+\sum_\alpha\dot{\mathcal{A}}_\alpha f_{\text{mat},j}\Xi^*_\alpha(\mathbf{r}_j)\cos\theta_{\alpha,j}=0. \tag{S7b}$$

These equations account for all dipole-cavity and dipole-dipole interactions, as analyzed in Sec. 3.3 of the main article. To show how to obtain the MoC and SpC models, we focus on the two canonical examples analyzed in Secs. 3.1 and 3.2 of the main article:

- Coupling between a quantum emitter and a transverse mode of a dielectric cavity (Sec. 3.1): By considering a single transverse mode α of the cavity interacting with one molecular emitter with induced dipole moment d, all Coulomb interactions in Eq. (S7) are eliminated. The equations of motion become

$$\ddot{\mathcal{A}}+\omega^2_{\text{cav}}\mathcal{A}-\dot{d}\frac{\Xi(\mathbf{r}_{\text{mat}})\cos\theta}{\varepsilon_0V_{\text{eff}}}=0, \tag{S8a}$$
$$\ddot{d}+\omega^2_{\text{mat}}d+\dot{\mathcal{A}}f_{\text{mat}}\Xi^*(\mathbf{r}_{\text{mat}})\cos\theta=0. \tag{S8b}$$

  By replacing here the oscillation amplitudes $x_{\text{cav}}=\mathcal{A}\sqrt{\varepsilon_0V_{\text{eff}}}$ and $x_{\text{mat}}=\frac{d}{\sqrt{f_{\text{mat}}}}$ and introducing the coupling strength

$$g_{\text{MoC}}=\frac{1}{2}\sqrt{\frac{f_{\text{mat}}}{\varepsilon_0V_{\text{eff}}}}\Xi(\mathbf{r}_{\text{mat}})\cos\theta, \tag{S9}$$

  we recover the equations of motion of the MoC model (Eq. (11) in the main article).

- Coupling between a quantum emitter and a plasmonic nanoparticle via Coulomb interactions (Sec. 3.2): We consider that the emitter (a molecule) and the nanoparticle have induced dipole moments d_{mat} and d_{cav}, respectively. Under the quasistatic approximation of the plasmonic response, the vector potential components of all transverse modes are neglected. With this

approximation and for only two dipoles, Eq. (S7) is written as

$$\ddot{d}_{\text{cav}} + \omega_{\text{cav}}^2 d_{\text{cav}} + f_{\text{cav}} \frac{\mathbf{n}_{\mathbf{d}\text{cav}} \cdot \mathbf{n}_{\mathbf{d}\text{mat}} - 3(\mathbf{n}_{\mathbf{d}\text{cav}} \cdot \mathbf{n}_{\mathbf{r}\text{rel}})(\mathbf{n}_{\mathbf{d}\text{mat}} \cdot \mathbf{n}_{\mathbf{r}\text{rel}})}{4\pi\varepsilon_0 |\mathbf{r}_{\text{cav}} - \mathbf{r}_{\text{mat}}|^3} d_{\text{mat}} = 0, \tag{S10a}$$

$$\ddot{d}_{\text{mat}} + \omega_{\text{mat}}^2 d_{\text{mat}} + f_{\text{mat}} \frac{\mathbf{n}_{\mathbf{d}\text{cav}} \cdot \mathbf{n}_{\mathbf{d}\text{mat}} - 3(\mathbf{n}_{\mathbf{d}\text{cav}} \cdot \mathbf{n}_{\mathbf{r}\text{rel}})(\mathbf{n}_{\mathbf{d}\text{mat}} \cdot \mathbf{n}_{\mathbf{r}\text{rel}})}{4\pi\varepsilon_0 |\mathbf{r}_{\text{cav}} - \mathbf{r}_{\text{mat}}|^3} d_{\text{cav}} = 0, \tag{S10b}$$

where $\mathbf{n}_{\mathbf{r}\text{rel}} = \frac{\mathbf{r}_{\text{cav}} - \mathbf{r}_{\text{mat}}}{|\mathbf{r}_{\text{cav}} - \mathbf{r}_{\text{mat}}|}$ is the unitary vector of the relative direction between the nanocavity and the molecular emitter. By replacing $x_{\text{cav}} = \frac{d_{\text{cav}}}{\sqrt{f_{\text{cav}}}}$ and $x_{\text{mat}} = \frac{d_{\text{mat}}}{\sqrt{f_{\text{mat}}}}$, and defining the coupling strength g_{SpC} as

$$g_{\text{SpC}} = \frac{1}{2} \frac{\sqrt{f_{\text{cav}}}\sqrt{f_{\text{mat}}}}{4\pi\varepsilon_0 |\mathbf{r}_{\text{cav}} - \mathbf{r}_{\text{mat}}|^3 \sqrt{\omega_{\text{cav}}\omega_{\text{mat}}}} [\mathbf{n}_{\mathbf{d}\text{cav}} \cdot \mathbf{n}_{\mathbf{d}\text{mat}} - 3(\mathbf{n}_{\mathbf{d}\text{cav}} \cdot \mathbf{n}_{\mathbf{r}\text{rel}})(\mathbf{n}_{\mathbf{d}\text{mat}} \cdot \mathbf{n}_{\mathbf{r}\text{rel}})], \tag{S11}$$

we recover the equations of the SpC model (Eq. (8) in the main article).

Spring coupling model with external laser illumination

In Sec. 3.2 of the main text, the dipolar mode of a metallic nanoparticle is excited by an external laser. We now discuss briefly how to introduce the incident laser field in the model of the interaction of this metallic nanocavity with a quantum emitter, e.g. a molecule. The incident field is treated as a planewave of wavevector $\mathbf{k}_{\text{inc}}$, amplitude $\mathcal{A}_{\text{inc}}$ and frequency ω, with an associated vector potential of the form $\mathbf{A}_{\text{inc}}(\mathbf{r}, t) = \mathcal{A}_{\text{inc}} e^{i\mathbf{k}_{\text{inc}} \cdot \mathbf{r}} e^{-i\omega t}$. Under the quasistatic approximation, all transverse modes α of the system are neglected, and thus the only component of the vector potential considered in the Lagrangian of Eq. (S6) corresponds to the external laser $\mathbf{A}_{\text{inc}}(\mathbf{r}, t)$. With these considerations, the Lagrangian of Eq. (S6) becomes

$$L_{\text{Cou}}^{\text{dip-dip}}(d_{\text{cav}}, \dot{d}_{\text{cav}}, d_{\text{mat}}, \dot{d}_{\text{mat}}) = \frac{1}{2} \frac{1}{f_{\text{cav}}} \left(\dot{d}_{\text{cav}}^2 - \omega_{\text{cav}}^2 d_{\text{cav}}^2 \right) + \frac{1}{2} \frac{1}{f_{\text{mat}}} \left(\dot{d}_{\text{mat}}^2 - \omega_{\text{mat}}^2 d_{\text{mat}}^2 \right)$$
$$- d_{\text{cav}} d_{\text{mat}} \frac{\mathbf{n}_{\mathbf{d}\text{cav}} \cdot \mathbf{n}_{\mathbf{d}\text{mat}} - 3(\mathbf{n}_{\mathbf{d}\text{cav}} \cdot \mathbf{n}_{\mathbf{r}\text{rel}})(\mathbf{n}_{\mathbf{d}\text{mat}} \cdot \mathbf{n}_{\mathbf{r}\text{rel}})}{4\pi\varepsilon_0 |\mathbf{r}_{\text{cav}} - \mathbf{r}_{\text{mat}}|^3} + \mathcal{A}_{\text{inc}} e^{-i\omega t} (\dot{d}_{\text{cav}} \cos\theta_{\text{inc,cav}} + \dot{d}_{\text{mat}} \cos\theta_{\text{inc,mat}}), \tag{S12}$$

where $\theta_{\text{inc,cav}}$ and $\theta_{\text{inc,mat}}$ are the angles between the incident field and the induced dipole moments of the cavity and molecular emitter, respectively. The superscript "dip-dip" emphasizes that we only consider dipole-dipole interactions for this system (under the quasistatic approximation). The dynamics of the variables d_{cav} and d_{mat} are obtained within the Euler-Lagrange equations of Eq. (S12). By calculating these equations of motion and transforming the variables into the oscillation amplitudes $x_{\text{cav}} = \frac{d_{\text{cav}}}{\sqrt{f_{\text{cav}}}}$ and $x_{\text{mat}} = \frac{d_{\text{mat}}}{\sqrt{f_{\text{mat}}}}$, the resulting equations are

$$\ddot{x}_{\text{cav}} + \omega_{\text{cav}}^2 x_{\text{cav}} + \frac{\mathbf{n}_{\mathbf{d}\text{cav}} \cdot \mathbf{n}_{\mathbf{d}\text{mat}} - 3(\mathbf{n}_{\mathbf{d}\text{cav}} \cdot \mathbf{n}_{\mathbf{r}\text{rel}})(\mathbf{n}_{\mathbf{d}\text{mat}} \cdot \mathbf{n}_{\mathbf{r}\text{rel}})}{4\pi\varepsilon_0 |\mathbf{r}_{\text{cav}} - \mathbf{r}_{\text{mat}}|^3} x_{\text{mat}} = -\sqrt{f_{\text{cav}}} \cos\theta_{\text{inc,cav}} \frac{d}{dt} \left(\mathcal{A}_{\text{inc}} e^{-i\omega t} \right), \tag{S13a}$$

$$\ddot{x}_{\text{mat}} + \omega_{\text{mat}}^2 x_{\text{mat}} + \frac{\mathbf{n}_{\mathbf{d}\text{cav}} \cdot \mathbf{n}_{\mathbf{d}\text{mat}} - 3(\mathbf{n}_{\mathbf{d}\text{cav}} \cdot \mathbf{n}_{\mathbf{r}\text{rel}})(\mathbf{n}_{\mathbf{d}\text{mat}} \cdot \mathbf{n}_{\mathbf{r}\text{rel}})}{4\pi\varepsilon_0 |\mathbf{r}_{\text{cav}} - \mathbf{r}_{\text{mat}}|^3} x_{\text{cav}} = -\sqrt{f_{\text{mat}}} \cos\theta_{\text{inc,mat}} \frac{d}{dt} \left(\mathcal{A}_{\text{inc}} e^{-i\omega t} \right). \tag{S13b}$$

Therefore, the incident field is incorporated into the SpC equations of motion (Eq. (8) in the main article) by adding time-dependent force-like terms of amplitude $F_{\text{cav}} = i\omega\mathcal{A}_{\text{inc}}\sqrt{f_{\text{cav}}} \cos\theta_{\text{inc,cav}}$ and $F_{\text{mat}} = i\omega\mathcal{A}_{\text{inc}}\sqrt{f_{\text{mat}}} \cos\theta_{\text{inc,mat}}$ to the nanocavity and the molecular emitter, respectively.

Classical description of the coupling between a molecular emitter and a plasmonic nanocavity based on their polarizability

The interaction of a small metallic nanoparticle with a molecular emitter (or another quantum emitter) can also be described classically by using polarizabilities $\alpha_{\rm cav}$ and $\alpha_{\rm mat}$ for both particles so that the dipole moment induced by the electric field at each position $\mathbf{r}_{\rm cav}$ and $\mathbf{r}_{\rm mat}$ is given by $\mathbf{d}_{\rm cav} = \alpha_{\rm cav}\mathbf{E}(\mathbf{r}_{\rm cav})$ and $\mathbf{d}_{\rm mat} = \alpha_{\rm mat}\mathbf{E}(\mathbf{r}_{\rm mat})$, respectively. We briefly show here that this approach leads to the same equations as the SpC model obtained from the electromagnetic Lagrangian, which supports the validity of the general approach used in the main text. For the cavity mode (plasmon in metallic nanoparticle) and the molecular excitation (or any matter excitation in general), we consider the polarizability given by the Lorentz oscillator model. In the case of the molecular emitter, we assume a single molecular excitation with Lorentzian polarizability centered at resonant frequency $\omega_{\rm mat}$, linewidth determined by the damping frequency γ, and oscillator strength $f_{\rm mat}$. Similarly, we also model the nanocavity response as given by a single plasmonic resonance that follows a Lorentzian-like lineshape (for a Drude permittivity), which is the typical lineshape in the quasistatic regime. This resonance is centered at frequency $\omega_{\rm cav}$ and is characterized by losses κ and oscillator strength $f_{\rm cav}$. The polarizabilities of the plasmonic nanocavity and the molecular emitter are then given by

$$\alpha_{\rm cav}(\omega) = \frac{f_{\rm cav}}{\omega_{\rm cav}^2 - \omega^2 - i\omega\kappa}, \tag{S14a}$$

$$\alpha_{\rm mat}(\omega) = \frac{f_{\rm mat}}{\omega_{\rm mat}^2 - \omega^2 - i\omega\gamma}. \tag{S14b}$$

The dipole moment of the molecular emitter and the nanoparticle is induced by the electric field $\mathbf{E}_{\rm inc}$ of the external laser and also by the electric field generated by either the plasmonic mode ($\mathbf{E}_{\rm cav}$) or the matter excitation in the molecule ($\mathbf{E}_{\rm mat}$), respectively. We then have $\mathbf{d}_{\rm cav} = \alpha_{\rm cav}[\mathbf{E}_{\rm mat}(\mathbf{r}_{\rm cav}) + \mathbf{E}_{\rm inc}]$ and $\mathbf{d}_{\rm mat} = \alpha_{\rm mat}[\mathbf{E}_{\rm cav}(\mathbf{r}_{\rm mat}) + \mathbf{E}_{\rm inc}]$. By inserting in these expressions the polarizabilities given by Eq. (S14) and considering that the quasi-static fields induced by the dipoles excited at the cavity and the molecule follow the dependence,

$$\mathbf{E}_{\rm mat}(\mathbf{r}_{\rm cav}) = \frac{\mathbf{n}_{\mathbf{d}{\rm mat}} - 3(\mathbf{n}_{\mathbf{d}{\rm mat}}\cdot\mathbf{n}_{\mathbf{r}{\rm rel}})\mathbf{n}_{\mathbf{r}{\rm rel}}}{4\pi\varepsilon_0|\mathbf{r}_{\rm cav} - \mathbf{r}_{\rm mat}|^3} d_{\rm mat}, \tag{S15a}$$

$$\mathbf{E}_{\rm cav}(\mathbf{r}_{\rm mat}) = \frac{\mathbf{n}_{\mathbf{d}{\rm cav}} - 3(\mathbf{n}_{\mathbf{d}{\rm cav}}\cdot\mathbf{n}_{\mathbf{r}{\rm rel}})\mathbf{n}_{\mathbf{r}{\rm rel}}}{4\pi\varepsilon_0|\mathbf{r}_{\rm cav} - \mathbf{r}_{\rm mat}|^3} d_{\rm cav}, \tag{S15b}$$

we obtain the expressions of the induced dipole moments

$$(\omega_{\rm cav}^2 - \omega^2 - i\omega\kappa)d_{\rm cav} = f_{\rm cav}\left[\frac{\mathbf{n}_{\mathbf{d}{\rm cav}}\cdot\mathbf{n}_{\mathbf{d}{\rm mat}} - 3(\mathbf{n}_{\mathbf{d}{\rm cav}}\cdot\mathbf{n}_{\mathbf{r}{\rm rel}})(\mathbf{n}_{\mathbf{d}{\rm mat}}\cdot\mathbf{n}_{\mathbf{r}{\rm rel}})}{4\pi\varepsilon_0|\mathbf{r}_{\rm cav} - \mathbf{r}_{\rm mat}|^3} d_{\rm mat} + \mathbf{E}_{\rm inc}\cdot\mathbf{n}_{\mathbf{d}{\rm cav}}\right], \tag{S16a}$$

$$(\omega_{\rm mat}^2 - \omega^2 - i\omega\gamma)d_{\rm mat} = f_{\rm mat}\left[\frac{\mathbf{n}_{\mathbf{d}{\rm cav}}\cdot\mathbf{n}_{\mathbf{d}{\rm mat}} - 3(\mathbf{n}_{\mathbf{d}{\rm cav}}\cdot\mathbf{n}_{\mathbf{r}{\rm rel}})(\mathbf{n}_{\mathbf{d}{\rm mat}}\cdot\mathbf{n}_{\mathbf{r}{\rm rel}})}{4\pi\varepsilon_0|\mathbf{r}_{\rm cav} - \mathbf{r}_{\rm mat}|^3} d_{\rm cav} + \mathbf{E}_{\rm inc}\cdot\mathbf{n}_{\mathbf{d}{\rm mat}}\right]. \tag{S16b}$$

These equations are equivalent to Eq. (S13) in frequency domain, with $x_{\rm cav} = \frac{d_{\rm cav}}{\sqrt{f_{\rm cav}}}$, $x_{\rm mat} = \frac{d_{\rm mat}}{\sqrt{f_{\rm mat}}}$ and using the relation $|\mathbf{E}_{\rm inc}| = |i\omega\mathcal{A}_{\rm inc}|$ that follows from the definition of the vector potential.

S2 Alternative classical models of coupled harmonic oscillators

The discussion of Supplementary Sec. S1 concluded that the classical MoC model describes the coupling of matter excitations with transverse electromagnetic modes, while the SpC model can express dipole-dipole interactions. Crucially, the bare cavity and matter frequencies appear directly in these models without dressing the energies. In this supplementary section, we demonstrate that other classical coupled harmonic oscillator models exist, equivalent to the MoC and SpC models, but involving some frequency dressing (this effect is related to the discussion in Ref. [2] between the dressing of the frequencies and the presence or absence of diamagnetic term). The alternative models depend on the gauge chosen for the classical Lagrangian and Hamiltonian descriptions. We discuss oscillator models in two of the most commonly used gauges: the Coulomb and dipole gauges. We also show that the physical interpretation of the oscillation amplitudes depends on the particular coupled harmonic oscillator model that is used.

More specifically, Secs. S2.1 and S2.2 consider the coupling with transverse modes in dielectric cavities. We derive alternative coupled harmonic oscillator equations that use dressed frequencies and coupling terms proportional to the amplitude of the oscillators (in contrast with the equivalent MoC model, which uses bare frequencies and coupling terms proportional to the time derivatives of the oscillator amplitudes). We first show in Sec. S2.1 how to derive, within the Coulomb gauge, an alternative coupled harmonic oscillator model in which the cavity mode is dressed. Then, in Sec. S2.2, the use of the dipole gauge yields a second alternative coupled harmonic oscillator model with dressed matter excitation and coupling terms again proportional to the oscillations amplitudes.

Afterwards, in Sec S2.3, we consider Coulomb coupling through longitudinal fields, and obtain coupled harmonic oscillator equations with dressing of the matter excitation and coupling term proportional to the time derivatives of the oscillator amplitudes (for comparison, in the equivalent SpC model, the frequencies are the bare ones and the coupling terms are proportional to the oscillator amplitudes of the oscillation models). This section considers the Coulomb gauge, but the dipole gauge yields identical results.

S2.1 Alternative model of a matter excitation interacting with transverse cavity modes obtained within the Coulomb gauge

We first describe the coupling between a transverse electromagnetic mode and a dipolar excitation of a molecule (or another quantum emitter), which is the system discussed in Sec. 3.1 of the main article. The aim is to obtain alternative equations of motion of this system. We start with the classical Lagrangian in the Coulomb gauge given by Eq. (S6), which for the considered system can be expressed as

$$L_{\mathrm{Cou}}^{\mathrm{min\text{-}c}}(d,\dot{d},\mathcal{A},\dot{\mathcal{A}}) = \frac{\varepsilon_0 V_{\mathrm{eff}}}{2}(\dot{\mathcal{A}}^2 - \omega_{\mathrm{cav}}^2\mathcal{A}^2) + \frac{1}{2f_{\mathrm{mat}}}(\dot{d}^2 - \omega_{\mathrm{mat}}^2 d^2) + \mathcal{A}\dot{d}. \tag{S17}$$

To simplify the analytical expressions in the following discussion, we consider Eq. (S17) for a specific case where the molecular emitter is placed in the position of maximum field of the mode and oriented in the same direction as the field polarization so that $\Xi(\mathbf{r}_{\mathrm{mat}})\cos\theta = 1$ (see Sec. S1 for the definition of these parameters). However, the discussion of this section remains valid for other values of $\Xi(\mathbf{r}_{\mathrm{mat}})\cos\theta$.

It has been shown in Supplementary Sec. S1 that the Euler-Lagrange equations derived from Eq. (S17) lead to the MoC model. We use here Hamilton's equations to derive the MoC model in an alternative manner and also to obtain another equivalent classical model of harmonic oscillators. To

first derive the classical Hamiltonian of the system, we obtain the canonical momenta related to the transverse electromagnetic modes and the induced dipole moment in the Coulomb gauge as

$$\Pi_{\text{Cou}} = \frac{\partial L_{\text{Cou}}}{\partial \dot{\mathcal{A}}} = \varepsilon_0 V_{\text{eff}} \dot{\mathcal{A}}, \tag{S18a}$$

$$p_{\text{Cou}} = \frac{\partial L_{\text{Cou}}}{\partial \dot{d}} = \frac{\dot{d}}{f_{\text{mat}}} + \mathcal{A}. \tag{S18b}$$

According to these expressions, the dynamical variable Π_{Cou} expresses the transverse electric field of the cavity modes from the relation $\mathbf{E} = -\frac{\partial \mathbf{A}}{\partial t}$. On the other hand, the relation between the induced dipole moment d and its canonical momentum p_{Cou} is more complicated because p_{Cou} depends not only on d but also on the vector potential. Using Eq. (S18), the calculation of the Hamiltonian $H^{\text{min-c}}_{\text{Cou}} = \dot{\mathcal{A}}\Pi_{\text{Cou}} + \dot{d}p_{\text{Cou}} - L^{\text{min-c}}_{\text{Cou}}$ is straightforward:

$$H^{\text{min-c}}_{\text{Cou}} = \frac{\Pi^2_{\text{Cou}}}{2\varepsilon_0 V_{\text{eff}}} + \frac{1}{2}\varepsilon_0 V_{\text{eff}} \omega^2_{\text{cav}} \mathcal{A}^2 + \frac{f_{\text{mat}}}{2} p^2_{\text{Cou}} + \frac{1}{2}\frac{\omega^2_{\text{mat}}}{f_{\text{mat}}} d^2 - f_{\text{mat}} p_{\text{Cou}} \mathcal{A} + \frac{1}{2} f_{\text{mat}} \mathcal{A}^2. \tag{S19}$$

This expression has the well-known form of the minimal-coupling Hamiltonian. This is the reason why we include the superindex "min-c" in the Lagrangian of Eq. (S17) and in the Hamiltonian of Eq. (S19). We can directly derive the Hamilton's equations of motion of all canonical variables:

$$\dot{\mathcal{A}} = \frac{\partial H^{\text{min-c}}_{\text{Cou}}}{\partial \Pi_{\text{Cou}}} = \frac{\Pi_{\text{Cou}}}{\varepsilon_0 V_{\text{eff}}}, \tag{S20a}$$

$$\dot{\Pi}_{\text{Cou}} = -\frac{\partial H^{\text{min-c}}_{\text{Cou}}}{\partial \mathcal{A}} = -\varepsilon_0 V_{\text{eff}} \omega^2_{\text{cav}} \mathcal{A} + f_{\text{mat}}(p_{\text{Cou}} - \mathcal{A}), \tag{S20b}$$

$$\dot{d} = \frac{\partial H^{\text{min-c}}_{\text{Cou}}}{\partial p_{\text{Cou}}} = f_{\text{mat}}(p_{\text{Cou}} - \mathcal{A}), \tag{S20c}$$

$$\dot{p}_{\text{Cou}} = -\frac{\partial H^{\text{min-c}}_{\text{Cou}}}{\partial d} = -\frac{\omega^2_{\text{mat}}}{f_{\text{mat}}} d_j. \tag{S20d}$$

Hamilton's equations can be used to obtain classical harmonic oscillator models by eliminating two variables, leading to two second-order differential equations. By choosing the variables $\mathcal{A}$ and d to describe the dynamics of the system, we obtain

$$\ddot{\mathcal{A}} + \omega^2_{\text{cav}} \mathcal{A} - \frac{\dot{d}}{\varepsilon_0 V_{\text{eff}}} = 0, \tag{S21a}$$

$$\ddot{d} + \omega^2_{\text{mat}} d + f_{\text{mat}} \dot{\mathcal{A}} = 0. \tag{S21b}$$

This system of equations can be converted into Eq. (11) in the main text, and thus we recover the MoC model. However, there are other possible ways to represent the response of this system with harmonic oscillators. An alternative is to choose the variable p_{Cou} for the matter excitation and $\mathcal{A}$ for the cavity mode. By eliminating the rest of the variables in Eq. (S20), the equations of motion for the chosen variables are written as

$$\ddot{\mathcal{A}} + \left(\omega^2_{\text{cav}} + \frac{f_{\text{mat}}}{\varepsilon_0 V_{\text{eff}}}\right)\mathcal{A} - \frac{f_{\text{mat}}}{\varepsilon_0 V_{\text{eff}}} p_{\text{Cou}} = 0, \tag{S22a}$$

$$\ddot{p}_{\text{Cou}} + \omega^2_{\text{mat}} p_{\text{Cou}} - \omega^2_{\text{mat}} \mathcal{A} = 0. \tag{S22b}$$

With the transformation $x_{\text{cav}} = \sqrt{\varepsilon_0 V_{\text{eff}}}\mathcal{A}$ used in Sec. 3.1 of the main text, and with the new

transformation $x'_{\text{mat}} = \frac{\sqrt{f_{\text{mat}}}}{\omega_{\text{mat}}} p_{\text{Cou}}$, Eq. (S22) becomes

$$\ddot{x}_{\text{cav}} + (\omega_{\text{cav}}^2 + 4g_{\text{MoC}}^2)x_{\text{cav}} - 2g_{\text{MoC}}\omega_{\text{mat}}x'_{\text{mat}} = 0, \tag{S23a}$$

$$\ddot{x}'_{\text{mat}} + \omega_{\text{mat}}^2 x'_{\text{mat}} - 2g_{\text{MoC}}\omega_{\text{mat}}x_{\text{cav}} = 0, \tag{S23b}$$

with the same coupling strength $g_{\text{MoC}} = \frac{1}{2}\sqrt{\frac{f_{\text{mat}}}{\varepsilon_0 V_{\text{eff}}}}$ that is used to describe the cavity-dipole coupling within the MoC model.

Equations (S21) and (S23) (the former corresponding to the MoC model) have been derived for the same system and thus must result in the same response of the system. However, several interesting aspects can be observed. First, in Eq. (S23) x'_{mat} is related to p_{Cou}, while x_{mat} is related to d in the MoC model. Thus, it is important to consider this difference when calculating physical observables, as in Sec. 3.1 of the main text. Second, Eq. (S23) contains coupling terms proportional to the oscillation amplitudes x_{cav} and x'_{mat} (as in the SpC model) instead of to their time derivatives $\dot{x}_{\text{cav}}$ and $\dot{x}_{\text{mat}}$ (as in the MoC model). Last, in Eq. (S23) the frequency of the cavity mode is dressed from ω_{cav} to $\sqrt{\omega_{\text{cav}}^2 + 4g_{\text{MoC}}^2}$. The different coupling terms and the frequency dressing compensate each other, ensuring that Eq. (S23) yields the same result as the MoC model. Therefore, the molecule-dielectric cavity system can be equivalently described using coupling terms proportional to the oscillation amplitudes or to their time derivatives, provided that the frequency of the cavity mode and the physical interpretation of the oscillation amplitudes are modified appropriately.

S2.2 Alternative model of a matter excitation interacting with transverse cavity modes obtained within the dipole gauge

We have shown that the results of the MoC model can be recovered using equations with a different coupling term and a dressed frequency of the cavity mode. Here, we use the dipole gauge to show that we can also obtain equivalent equations by dressing the frequency of the matter excitation. We consider again a single matter excitation and a transverse electromagnetic mode.

The Lagrangian in the Coulomb gauge L_{Cou} of Eq. (S17) can be transformed to any other Lagrangian L' with the operation $L' = L_{\text{Cou}} + \frac{d\mathcal{G}(\mathcal{A},d,t)}{dt}$, by using a general function $\mathcal{G}(\mathcal{A}, d, t)$. In particular, the transformation to the dipole gauge is done with the choice $\mathcal{G} = -d\mathcal{A}$. This is equivalent to the Power-Zienau-Woolley transformation [3] in cavity-QED descriptions, with the unitary operator

$$\hat{U} = \exp\left\{\frac{i}{\hbar}\int \mathbf{P}\cdot\mathbf{A}\,d\mathbf{r}\right\}, \tag{S24}$$

where $\mathbf{P}$ is the polarization density. After applying the gauge transformation to Eq. (S17), the Lagrangian of the system in the dipole gauge is

$$L_{\text{Dip}}^{\text{min-c}}(d, \dot{d}, \mathcal{A}, \dot{\mathcal{A}}) = \frac{\varepsilon_0 V_{\text{eff}}}{2}(\dot{\mathcal{A}}^2 - \omega_{\text{cav}}^2\mathcal{A}^2) + \frac{1}{2f_{\text{mat}}}(\dot{d}^2 - \omega_{\text{mat}}^2 d^2) - \dot{\mathcal{A}}d. \tag{S25}$$

We repeat the procedure implemented in the Coulomb gauge in Sec. S2.1 to obtain the equations of motion of the dynamical variables in the dipole gauge. The canonical momenta are calculated as

$$\Pi_{\text{Dip}} = \frac{\partial L_{\text{Dip}}}{\partial\dot{\mathcal{A}}} = \varepsilon_0 V_{\text{eff}}\dot{\mathcal{A}} - d, \tag{S26a}$$

$$p_{\text{Dip}} = \frac{\partial L_{\text{Dip}}}{\partial\dot{d}} = \frac{\dot{d}}{f_{\text{mat}}}. \tag{S26b}$$

In the dipole gauge, p_{Dip} is only related to the time derivative of the induced dipole moment. However, the canonical momentum associated with the cavity mode, Π_{Dip}, depends on both d and the vector potential, in contrast to the result of the Coulomb gauge (Eq. (S18)). Thus, in the dipole gauge this variable represents the displacement vector $\Pi_{\text{Dip}} \propto |\mathbf{D}| = |\varepsilon_0 \mathbf{E} + \mathbf{P}|$ instead of the electric field of the cavity mode as happens in the Coulomb gauge, where $\Pi_{\text{Cou}} \propto |\mathbf{E}|$. The resulting Hamiltonian in the dipole gauge is

$$H_{\text{Dip}}^{\text{min-c}} = \frac{\Pi_{\text{Dip}}^2}{2\varepsilon_0 V_{\text{eff}}} + \frac{1}{2}\varepsilon_0 V_{\text{eff}} \omega_{\text{cav}}^2 \mathcal{A}^2 + \frac{f_{\text{mat}}}{2} p_{\text{Dip}}^2 + \frac{1}{2}\frac{\omega_{\text{mat}}^2}{f_{\text{mat}}} d^2 + \frac{\Pi_{\text{Dip}} d}{\varepsilon_0 V_{\text{eff}}} + \frac{d^2}{2\varepsilon_0 V_{\text{eff}}}, \tag{S27}$$

with corresponding Hamilton's equations of motion:

$$\dot{\mathcal{A}} = \frac{\partial H_{\text{Dip}}^{\text{min-c}}}{\partial \Pi_{\text{Dip}}} = \frac{\Pi_{\text{Dip}} + d}{\varepsilon_0 V_{\text{eff}}}, \tag{S28a}$$

$$\dot{\Pi}_{\text{Dip}} = -\frac{\partial H_{\text{Dip}}^{\text{min-c}}}{\partial \mathcal{A}} = -\varepsilon_0 V_{\text{eff}} \omega_{\text{cav}}^2 \mathcal{A}, \tag{S28b}$$

$$\dot{d} = \frac{\partial H_{\text{Dip}}^{\text{min-c}}}{\partial p_{\text{Dip}}} = f_{\text{mat}} p_{\text{Dip}}, \tag{S28c}$$

$$\dot{p}_{\text{Dip}} = -\frac{\partial H_{\text{Dip}}^{\text{min-c}}}{\partial d} = -\frac{\omega_{\text{mat}}^2}{f_{\text{mat}}} d - \frac{\Pi_{\text{Dip}} + d}{\varepsilon_0 V_{\text{eff}}}. \tag{S28d}$$

The choice of variables $\mathcal{A}$ and d to obtain second-order differential equations leads to the transformation from Eq. (S28) to Eq. (S21). Therefore, the MoC model is obtained independently of the considered gauge for these variables. On the other hand, with the choice of the variables d and Π_{Dip}, we obtain

$$\ddot{\Pi}_{\text{Dip}} + \omega_{\text{cav}}^2 \Pi_{\text{Dip}} + \omega_{\text{cav}}^2 d = 0 \tag{S29a}$$

$$\ddot{d} + \left(\omega_{\text{mat}}^2 + \frac{f_{\text{mat}}}{\varepsilon_0 V_{\text{eff}}}\right) d + \frac{f_{\text{mat}}}{\varepsilon_0 V_{\text{eff}}} \Pi_{\text{Dip}} = 0. \tag{S29b}$$

This equation can be rewritten in terms of oscillation amplitudes. By using the matter oscillator amplitude $x_{\text{mat}} = \frac{d}{\sqrt{f_{\text{mat}}}}$ and the new cavity oscillator amplitude $x'_{\text{cav}} = \frac{\Pi_{\text{Dip}}}{\sqrt{\varepsilon_0 V_{\text{eff}}} \omega_{\text{cav}}}$, the resulting equations are

$$\ddot{x}'_{\text{cav}} + \omega_{\text{cav}}^2 x'_{\text{cav}} + 2 g_{\text{MoC}} \omega_{\text{cav}} x_{\text{mat}} = 0, \tag{S30a}$$

$$\ddot{x}_{\text{mat}} + (\omega_{\text{mat}}^2 + 4 g_{\text{MoC}}^2) x_{\text{mat}} + 2 g_{\text{MoC}} \omega_{\text{cav}} x'_{\text{cav}} = 0, \tag{S30b}$$

which gives the same results as the MoC model, but with the coupling term proportional to the oscillator oscillation amplitudes x'_{cav} and x_{mat} and with the frequency of the matter excitation dressed, i.e. renormalized, from ω_{mat} to $\sqrt{\omega_{\text{mat}}^2 + 4 g_{\text{MoC}}^2}$.

S2.3 Alternative model of a molecular emitter interacting with a metallic nanoparticle

In Supplementary Secs. S2.1 and S2.2 we have shown that the coupling between a dipolar excitation of a molecular emitter and a transverse cavity mode can be described equivalently with the MoC model (coupling terms proportional to the time derivatives $\dot{x}_{\text{cav}}$ and $\dot{x}_{\text{mat}}$) or with models where the

coupling terms are proportional to the oscillation amplitudes and the frequencies of the oscillators are dressed. Here, we use the Coulomb gauge and show a similar result for the dipole-dipole interaction between one plasmonic mode and one matter excitation in a molecule or any other quantum emitter: this interaction can be described by the SpC model (coupling terms proportional to the oscillation amplitudes $x_{\rm cav}$ and $x_{\rm mat}$) or with alternative equations that contain coupling terms proportional to the time derivatives $\dot{x}_{\rm cav}$ and $\dot{x}_{\rm mat}$, together with dressed frequencies.

We consider the same system analyzed in Sec. 3.2 of the main article, namely, a molecule (or another quantum emitter) placed close to a metallic nanoparticle and coupled to it through the Coulomb interaction. This system is described by the Lagrangian of Eq. (S12) (here we omit laser excitation, i.e. $\mathcal{A}_{\rm inc} = 0$), which leads to the SpC model in Eq. (S10), as discussed in Sec. S1. To obtain the alternative model, we follow the procedure of the previous subsections and first obtain from Eq. (S12) the classical Hamiltonian of the system $H^{\text{dip-dip}} = \dot{d}_{\rm cav}p_{\rm cav} + \dot{d}_{\rm mat}p_{\rm mat} - L^{\text{dip-dip}}_{\rm Cou}$, which is

$$\begin{aligned} H^{\text{dip-dip}} = & \frac{1}{2}f_{\rm cav}p_{\rm cav}^2 + \frac{1}{2}\frac{\omega_{\rm cav}^2}{f_{\rm cav}}d_{\rm cav}^2 + \frac{1}{2}f_{\rm mat}p_{\rm mat}^2 + \frac{1}{2}\frac{\omega_{\rm mat}^2}{f_{\rm mat}}d_{\rm mat}^2 \\ & + d_{\rm cav}d_{\rm mat}\frac{\mathbf{n}_{\mathbf{d}{\rm cav}}\cdot\mathbf{n}_{\mathbf{d}{\rm mat}} - 3(\mathbf{n}_{\mathbf{d}{\rm cav}}\cdot\mathbf{n}_{\mathbf{r}{\rm rel}})(\mathbf{n}_{\mathbf{d}{\rm mat}}\cdot\mathbf{n}_{\mathbf{r}{\rm rel}})}{4\pi\varepsilon_0|\mathbf{r}_{\rm cav}-\mathbf{r}_{\rm mat}|^3}, \end{aligned} \tag{S31}$$

with the canonical momenta $p_{\rm cav} = \frac{\dot{d}_{\rm cav}}{f_{\rm cav}}$ and $p_{\rm mat} = \frac{\dot{d}_{\rm mat}}{f_{\rm mat}}$. The Hamiltonian of Eq. (S31) has been obtained from the Coulomb gauge, but the dipole gauge leads to the same Hamiltonian for this specific system because this change of gauge affects the treatment of the electromagnetic degrees of freedom $\mathcal{A}_\alpha$ associated with the transverse fields. These degrees of freedom are not present when the interaction occurs through Coulomb coupling.

By calculating the equations of motion for the oscillator variables $x_{\rm cav} = \frac{d_{\rm cav}}{\sqrt{f_{\rm cav}}}$ and $x_{\rm mat} = \frac{d_{\rm mat}}{\sqrt{f_{\rm mat}}}$ as in previous subsections, we recover the equations of the SpC model (Eq. (8) in the main text). However, we can again make another choice for the variables to obtain an alternative model of harmonic oscillators. Using the oscillator $x_{\rm cav} = \frac{d_{\rm cav}}{\sqrt{f_{\rm cav}}}$ as before and the new oscillator $x'_{\rm mat} = \frac{\sqrt{f_{\rm mat}}}{\omega_{\rm mat}}p_{\rm mat}$, the equations of motion are

$$\ddot{x}_{\rm cav} + (\omega_{\rm cav}^2 - 4g'^2_{\rm SpC})x_{\rm cav} - 2g'_{\rm SpC}\dot{x}'_{\rm mat} = 0, \tag{S32a}$$

$$\ddot{x}'_{\rm mat} + \omega_{\rm mat}^2 x'_{\rm mat} + 2g'_{\rm SpC}\dot{x}_{\rm cav} = 0, \tag{S32b}$$

with the coupling strength $g'_{\rm SpC} = g_{\rm SpC}\sqrt{\frac{\omega_{\rm cav}}{\omega_{\rm mat}}}$, slightly modified compared to the SpC value $g_{\rm SpC}$ used in Eq. (28) of the main text. We have thus shown that the results of the SpC model can also be obtained with a model where the coupling terms are proportional to the time derivatives $\dot{x}_{\rm cav}$ and $\dot{x}'_{\rm mat}$. In this case the cavity frequency has been renormalized from $\omega_{\rm cav}$ to $\sqrt{\omega_{\rm cav}^2 - 4g'^2_{\rm SpC}}$.

S3 Comparison between cavity-QED Hamiltonians of different systems and gauges

In the previous Supplementary Sections, the SpC, MoC, and alternative coupled harmonic oscillator models are derived from a fully classical description based on Lagrangian and Hamiltonian mechanics. We next quantize the classical Hamiltonians to obtain the cavity-QED Hamiltonians describing the system, including those in the main text. This procedure shows that the cavity-QED Hamiltonians and the corresponding coupled-harmonic oscillator models are directly related.

The coupling between a molecular emitter (or another quantum emitter) and the transverse electromagnetic modes of a dielectric cavity is described by the minimal-coupling Hamiltonian, which for the Coulomb gauge has the classical form of Eq. (S19) and for the dipole gauge it is given by Eq. (S27). We quantize these classical Hamiltonians following the standard rules of quantization (Eqs. (15)-(18) in the main article) and obtain

$$\hat{H}^{\text{min-c}}_{\text{Cou}} = \hbar\omega_{\text{cav}}\left(\hat{a}^\dagger\hat{a}+\frac{1}{2}\right)+\hbar\omega_{\text{mat}}\left(\hat{b}^\dagger\hat{b}+\frac{1}{2}\right)+i\hbar g_{\text{MoC}}\sqrt{\frac{\omega_{\text{mat}}}{\omega_{\text{cav}}}}(\hat{a}+\hat{a}^\dagger)(\hat{b}-\hat{b}^\dagger)+\hbar\frac{g^2_{\text{MoC}}}{\omega_{\text{cav}}}(\hat{a}+\hat{a}^\dagger)^2. \quad \text{(S33)}$$

$$\hat{H}^{\text{min-c}}_{\text{Dip}} = \hbar\omega_{\text{cav}}\left(\hat{a}^\dagger\hat{a}+\frac{1}{2}\right)+\hbar\omega_{\text{mat}}\left(\hat{b}^\dagger\hat{b}+\frac{1}{2}\right)-i\hbar g_{\text{MoC}}\sqrt{\frac{\omega_{\text{cav}}}{\omega_{\text{mat}}}}(\hat{a}-\hat{a}^\dagger)(\hat{b}+\hat{b}^\dagger)+\hbar\frac{g^2_{\text{MoC}}}{\omega_{\text{mat}}}(\hat{b}+\hat{b}^\dagger)^2. \quad \text{(S34)}$$

for the Coulomb and dipole gauges, respectively. In these Hamiltonians, $\hat{a}$ and $\hat{a}^\dagger$ are the annihilation and creation operators of the cavity mode, while $\hat{b}$ and $\hat{b}^\dagger$ are the corresponding operators for the molecular excitations. The main difference between Eqs. (S33) and (S34) is the last quadratic term, which is originated from the vector potential of the electromagnetic mode in the Coulomb gauge, and from the induced dipole moment of the molecule in the dipole gauge, respectively. We further note that the relation between the quantum coupling strength g_{QED} (i.e., the proportionality factor if we write the third term of the Hamiltonians as $\pm i\hbar g_{\text{QED}}(\hat{a}-\hat{a}^\dagger)(\hat{b}+\hat{b}^\dagger)$) and the classical coupling strength g_{MoC} is different for each gauge, with $g_{\text{QED}} = g_{\text{MoC}}\sqrt{\frac{\omega_{\text{mat}}}{\omega_{\text{cav}}}}$ for the Coulomb gauge and $g_{\text{QED}} = g_{\text{MoC}}\sqrt{\frac{\omega_{\text{cav}}}{\omega_{\text{mat}}}}$ for the dipole gauge. We emphasize, however, that the eigenvalues of the two Hamiltonians are identical (given by Eq. (13) in the main text). Further, these two Hamiltonians also lead to identical results for any physical magnitude, once we consider that the operators $\hat{a}$, $\hat{a}^\dagger$, $\hat{b}$ and $\hat{b}^\dagger$ are not equivalent in the two Hamiltonians and are related to a different set of canonical momenta (and thus to different physical magnitudes) in each of them: Π_{Cou} and p_{Cou} given by Eq. (S18) for the Hamiltonian of Eq. (S33), or Π_{Dip} and p_{Dip} given by Eq. (S26) for the Hamiltonian of Eq. (S34).

On the other hand, dipole-dipole interactions (for example, between a metallic nanoparticle and a molecular emitter) are modeled with the following cavity-QED Hamiltonian both in the Coulomb and dipole gauges (obtained by applying the quantization rules to Eq. (S31)):

$$\hat{H}^{\text{dip-dip}} = \hbar\omega_{\text{cav}}\left(\hat{a}^\dagger\hat{a}+\frac{1}{2}\right)+\hbar\omega_{\text{mat}}\left(\hat{b}^\dagger\hat{b}+\frac{1}{2}\right)+\hbar g_{\text{QED}}(\hat{a}+\hat{a}^\dagger)(\hat{b}+\hat{b}^\dagger), \quad \text{(S35)}$$

with $g_{\text{QED}} = g_{\text{SpC}}$. This Hamiltonian does not have any diamagnetic term. Thus it gives different results than the minimal-coupling Hamiltonians of Eqs. (S33) and (S34). Further, the operators $\hat{a}$, $\hat{a}^\dagger$, $\hat{b}$ and $\hat{b}^\dagger$ in Eq. (S35) are related to the induced dipole moments of the nanoparticle and the molecule according to Eq. (17) of the main article.

The analysis of this and the previous sections establishes that the classical coupled harmonic oscillator models and the cavity-QED Hamiltonians can be derived from the same starting point of the Lagrangian in Eq. (S1), and can thus be used to obtain equivalent physical results.

S4 Summary of classical models and their connection with cavity-QED Hamiltonians

Table S1 summarizes all the classical models discussed in Supplementary Secs. S1 and S2, as well as the cavity-QED Hamiltonians discussed in Supplementary Sec. S3. These sections focus on two types of interactions: the coupling between a molecular excitation and transverse electromagnetic modes

Diamagnetic term	Included		Not included
Type of interaction	Dielectric cavity mode-dipole interaction		Dipole-dipole Coulomb interaction
	Coulomb gauge	Dipole gauge	Coulomb and dipole gauge
Cavity QED Hamiltonian	$\hat{H} = \hbar\omega_{\rm cav}\left(\hat{a}^\dagger\hat{a} + \frac{1}{2}\right) + \hbar\omega_{\rm mat}\left(\hat{b}^\dagger\hat{b} + \frac{1}{2}\right) + i\hbar g_{\rm MoC}\sqrt{\frac{\omega_{\rm mat}}{\omega_{\rm cav}}}(\hat{a}+\hat{a}^\dagger)(\hat{b}-\hat{b}^\dagger) + \hbar\frac{g_{\rm MoC}^2}{\omega_{\rm cav}}(\hat{a}+\hat{a}^\dagger)^2$	$\hat{H} = \hbar\omega_{\rm cav}\left(\hat{a}^\dagger\hat{a} + \frac{1}{2}\right) + \hbar\omega_{\rm mat}\left(\hat{b}^\dagger\hat{b} + \frac{1}{2}\right) - i\hbar g_{\rm MoC}\sqrt{\frac{\omega_{\rm cav}}{\omega_{\rm mat}}}(\hat{a}-\hat{a}^\dagger)(\hat{b}+\hat{b}^\dagger) + \hbar\frac{g_{\rm MoC}^2}{\omega_{\rm mat}}(\hat{b}+\hat{b}^\dagger)^2$	$\hat{H} = \hbar\omega_{\rm cav}\left(\hat{a}^\dagger\hat{a} + \frac{1}{2}\right) + \hbar\omega_{\rm mat}\left(\hat{b}^\dagger\hat{b} + \frac{1}{2}\right) + \hbar g_{\rm SpC}(\hat{a}+\hat{a}^\dagger)(\hat{b}+\hat{b}^\dagger)$
Classical model with coupling term $\propto x_{\rm cav}, x_{\rm mat}$	Alternative model 1<br>$\ddot{x}_{\rm cav} + (\omega_{\rm cav}^2 + 4g_{\rm MoC}^2)x_{\rm cav} - 2g_{\rm MoC}\omega_{\rm mat}x'_{\rm mat} = 0$<br>$\ddot{x}'_{\rm mat} + \omega_{\rm mat}^2 x'_{\rm mat} - 2g_{\rm MoC}\omega_{\rm mat}x_{\rm cav} = 0$	Alternative model 2<br>$\ddot{x}'_{\rm cav} + \omega_{\rm cav}^2 x'_{\rm cav} + 2g_{\rm MoC}\omega_{\rm cav}x_{\rm mat} = 0$<br>$\ddot{x}_{\rm mat} + (\omega_{\rm mat}^2 + 4g_{\rm MoC}^2)x_{\rm mat} + 2g_{\rm MoC}\omega_{\rm cav}x'_{\rm cav} = 0$	SpC model<br>$\ddot{x}_{\rm cav} + \omega_{\rm cav}^2 x_{\rm cav} + 2g_{\rm SpC}\sqrt{\omega_{\rm cav}\omega_{\rm mat}}x_{\rm mat} = 0$<br>$\ddot{x}_{\rm mat} + \omega_{\rm mat}^2 x_{\rm mat} + 2g_{\rm SpC}\sqrt{\omega_{\rm cav}\omega_{\rm mat}}x_{\rm cav} = 0$
Classical model with coupling term $\propto \dot{x}_{\rm cav}, \dot{x}_{\rm mat}$	MoC model<br>$\ddot{x}_{\rm cav} + \omega_{\rm cav}^2 x_{\rm cav} - 2g_{\rm MoC}\dot{x}_{\rm mat} = 0$<br>$\ddot{x}_{\rm mat} + \omega_{\rm mat}^2 x_{\rm mat} + 2g_{\rm MoC}\dot{x}_{\rm cav} = 0$		Alternative model 3<br>$\ddot{x}_{\rm cav} + \left(\omega_{\rm cav}^2 - 4g_{\rm SpC}^2\frac{\omega_{\rm cav}}{\omega_{\rm mat}}\right)x_{\rm cav} - 2g_{\rm SpC}\sqrt{\frac{\omega_{\rm cav}}{\omega_{\rm mat}}}\dot{x}'_{\rm mat} = 0$<br>$\ddot{x}'_{\rm mat} + \omega_{\rm mat}^2 x'_{\rm mat} + 2g_{\rm SpC}\sqrt{\frac{\omega_{\rm cav}}{\omega_{\rm mat}}}\dot{x}_{\rm cav} = 0$
Magnitudes of the oscillators	$x_{\rm cav} = \sqrt{\varepsilon_0 V_{\rm eff}}\mathcal{A}$ $\quad x'_{\rm mat} = \frac{\sqrt{f_{\rm mat}}}{\omega_{\rm mat}}p_{\rm Cou}$<br>$x_{\rm mat} = \frac{d}{\sqrt{f_{\rm mat}}}$ $\quad \left(p_{\rm Cou} = \frac{\dot{d}}{f_{\rm mat}} + \mathcal{A}\right)$	$x_{\rm cav} = \sqrt{\varepsilon_0 V_{\rm eff}}\mathcal{A}$ $\quad x'_{\rm cav} = \frac{\Pi_{\rm Dip}}{\sqrt{\varepsilon_0 V_{\rm eff}}\omega_{\rm cav}}$<br>$x_{\rm mat} = \frac{d}{\sqrt{f_{\rm mat}}}$ $\quad \left(\Pi_{\rm Dip} = \varepsilon_0 V_{\rm eff}\dot{\mathcal{A}} - d\right)$	$x_{\rm cav} = \frac{d_{\rm cav}}{\sqrt{f_{\rm cav}}}$ $\quad x'_{\rm mat} = \frac{\sqrt{f_{\rm mat}}}{\omega_{\rm mat}}p_{\rm mat}$<br>$x_{\rm mat} = \frac{d_{\rm mat}}{\sqrt{f_{\rm mat}}}$ $\quad \left(p_{\rm mat} = \frac{\dot{d}_{\rm mat}}{f_{\rm mat}}\right)$
Frequencies of the hybrid modes	$\omega_\pm = \frac{1}{\sqrt{2}}\sqrt{\omega_{\rm cav}^2 + \omega_{\rm mat}^2 + 4g_{\rm MoC}^2 \pm \sqrt{(\omega_{\rm cav}^2 + \omega_{\rm mat}^2 + 4g_{\rm MoC}^2)^2 - 4\omega_{\rm cav}^2\omega_{\rm mat}^2}}$		$\omega_\pm = \frac{1}{\sqrt{2}}\sqrt{\omega_{\rm cav}^2 + \omega_{\rm mat}^2 \pm \sqrt{(\omega_{\rm cav}^2 - \omega_{\rm mat}^2)^2 + 16g_{\rm SpC}^2\omega_{\rm cav}\omega_{\rm mat}}}$

Table S1: Summary of the correspondences between the classical coupled harmonic oscillator models and the cavity-QED Hamiltonians. We consider the coupling between a dipole (representing, e.g., a molecular excitation) and a dielectric cavity (with transverse electromagnetic modes) or a plasmonic nanocavity (dipole-dipole coupling via Coulomb interactions). The coupling with transverse modes is described in the Coulomb (second column) and dipole (third column) gauges, while the dipole-dipole coupling is described in the same way in both gauges as indicated in the fourth column. The fourth row shows the cavity-QED Hamiltonians that describe each of these situations. The fifth and the sixth rows indicate the corresponding classical harmonic oscillations models: the fifth row corresponds to the models associated with coupling terms proportional to oscillation amplitudes, and the sixth row to models with coupling terms proportional to their time derivatives (with coupling strengths $g_{\rm MoC}$ given by Eq. (S9) and $g_{\rm SpC}$ given by Eq. (S11)). We highlight in green the SpC and MoC models, which are the focus of the main text and for which the bare frequencies $\omega_{\rm cav}$ and $\omega_{\rm mat}$ are considered. With the yellow background, we indicate the alternative models where we use dressed frequencies, which also change the coupling term. The seventh row shows the association between the amplitudes of the oscillators and the physical magnitudes of the system, which differ for each model in the fifth and sixth rows. The last row provides the frequencies of the two hybrid modes for the two different types of interaction. To ease comparison, we write both the cavity-QED Hamiltonians and coupled harmonic oscillator models in terms of $g_{\rm SpC}$ and $g_{\rm MoC}$.

in a dielectric (Fabry-Pérot) cavity, and the dipole-dipole coupling due to the Coulomb interaction. The models describing the first type of interaction (second and third columns) depend on the chosen gauge (Coulomb or dipole) [4], but all of them result in identical eigenfrequencies and other physical magnitudes (the latter require to take into account the specific connection of the classical oscillation amplitudes and quantum operators with, e.g., the electric field depends on the model). All the models in the fourth column describing dipole-dipole Coulomb coupling are also equivalent to each other. On the other hand, the models in the fourth column are not equivalent to those in the second and third columns.

Table S1 shows that if the classical equations depend directly on the bare (non-dressed) frequencies of the uncoupled oscillators $\omega_{\rm cav}$ and $\omega_{\rm mat}$, the description of the interaction between transverse cavity modes and matter excitations requires a coupling term proportional to the time derivatives of the oscillation amplitudes (MoC model, equivalent to cavity-QED Hamiltonians with diamagnetic term). In contrast, the coupling term associated with dipole-dipole interactions is proportional to the oscillation amplitudes where frequencies are not dressed (SpC model, equivalent to cavity-QED Hamiltonians without diamagnetic term). These classical models are analyzed in the main text and Supplementary Sec. S1 and highlighted in green. On the other hand, as discussed in Supplementary Sec. S2, each type of interaction can also be modeled with alternative models where the type of coupling term is modified from proportional to the oscillation amplitudes to proportional to their time derivatives, or vice versa. We highlight these models in Table S1 by the yellow squares. In these alternative models, dressing or renormalization of one of the oscillator frequencies is needed to maintain their equivalence with their corresponding cavity-QED Hamiltonians. Further, the alternative classical models also require the modification of the physical magnitudes that each oscillator represents. However, if the transformation of oscillation amplitudes and frequencies is done appropriately, all models describing the coupling between transverse fields and dipoles (second and third columns) yield identical results, and the same happens for dipole-dipole interactions (fourth column).

S5 Linearized Model

We show in this section that, for $g < 0.1\omega_{\rm mat}$ (i.e., before the onset of ultrastrong coupling according to the standard definition of this regime), it is possible to reduce both the MoC and the SpC model to the same simplified linearized model by considering that the eigenfrequencies $\omega_\pm$ do not differ too strongly from the bare frequencies ω_α (α = 'cav' or α = 'mat').

Using the approximation $\omega_\alpha + \omega \approx 2\omega \approx 2\omega_\alpha$, the frequency-domain equations of both the SpC and MoC models become linear in ω:

$$(\omega_{\rm cav} - \omega)x_{\rm cav} + g_{\rm lin}x_{\rm mat} = 0 \tag{S36a}$$

$$(\omega_{\rm mat} - \omega)x_{\rm mat} + g^*_{\rm lin}x_{\rm cav} = 0, \tag{S36b}$$

with $g_{\rm lin} = g_{\rm SpC} = ig_{\rm MoC}$. The resulting eigenfrequencies are

$$\omega_{\pm,\rm lin} = \frac{\omega_{\rm cav} + \omega_{\rm mat} \pm \sqrt{(\omega_{\rm cav} - \omega_{\rm mat})^2 + 4|g_{\rm lin}|^2}}{2}. \tag{S37}$$

We compare in Fig. S1 the results of this model (black dots) to those obtained with the MoC (red dashed lines) and SpC (blue solid lines) models. The results are obtained with Eq. (S37), and Eqs.

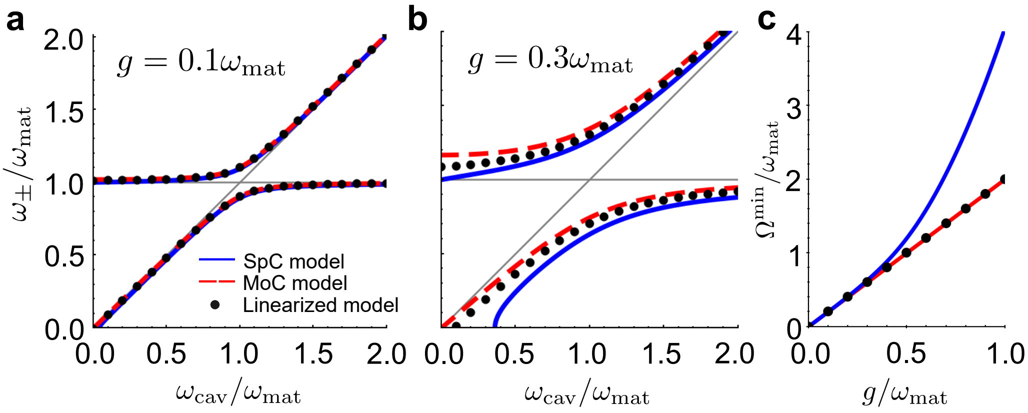

Figure S1: Comparison of the Spring Coupling (SpC), Momentum Coupling (MoC), and linearized models. a) Eigenfrequencies $\omega_{\pm}$ of the hybrid states calculated from the bare values ω_{cav} and ω_{mat}, with ω_{mat} fixed and $\omega_{\text{cav}}/\omega_{\text{mat}}$ changing. $\omega_{\pm}$ obtained from the SpC model (blue solid line, corresponding to Eq. (10) in the main text), MoC model (red dashed line, Eq. (13) in the main text) and the approximate linearized model (black dots, Eq. (S37)), for coupling strength $g = g_{\text{SpC}} = g_{\text{MoC}} = g_{\text{lin}} = 0.1\,\omega_{\text{mat}}$. The thin gray lines correspond to the bare cavity frequency ω_{cav} and the bare frequency of the matter excitation, ω_{mat}. b) Same as panel (a), for coupling strength $g = g_{\text{SpC}} = g_{\text{MoC}} = g_{\text{lin}} = 0.3\,\omega_{\text{mat}}$. c) Minimum splitting between the hybrid modes $\Omega^{\text{min}} = \omega_{+} - \omega_{-}$, as a function of the coupling strength g for the SpC model (blue solid line), the MoC model (red solid line) and the linearized model (black dots). All frequencies are normalized with respect to the fixed frequency of the matter excitation ω_{mat}, so that the results do not depend on the particular value of ω_{mat}, only on the $\omega_{\text{cav}}/\omega_{\text{mat}}$ ratio or g/ω_{cav} ratio . The MoC and SpC results are the same as in Fig. 2 of the main text.

(13) and (10) of the main text, respectively. Fig. S1a shows that, for $g = 0.1\omega_{\text{mat}}$ (as in Sec. 2.4 of the main text, we use g to refer to g_{SpC}, g_{MoC} and/or g_{lin} in discussions that are valid for more than one model), the three models indeed result in very similar eigenvalues for all values of $\omega_{\text{cav}}/\omega_{\text{mat}}$. However, this is not the case for $g = 0.3\omega_{\text{mat}}$ (Fig. S1b), where the eigenfrequencies of the linearized model are typically in between those of the SpC and MoC models. Notably, the linearized model does not present any mode in a forbidden energy band that is half as wide as in the MoC model (while the SpC model did not present such a forbidden band). Similarly, the linearized model does not present any lower-energy mode, (i.e., negative $\omega_{-,\text{lin}}$, for $\frac{\omega_{\text{cav}}}{\omega_{\text{mat}}} < \left(\frac{g_{\text{lin}}}{\omega_{\text{mat}}}\right)^2$); in contrast, the corresponding condition for the SpC model is $\frac{\omega_{\text{cav}}}{\omega_{\text{mat}}} < \left(\frac{2g_{\text{SpC}}}{\omega_{\text{mat}}}\right)^2$ (where $\omega_{-,\text{SpC}}$ is imaginary) and the MoC model always presents a lower-energy mode. On the other hand, the splitting at zero detuning is equal to $\Omega = 2g$ in both the linearized and MoC models (but with different values of $\omega_{\pm}$ for each of them), which is the minimum splitting in these two models. In contrast, the minimum splitting scales non-linearly with g for the SpC model, and is larger than for the MoC or linearized modes. These results are illustrated in Fig. S1(c), which shows the dependence of the normalized minimum splitting $\Omega^{\text{min}}/\omega_{\text{mat}}$ on the normalized coupling strength g/ω_{mat}.

S6 Evolution of the eigenvalues for a different choice of coupling strength

In Sec. 2.4 of the main text (as well as in Sec. S5), we consider that the coupling strengths g_{MoC} and g_{SpC} do not depend on the resonant frequency of the cavity ω_{cav}. This choice is consistent with

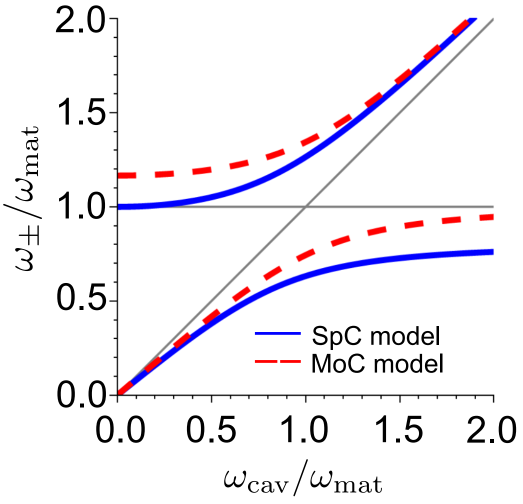

Figure S2: Comparison of the Spring Coupling (SpC) and Momentum Coupling (MoC) models for a different choice of the coupling strength than in the main text. Eigenfrequencies $\omega_\pm$ obtained as a function of $\omega_{\rm cav}$, obtained from the SpC model (blue solid line, corresponding to Eq. (10) in the main text) and MoC model (red dashed line, Eq. (13) in the main text) for coupling strength $g_{\rm SpC} = 0.3\sqrt{\omega_{\rm cav}\omega_{\rm mat}}$ and $g_{\rm MoC} = 0.3\,\omega_{\rm mat}$, respectively. The thin gray lines correspond to the bare cavity frequency $\omega_{\rm cav}$ and the bare frequency of the matter excitation, $\omega_{\rm mat}$. All frequencies are normalized with respect to the fixed frequency of the matter excitation $\omega_{\rm mat}$ ($\hbar\omega_{\rm mat} = 0.1$ eV), and the MoC results are the same as in Fig. 2 of the main text.

the results obtained in Secs. 3.1 and 3.3 of the main text, which used the MoC model to describe the coupling of a molecular emitter or an ensemble of molecular emitters with the transverse fields of an electromagnetic mode of a dielectric (Fabry-Pérot) cavity. On the other hand, when describing the Coulomb coupling within the SpC model in Sec. 3.2, the dependence of the coupling strength $g_{\rm SpC}$ on $\omega_{\rm cav}$ can vary with the details of each particular configuration. To exemplify the consequences of such details, we consider in this section a different dependence of $g_{\rm SpC}$ on $\omega_{\rm cav}$ than in the main text.

We analyze again the Coulomb coupling between metallic spherical particles of radius $R_{\rm cav}$ and a quantum emitter, described with the SpC model as in Sec. 3.2 of the main text. However, we now change the plasma frequency of the metal, which modifies the dipole moment $f_{\rm cav} = 4\pi\varepsilon_0 R_{\rm cav}^3\omega_{\rm cav}^2$ so that, according to Eq. (28) in the main text, the coupling strength scales as $g_{\rm SpC} \propto \sqrt{\omega_{\rm cav}}$ (assuming a constant $f_{\rm mat}$).

We then plot in Fig. S2 the results obtained within the MoC model for $\hbar\omega_{\rm mat} = 0.1$ eV, $g_{\rm MoC} = 0.3\,\omega_{\rm mat}$ (red dashed line, same as in the main text) and within the SpC model for coupling strength $g_{\rm SpC} = 0.3\omega_{\rm mat}\sqrt{\omega_{\rm cav}/\omega_{\rm mat}} = 0.3\sqrt{\omega_{\rm cav}\omega_{\rm mat}}$ (blue solid line), as $\omega_{\rm cav}$ is changed. With this choice, $g_{\rm MoC} = g_{\rm SpC}$ under resonant conditions ($\omega_{\rm cav} = \omega_{\rm mat}$). We find that, for this scaling of $g_{\rm SpC}$, the SpC model results in two (real valued) eigenfrequencies for all values of $\omega_{\rm cav}$, as well as in the opening of a Reststrahlen band. Interestingly, however, this band appears for energies smaller than $\omega_{\rm mat}$, contrary to the result for the MoC model.

S7 Transformation from individual to collective oscillators in the description of homogeneous materials in Fabry-Pérot cavities

In Sec. 3.3 of the main article, we analyze how classical models of harmonic oscillators describe an ensemble of $N_{\rm dip}$ molecules (or a homogeneous material) inside a Fabry-Pérot cavity. Each molecular

emitter couples with all the other molecular emitters and also with the transverse modes of the cavity, and all these interactions can be modeled through Eq. (33) in the main text. In this supplementary section, we show in more detail how to describe this system by considering the coupling of each Fabry-Pérot mode with a single collective mode of matter oscillators. Specifically, here we demonstrate how to transform Eq. (33) in Sec. 3.3 of the main text, written in terms of harmonic oscillators of individual molecular excitations, into Eq. (34), which considers collective modes. This derivation can be generalized to other cavities by following the same procedure but using the spatial distribution of the transverse electric field of the corresponding cavity modes.

We assume that the Fabry-Pérot cavity contains perfect mirrors in the planes $z = 0$ and $z = L_{\text{cav}}$ (L_{cav} is the thickness of the cavity), so that the cavity has transverse electric (TE) modes with field distribution[c]

$$\Xi_{n\mathbf{k}_{\parallel}}(\mathbf{r}) = \sin\left(\frac{n\pi z}{L_{\text{cav}}}\right) e^{i\mathbf{k}_{\parallel}\cdot\mathbf{r}_{\parallel}}. \tag{S38}$$

The integer n indexes all modes of the cavity and the wavevector in the parallel direction $\mathbf{k}_{\parallel}$ is any two-dimensional vector (we consider a discrete set of $\mathbf{k}_{\parallel}$ by assuming that the cavity has long but finite size in the lateral dimensions and using Born-von Karman periodic boundary conditions for Eq. (S38)). We further assume that the direction of the transition dipole moments of the molecules is the same as that of the electric field of the mode (parallel to the mirror planes). As a consequence, the coupling strength between each molecular emitter placed in the position $\mathbf{r}_i = (\mathbf{r}_{\parallel,i}, z_i)$ and the $n\mathbf{k}_{\parallel}$ Fabry-Pérot mode is calculated with the expression $g_{\text{MoC}}^{(n\mathbf{k}_{\parallel},i)} = \frac{1}{2}\sqrt{\frac{f_{\text{dip}}}{\varepsilon_0 V_{\text{eff}}}}\Xi_{n\mathbf{k}_{\parallel}}(\mathbf{r}_i)$ (see discussion of Supplementary Sec. S1 and Eq. (S9)). By introducing the field distribution of Eq. (S38) in the expression of the coupling strength explicitly, the equations of motion of the system (Eq. (33) in the main text) become

$$\ddot{x}_{\text{cav},n\mathbf{k}_{\parallel}} + \omega_{\text{cav},n\mathbf{k}_{\parallel}}^2 x_{\text{cav},n\mathbf{k}_{\parallel}} - \sum_i \sqrt{\frac{f_{\text{dip}}}{\varepsilon_0 V_{\text{eff}}}} \sin\left(\frac{n\pi z_i}{L_{\text{cav}}}\right) e^{-i\mathbf{k}_{\parallel}\cdot\mathbf{r}_{\parallel i}} \dot{x}_{\text{dip},i} = 0, \tag{S39a}$$

$$\ddot{x}_{\text{dip},i} + \omega_{\text{dip}}^2 x_{\text{dip},i} + \sum_{n',\mathbf{k}'_{\parallel}} \sqrt{\frac{f_{\text{dip}}}{\varepsilon_0 V_{\text{eff}}}} \sin\left(\frac{n'\pi z_i}{L_{\text{cav}}}\right) e^{i\mathbf{k}'_{\parallel}\cdot\mathbf{r}_{\parallel i}} \dot{x}_{\text{cav},n'\mathbf{k}'_{\parallel}} + \sum_{j\neq i} 2\omega_{\text{dip}} g_{\text{SpC}}^{(i,j)} x_{\text{dip},j} = 0. \tag{S39b}$$

In Eq. (S39a), we already observe that the oscillator $x_{\text{cav},n\mathbf{k}_{\parallel}}$ of the $n\mathbf{k}_{\parallel}$ cavity mode is coupled to a collective matter operator. By defining the collective oscillator of the $n\mathbf{k}_{\parallel}$ matter mode as

$$x_{\text{mat},n\mathbf{k}_{\parallel}} = \frac{1}{\sqrt{N_{\text{eff}}}} \sum_i e^{-i\mathbf{k}_{\parallel}\cdot\mathbf{r}_{\parallel i}} \sin\left(\frac{n\pi z_i}{L_{\text{cav}}}\right) x_{\text{dip},i}, \tag{S40}$$

Equation (S39a) becomes

$$\ddot{x}_{\text{cav},n\mathbf{k}_{\parallel}} + \omega_{\text{cav},n\mathbf{k}_{\parallel}}^2 x_{\text{cav},n\mathbf{k}_{\parallel}} - 2\sqrt{N_{\text{eff}}} g_{\text{MoC}}^{\text{max}} \dot{x}_{\text{mat},n\mathbf{k}_{\parallel}} = 0. \tag{S41}$$

where $g_{\text{MoC}}^{\text{max}} = \frac{1}{2}\sqrt{\frac{f_{\text{dip}}}{\varepsilon_0 V_{\text{eff}}}}$ is the maximum achievable coupling strength between a single molecular emitter and a cavity mode in this system, found for molecules placed in the antinodes of the mode.

[c]To simplify the discussion, here we show explicitly the transformation under the field distribution of TE modes. Fabry-Pérot cavities also have transverse magnetic (TM) modes, and all the transformations are equivalent after substituting the field distribution of these modes into Eq. (S38), but additional care needs to be taken to account for the position dependence of the polarization direction of the cavity fields.

$N_{\text{eff}} = \sum_i \left|\Xi_{n\mathbf{k}_\parallel}(\mathbf{r}_i)\right|^2$ is the effective number of molecular emitters that couple with the cavity mode, whose exact relation with the total number of molecular emitters N_{dip} depends on the system and the spatial distribution of the modes. By performing the sum $N_{\text{eff}} = \sum_i \left|\Xi_{n\mathbf{k}_\parallel}(\mathbf{r}_i)\right|^2 = \sum_i \left|\sin\left(\frac{n\pi z_i}{L_{\text{cav}}}\right)\right|^2$ with the specific field distribution of Fabry-Pérot cavity modes, we obtain $N_{\text{eff}} = N_{\text{dip}}/2$ for this cavity. We observe in Eq. (S41) that the coupling strength between the cavity mode and the collective oscillator mode increases as $g_{\text{MoC}}^{\max}\sqrt{N_{\text{eff}}}$. This scaling of the coupling strength (together with the scaling as $1/\sqrt{N_{\text{eff}}}$ of the collective oscillator in Eq. (S40)) is the same as in the quantum Dicke model [5], further confirming that the classical oscillator models are consistent with cavity-QED descriptions.

The next step is to transform Eq. (S39b), which requires considering N_{dip} equations simultaneously, one per molecular emitter at position $\mathbf{r}_i$. To do the transformation, we multiply Eq. (S39b) by $\frac{1}{\sqrt{N_{\text{eff}}}}\sin\left(\frac{n\pi z_i}{L_{\text{cav}}}\right)e^{-i\mathbf{k}_\parallel\cdot\mathbf{r}_{\parallel i}}$ for each i molecular emitter and sum the N_{dip} resulting terms. With this procedure, and using Eq. (S40), the transformation of the first two terms is straightforward as

$$\frac{1}{\sqrt{N_{\text{eff}}}}\sum_i \sin\left(\frac{n\pi z_i}{L_{\text{cav}}}\right)e^{-i\mathbf{k}_\parallel\cdot\mathbf{r}_{\parallel i}}(\ddot{x}_{\text{dip},i} + \omega_{\text{dip}}^2 x_{\text{dip},i}) = \ddot{x}_{\text{mat},n\mathbf{k}_\parallel} + \omega_{\text{dip}}^2 x_{\text{mat},n\mathbf{k}_\parallel}. \tag{S42}$$

Repeating the procedure with the third term of Eq. (S39b), we obtain

$$\begin{aligned}&\frac{2}{\sqrt{N_{\text{eff}}}}g_{\text{MoC}}^{\max}\sum_{n',\mathbf{k}'_\parallel}\dot{x}_{\text{cav},n'\mathbf{k}'_\parallel}\sum_i \sin\left(\frac{n'\pi z_i}{L_{\text{cav}}}\right)\sin\left(\frac{n\pi z_i}{L_{\text{cav}}}\right)e^{i(\mathbf{k}_\parallel-\mathbf{k}'_\parallel)\cdot\mathbf{r}_\parallel}\\ &= \frac{2}{\sqrt{N_{\text{eff}}}}g_{\text{MoC}}^{\max}\sum_{n',\mathbf{k}'_\parallel}\dot{x}_{\text{cav},n\mathbf{k}_\parallel}N_{\text{eff}}\delta_{n,n'}\delta_{\mathbf{k}_\parallel,\mathbf{k}'_\parallel} = 2g_{\text{MoC}}^{\max}\sqrt{N_{\text{eff}}}\dot{x}_{\text{cav},n\mathbf{k}_\parallel}.\end{aligned} \tag{S43}$$

Equation (S43) shows that, although each molecular emitter couples with all Fabry-Pérot modes of different $\mathbf{k}_\parallel$, the collective matter oscillator of amplitude $x_{\text{mat},n\mathbf{k}_\parallel}$, described by the indexes n and $\mathbf{k}_\parallel$, only couples with the cavity mode of same indexes due to the orthogonality of all these modes.

Last, we transform the fourth term of Eq. (S39b), which involves molecule-molecule interactions. To perform this transformation, we consider the SpC coupling strength between molecular emitters as given by Eq. (S11) explicitly, which leads to

$$\begin{aligned}&\frac{1}{\sqrt{N_{\text{eff}}}}\sum_i\sum_{j\neq i}2\omega_{\text{dip}}g_{\text{SpC}}^{(i,j)}\sin\left(\frac{n\pi z_i}{L_{\text{cav}}}\right)e^{-i\mathbf{k}_\parallel\cdot\mathbf{r}_{\parallel i}}x_{\text{mat},j}\\ &= \frac{1}{\sqrt{N_{\text{eff}}}}\sum_j 2\omega_{\text{dip}}x_{\text{mat},j}e^{-i\mathbf{k}_\parallel\cdot\mathbf{r}_{\parallel j}}\sum_{i\neq j}g_{\text{SpC}}^{(i,j)}\sin\left(\frac{n\pi z_i}{L_{\text{cav}}}\right)e^{-i\mathbf{k}_\parallel\cdot(\mathbf{r}_{\parallel i}-\mathbf{r}_{\parallel j})}\\ &= \frac{1}{\sqrt{N_{\text{eff}}}}\sum_j 2\omega_{\text{dip}}x_{\text{mat},j}e^{-i\mathbf{k}_\parallel\cdot\mathbf{r}_{\parallel j}}\sum_{i\neq j}\frac{1}{2}\frac{f_{\text{dip}}e^{-i\mathbf{k}_\parallel\cdot(\mathbf{r}_{\parallel i}-\mathbf{r}_{\parallel j})}}{4\pi\varepsilon_0|\mathbf{r}_i-\mathbf{r}_j|^3\omega_{\text{dip}}}[1-3(\mathbf{n_d}\cdot\mathbf{n}_{\mathbf{r}ij})]\sin\left(\frac{n\pi z_i}{L_{\text{cav}}}\right)\\ &\approx \frac{1}{\sqrt{N_{\text{eff}}}}\sum_j 2\omega_{\text{dip}}x_{\text{mat},j}e^{-i\mathbf{k}_\parallel\cdot\mathbf{r}_{\parallel j}}\sin\left(\frac{n\pi z_j}{L_{\text{cav}}}\right)\underbrace{\sum_{i\neq j}\frac{1}{2}\frac{f_{\text{dip}}e^{-i\mathbf{k}_\parallel\cdot(\mathbf{r}_{\parallel i}-\mathbf{r}_{\parallel j})}}{4\pi\varepsilon_0|\mathbf{r}_i-\mathbf{r}_j|^3\omega_{\text{dip}}}[1-3(\mathbf{n_d}\cdot\mathbf{n}_{\mathbf{r}ij})]}_{g_{\text{shift}}^{(n\mathbf{k}_\parallel)}}\\ &= 2\omega_{\text{dip}}g_{\text{shift}}^{(n\mathbf{k}_\parallel)}x_{\text{mat},n\mathbf{k}_\parallel}.\end{aligned} \tag{S44}$$

In the fourth line in Eq. (S44), we have considered that the dipole-dipole coupling strength between different molecular emitters, which depends on their distance as $|\mathbf{r}_i - \mathbf{r}_j|^{-3}$, decays much faster over z than the term $\sin(n\pi z/L_{\text{cav}})$ changes (unless n is so large that it has very fast oscillations, which we do

not consider here). Due to this fast decay, we have checked numerically that the term $\sin(n\pi z_i/L_{\rm cav})$ can be taken outside the sum over the molecular emitters i as a constant of value $\sin(n\pi z_j/L_{\rm cav})$, i.e. where only the emitter j is involved. The sum over the variable i in Eq. (S44) can be then performed numerically to obtain the collective molecule-molecule coupling strength $g_{\rm shift}^{(n\mathbf{k}_\parallel)}$.

Therefore, by gathering all transformed terms in Eqs. (S42), (S43) and (S44), and using Eq. (S40), Eq. (S39b) becomes

$$\ddot{x}_{{\rm mat},n\mathbf{k}_\parallel} + (\omega_{\rm dip}^2 + 2\omega_{\rm dip} g_{\rm shift}^{(n\mathbf{k}_\parallel)}) x_{{\rm mat},n\mathbf{k}_\parallel} + 2 g_{\rm MoC}^{\rm max}\sqrt{N_{\rm eff}}\dot{x}_{{\rm cav},n\mathbf{k}_\parallel} = 0. \tag{S45}$$

Equations (S41) and (S45) correspond to Eqs. (34a) and (34b) in the main article. Importantly, the derivation carried out in this section shows two important features of light-matter coupling in this system: i) although each $n\mathbf{k}_\parallel$ cavity mode is coupled to all individual molecular emitters, it is only coupled to the $n\mathbf{k}_\parallel$ collective mode due to the orthogonality of the modes, and ii) the only consequence of the molecule-molecule coupling for the interaction between the $n\mathbf{k}_\parallel$ cavity and collective matter modes is to shift the bare frequency of the matter oscillator from $\omega_{\rm dip}$ to $\sqrt{\omega_{\rm dip}^2 + 2\omega_{\rm dip} g_{\rm shift}^{(n\mathbf{k}_\parallel)}}$ [6].

S8 Reststrahlen band

In the main text, we have shown that, if we impose that the resonant cavity mode and matter excitation frequencies in the coupled harmonic oscillator model are the bare ones without any dressing, then the Reststrahlen band is only correctly recovered when we use the MoC model, i.e., the coupling term is proportional to the time derivative of the amplitude of the oscillators. However, according to Sec. S2 and Table S1, if we relax this condition and dress the cavity mode or matter excitation, we find alternative classical harmonic oscillator models that are equivalent to the MoC model but that use a coupling term proportional to the oscillation amplitude and the appropriate dressing. Here, we apply this finding to demonstrate how to reproduce the Reststrahlen band with a coupling term proportional to the oscillator amplitudes. The results are equivalent to those obtained using the Hopfield Hamiltonian [7]. To obtain the Reststrahlen band, we first follow the approach in Ref. [8] to obtain the bulk dispersion of a phononic material directly from the response of an infinite material (an equivalent demonstration could be performed by connecting the dispersion of a Fabry-Pérot cavity with the bulk dispersion as in Sec. 3.3)

In the previous work, the authors considered a phononic material with a permittivity given by Eq. (38) in the main text and derived the system of equations

$$\begin{pmatrix} \omega^2 - \omega_{\rm TO}^2 & \omega\omega_{\rm p} \\ \omega\omega_{\rm p} & \omega^2 - \omega_k^2 \end{pmatrix} \begin{pmatrix} i j_{\rm latt}/(q_{\rm i}) \\ \sqrt{\varepsilon_0\varepsilon_\infty}|\mathbf{E}(k)_{\rm inc}| \end{pmatrix} = 0 \tag{S46}$$

where $|\mathbf{E}(k)_{\rm inc}|$ is the amplitude of the incident electric field, $q_{\rm i}$ the (positive) charge of the lattice ions, ε_∞ the high-frequency permittivity of the material, ω the frequency of the bulk dispersion modes, $\omega_k = \frac{ck_0}{\sqrt{\varepsilon_\infty}}$ the frequency of a free photon of vacuum wavevector k_0 propagating in a medium of permittivity ε_∞ and k the wavevector in this material. $\omega_{\rm p} = \sqrt{\omega_{\rm LO}^2 - \omega_{\rm TO}^2}$ is the parameter that controls the coupling strength in this model and $\omega_{\rm LO}$ and $\omega_{\rm TO}$ the frequency of the longitudinal and transverse optical phonon modes, respectively. According to the discussion in Sec. 3.3 of the main text, $\omega_{\rm TO}$ is here the bare frequency of the material. As a difference to the previous work, we neglect losses, and we have written the equations as a function of the normalized microscopic

current $j_{\rm latt}$, which depends on the normalized relative displacement $x_{\rm latt}$ of the atoms in the atomic lattice [8] through $j_{\rm latt} = -iq_i\omega x_{\rm latt}$ ($x_{\rm latt}$ correspond to the normalized displacement of the atoms that are positively charged from the atoms that are mostly negatively charged). We can then identify $x_k = \sqrt{\varepsilon_0\varepsilon_\infty}|\mathbf{E}(k)_{\rm inc}|$ (the amplitude of the oscillator associated with the field of wavevector k) and $x_{\rm mat} = -j_{\rm latt}/(q_{\rm i})$ (associated with the matter excitation) and rewrite Eq. (S46) as

$$-i\omega^2 x_{\rm mat} + i\omega_{\rm TO}^2 x_{\rm mat} + \omega\omega_{\rm p} x_k = 0 \tag{S47a}$$

$$\omega^2 x_k - \omega_k^2 x_k - i\omega\omega_{\rm p} x_{\rm mat} = 0, \tag{S47b}$$

which can be rewritten in the time domain to obtain

$$i\ddot{x}_{\rm mat} + i\omega_{\rm TO}^2 x_{\rm mat} + i\omega_{\rm p}\dot{x}_k = 0 \tag{S48a}$$

$$-\ddot{x}_k - \omega_k^2 x_k + \omega_{\rm p}\dot{x}_{\rm mat} = 0, \tag{S48b}$$

or, equivalently,

$$\ddot{x}_k + \omega_k^2 x_k - 2G_{\rm MoC}\dot{x}_{\rm mat} = 0, \tag{S49a}$$

$$\ddot{x}_{\rm mat} + \omega_{\rm mat}^2 x_{\rm mat} + 2G_{\rm MoC}\dot{x}_k = 0. \tag{S49b}$$

where $\omega_{\rm mat} = \omega_{\rm TO}$ and $G_{\rm MoC} = \frac{\omega_{\rm p}}{2} = \frac{\sqrt{\omega_{\rm LO}^2 - \omega_{\rm TO}^2}}{2}$, so that we recover Eq. (35) in the main text. This equivalence indicates that the result obtained here for an infinite phononic material coincides with those obtained in Sec. 3.3 of the main text, where we focused on the eigenmodes of the Fabry-Pérot cavity. Following the discussion in that section, this further confirms that Eq. (S49) describes the bulk dispersion within the MoC model.

On the other hand, Table S1 indicates that an equivalent dispersion can be obtained with the following coupled harmonic oscillator model (alternative model 1):

$$\ddot{x}_k + (\omega_k^2 + 4G_{\rm MoC}^2)x_k - 2G_{\rm MoC}\omega_{\rm mat}x'_{\rm mat} = 0, \tag{S50a}$$

$$\ddot{x}'_{\rm mat} + \omega_{\rm mat}^2 x'_{\rm mat} - 2G_{\rm MoC}\omega_{\rm mat}x_k = 0, \tag{S50b}$$

where the coupling term is proportional to the amplitude of the oscillators. We can rewrite this equation as

$$\ddot{x}_k + \left(\omega_k^{\rm A1}\right)^2 x_k + 2G^{\rm A1}\sqrt{\omega_{\rm mat}\omega_k^{\rm A1}}x'_{\rm mat} = 0, \tag{S51a}$$

$$\ddot{x}'_{\rm mat} + \omega_{\rm mat}^2 x'_{\rm mat} + 2G^{\rm A1}\sqrt{\omega_{\rm mat}\omega_k^{\rm A1}}x_k = 0, \tag{S51b}$$

$$\omega_k^{\rm A1} = \sqrt{\omega_k^2 + 4G_{\rm MoC}^2}, \tag{S51c}$$

$$G^{\rm A1} = -G_{\rm MoC}\frac{\omega_{\rm mat}}{\sqrt{\omega_{\rm mat}\omega_k^{\rm A1}}} = -G_{\rm MoC}\sqrt{\frac{\omega_{\rm mat}}{\sqrt{\omega_k^2 + 4G_{\rm MoC}^2}}}. \tag{S51d}$$

Crucially, the first two equations are formally equivalent to the SpC model, except that in this case, $\omega_k^{\rm A1}$ is a dressed frequency and the coupling strengths $G^{\rm A1}$ has been changed, as given by the last two equations (the superscript 'A1' refers to alternative model 1).

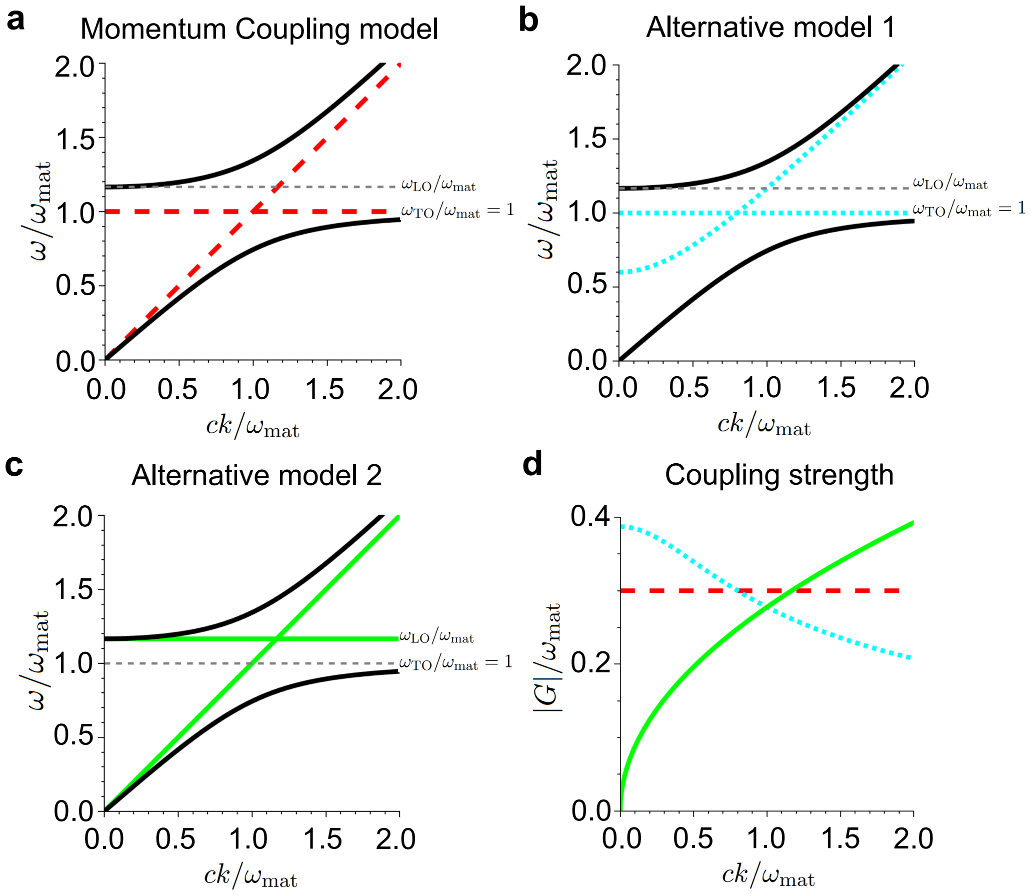

Figure S3: Bulk dispersion and opening of the Reststrahlen band according to different models. (a) Bulk dispersion (black line) and uncoupled frequencies (bare photon frequency ω_k, diagonal red dashed line; transverse optical frequency ω_{TO}, horizontal red dashed line) according to the Momentum Coupling (MoC) model. The horizontal dashed line corresponds to the longitudinal optical phonon frequency. (b) Bulk dispersion (black line) and uncoupled frequencies (dressed photon frequency ω_k^{A1}, diagonal-like cyan short-dashed line; bare transverse optical phonon frequency ω_{TO}, horizontal cyan short-dashed line) according to the alternative model 1. The horizontal dashed line corresponds to the longitudinal optical phonon frequency. (c) Bulk dispersion (black line) and uncoupled frequencies (bare photon frequency ω_k, diagonal green solid line; longitudinal optical phonon frequency ω_{LO}, horizontal green solid line) according to alternative model 2. The horizontal dashed line corresponds to the transverse optical phonon frequency. (d) Coupling strength as a function of wavevector according to the MoC model (G_{MoC}, red dashed line), the alternative model 1 ($|G^{\mathrm{A1}}|$, cyan short-dashed line) and the alternative model 2 (G^{A2}, green solid line). In all panels, the bulk dispersion is the same (black lines, corresponding to the one obtained for $G_{\mathrm{MoC}} = 0.3\omega_{\mathrm{mat}}$), all frequencies and the coupling strength are normalized by $\omega_{\mathrm{mat}} = \omega_{\mathrm{TO}}$ and the results are plotted as a function of the normalized wavevector ck/ω_{mat}.

Proceeding in the same manner but for the alternative model 2 in Table 1, we obtain a second set of coupled harmonic oscillations that also give the same dispersion.

$$\ddot{x}'_k + \omega_k^2 x'_k + 2G^{\mathrm{A2}}\sqrt{\omega_{\mathrm{mat}}^{\mathrm{A2}}\omega_k}x_{\mathrm{mat}} = 0, \tag{S52a}$$

$$\ddot{x}_{\mathrm{mat}} + \left(\omega_{\mathrm{mat}}^{\mathrm{A2}}\right)^2 x_{\mathrm{mat}} + 2G^{\mathrm{A2}}\sqrt{\omega_{\mathrm{mat}}^{\mathrm{A2}}\omega_k}x'_k = 0, \tag{S52b}$$

$$\omega_{\mathrm{mat}}^{\mathrm{A2}} = \sqrt{\omega_{\mathrm{mat}}^2 + 4G_{\mathrm{MoC}}^2} = \sqrt{\omega_{\mathrm{TO}}^2 + 4G_{\mathrm{MoC}}^2} = \omega_{\mathrm{LO}}, \tag{S52c}$$

$$G^{\mathrm{A2}} = G_{\mathrm{MoC}}\frac{\omega_k}{\sqrt{\omega_{\mathrm{mat}}^{\mathrm{A2}}\omega_k}} = G_{\mathrm{MoC}}\sqrt{\frac{\omega_k}{\sqrt{\omega_{\mathrm{mat}}^2 + 4G_{\mathrm{MoC}}^2}}}, \tag{S52d}$$

where, in this case, the dressed frequency is that of the matter excitation $\omega_{\mathrm{mat}}^{\mathrm{A2}}$.

The different coupled harmonic oscillator models are compared in Fig. S3. The bulk dispersion obtained for $G_{\mathrm{MoC}} = 0.3\omega_{\mathrm{mat}} = 0.3\omega_{\mathrm{TO}}$ (and corresponding values of G^{A1} and G^{A2}) is shown in panels (a-c) by the black lines. As expected, the three models give identical results. The red dashed lines in Fig. S3(a) show the frequencies of the uncoupled modes (i.e. the frequencies that are obtained if the coupling is ignored) of the MoC model given by Eq. (S49), corresponding to the frequency of the transverse optical mode, ω_{TO}, and of the free photons in the material of permittivity ε_∞, ω_k. Figure S3(b) shows the corresponding result for the alternative model 1, with the uncoupled modes (cyan short-dashed line) being in this is case the TO photon at frequency ω_{TO} and the dressed photon at frequency ω_k^{A1}. Last, the uncoupled frequencies of the alternative model 2 are indicated by the solid green line in Fig. S3(c) and correspond to the LO phonon frequency ω_{LO} and of the free photons ω_k. The coupling strength that need to be used in each of this models to reproduce the same bulk dispersion is shown in Fig. S3(d) (red dashed line corresponds to the coupling strength in the MoC model, $G_{\mathrm{MoC}} = 0.3\omega_{\mathrm{mat}}$; the cyan short-dashed line corresponds to the coupling strength in the alternative model 1, G^{A1}; the green solid line to the coupling strength in alternative model 2, G^{A2}). These results thus stress that the same bulk dispersion can be obtained using different classical coupled harmonic oscillator models.

Last, we emphasize that the possibility of obtaining the same dispersion with both the MoC model (Eq. (S49)) and the second alternative model (Eq. (S52)) indicates that the bulk dispersion can be obtained with classical coupled harmonic oscillator models that use a coupling term that can be proportional to either the oscillator amplitude (alternative model 2) and to its derivative (MoC model). These two models offer a very different picture of the opening of the Reststrahlen band (we do not discuss here the first alternative model because it does not have a simple physical interpretation):

- According to the second alternative model (Eq. (S52)), the dressed matter excitation in the coupled equations corresponds to the longitudinal optical phonon frequency, $\omega_{\mathrm{mat}}^{\mathrm{A2}} = \sqrt{\omega_{\mathrm{TO}}^2 + 4G_{\mathrm{MoC}}^2} = \omega_{\mathrm{LO}}$, the renormalized coupling strength G^{A2} becomes zero for photons of energy (or momentum) tending to zero, and $\left(G^{\mathrm{A2}}\right)^2$ scales linearly with photon energy. Thus, in this picture, i) the square of the coupling term is proportional to the energy of the photons, ii) the longitudinal optical phonon appears as the resonant matter excitation in the harmonic oscillator equations, and can be interpreted as the dressed matter excitation of the 'bare' transverse optical phonon, iii) the (dressed) matter excitation and the photons do not couple at low energies and iv) at large energy/momentum, the coupling becomes infinite. The arbitrarily large coupling strength at large momenta explains why, in the two limits of large detuning ($\omega_k \to 0$ and $\omega_k \to \infty$), two different asymptotic frequencies (ω_{TO} and ω_{LO} respectively) are obtained, i.e., it explains the

opening of the Reststrahlen band.

- In contrast, in the MoC model, (i) the coupling constant is independent of the photon energy, and (ii) the transverse optical phonon coincides with the bare matter excitation . In this case, the Reststrahlen band opens because the coupling is proportional to the time derivative of the oscillation amplitudes.

References

[1] C. Cohen-Tannoudji, J. Dupont-Roc, and G. Grynberg, *Photons and Atoms* (Wiley, New York, 1997).

[2] A. F. Kockum, A. Miranowicz, S. De Liberato, S. Savasta, and F. Nori, Ultrastrong coupling between light and matter. *Nat. Rev. Phys.* **1** 19-40 (2019).

[3] R. Guy Woolley, Power-Zienau-Woolley representations of nonrelativistic QED for atoms and molecules. *Phys. Rev. Research* **2**, 013206 (2020).

[4] D. L. Andrews, G. A. Jones, A. Salam, and R. Guy Woolley, Perspective: Quantum Hamiltonians for optical interactions. *J. Chem. Phys.* **148** 040901 (2018).

[5] R. H. Dicke, Coherence in spontaneous radiation processes. *Phys. Rev.* **93**, 99 (1954).

[6] S. Ribeiro, J. Aizpurua, and R. Esteban, Influence of direct dipole-dipole interactions on the optical response of two-dimensional materials in strongly inhomogeneous infrared cavity fields. *Phys. Rev. A* **108**, 043718 (2023).

[7] J. J. Hopfield, Theory of the contribution of excitons to the complex dielectric constant of crystals. *Phys. Rev.* **112**, 1555 (1958).

[8] D. Yoo et al., Ultrastrong plasmon-phonon coupling via epsilon-near-zero nanocavities. *Nat. Photonics* **15**, 125-130 (2021).
